# Selective Transesterification to Control Copolymer Microstructure in the Ring-Opening Copolymerization of Lactide and ε-Caprolactone by Lanthanum Complexes

**DOI:** 10.1021/acs.inorgchem.3c03120

**Published:** 2023-12-21

**Authors:** Bette Beament, Daniel Britton, Thomas Malcomson, Geoffrey R. Akien, Nathan R. Halcovitch, Michael P. Coogan, Rachel H. Platel

**Affiliations:** † Department of Chemistry, Lancaster University, Lancaster LA1 4YB, United Kingdom; ‡ Department of Chemistry, University of Manchester, Oxford Road, Manchester M13 9PL, United Kingdom

## Abstract

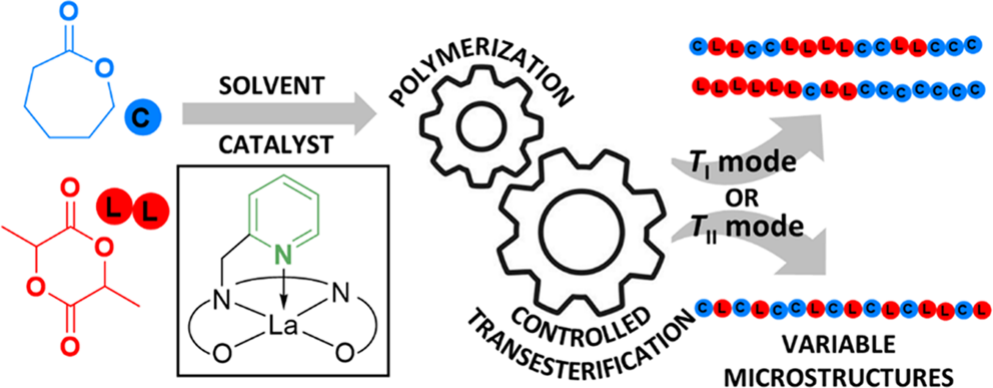

A series of novel lanthanum amido complexes, supported by ligands designed around the salan framework (salan = *N*,*N*′-*bis*(*o*-hydroxy, *m*-di-*tert*-butylbenzyl)-1,2-diaminoethane) were synthesized and fully characterized in the solid and solution states. The ligands incorporate benzyl or 2-pyridyl substituents at each tertiary amine center. The complexes were investigated as catalysts in the ring-opening homopolymerization of lactide (LA) and ε-caprolactone (ε-CL) and copolymerization of equimolar amounts of LA and ε-CL at ambient temperature. Solvent (THF or toluene) and the number of 2-pyridyl groups in the complex were found to influence the reactivity of the catalysts in copolymerization reactions. In all cases, complete conversion of LA to PLA was observed. The use of THF, a coordinating solvent, suppressed ε-CL polymerization, while the presence of one or more 2-pyridyl groups promoted ε-CL polymerization. Each copolymer gave a monomodal trace in gel permeation chromatography–size-exclusion chromatography (GPC-SEC) experiments, indicative of copolymer formation over homopolymerization. Copolymer microstructure was found to be dependent on catalyst structure and reaction solvent, ranging from blocky to close to alternating. Experiments revealed rapid conversion of LA in the initial stages of the reaction, followed by incorporation of ε-CL into the copolymer by either transesterification or propagation reactions. Significantly, the mode of transesterification (*T*_I_ or *T*_II_) that occurs is determined by the structure of the metal complex and the reaction solvent, leading to the possibility of controlling copolymer microstructure through catalyst design.

## Abstract 2

A series of lanthanum amido complexes incorporating salan-derived ligands containing 2-pyridyl groups are reported. Their behavior as catalysts in the homo- and copolymerization of lactide and ε-caprolactone is reported. Variable copolymer microstructures are obtained via a transesterification mechanism, and the mode of transesterification (*T*_I_ or *T*_II_) is found to depend on catalyst structure.

## Introduction

Biodegradable polymers continue to generate a high level of interest, offering an alternative to plastics derived from petrochemicals.^1^ In particular, the biodegradability and biocompatibility offered by some degradable polymers makes them attractive for use in pharmaceutical and medical applications; poly(lactic acid)s (PLAs)^2^ and poly(caprolactone) (PCL)^3^ fall into this category. An efficient method for preparing these aliphatic polyesters is through ring-opening polymerization (ROP) of a cyclic ester monomer (either lactide (LA), to form PLA, or ε-caprolactone (ε-CL) to form PCL) using a metal catalyst.^4−6^ It is widely accepted that the reactions proceed via a coordination–insertion mechanism. Through judicious choice of ligand(s) and metal, it is possible for these polymerizations to proceed in a highly controlled manner, often with very little or no transesterification side reactions, which scramble the polymer chains.

PLA, with a *T*_g_ of around 57 °C, has some attractive mechanical properties, in particular toughness, but it is brittle and has poor elasticity, as well as suffering from poor water and gas permeability.^7^ On the other hand, PCL (*T*_g_ −60 °C) has good elasticity and gas permeability, but less desirable mechanical properties.^8^ Thus, the two polymers can be considered complementary. A general strategy for tuning the properties of polymeric materials is copolymerization of two monomers. Depending on the relative reactivity of the two monomers with the catalyst, a variety of copolymers can be prepared: block, gradient, statistical, alternating, or intermediate microstructures. Although in block copolymers, the constituent monomer properties can be retained in each block, a statistical copolymer delivers properties that are intermediate between those of the two monomers. These are of interest for use in various medical applications.^9^ While the majority of reports disclosing copolymerization of LA and ε-CL involve the use of aluminum-based initiators,^10−19^ a variety of other metal initiators have been disclosed,^20−24^ as well as limited examples incorporating lanthanides.^25−28^ Generally, the much greater reactivity of LA compared to ε-CL makes the preparation of statistical copolymers challenging.^9^ Some strategies have proved successful in reducing the reactivity gap between LA and ε-CL: increasing steric bulk^10^ or rigidity^11,16,17^ of ancillary ligands, controlling the configuration of the active species with a chiral initiator,^13^ and selecting initiators that already have a very large reactivity difference that favors ε-CL homopolymerization.^26^ Another strategy is to exploit transesterification reactions, which scramble the polymer chains and can lead to statistical polymers.^23,24,27,28^ Notably, high temperatures are generally employed for this route, with one exception.^28^

While transesterification, the exchange of groups between different esters, is detrimental to polymer structure (broadening dispersity and reducing tacticity), it can provide a route to statistical copolymers. Some of the earliest examples of copolymers of poly(lactide-*co*-ε-caprolactone) were obtained by means of transesterification.^29−32^ Furthermore, in a copolymer with two different polymer units present, the *position* in the polymer chain where the transesterification occurs is also important. In particular, the fact that lactide is a cyclic diester monomer means that transesterification can scramble the monomer unit itself (i.e., a bond that was present in the monomer between the two lactyl units can be broken).

**Figure 1:**
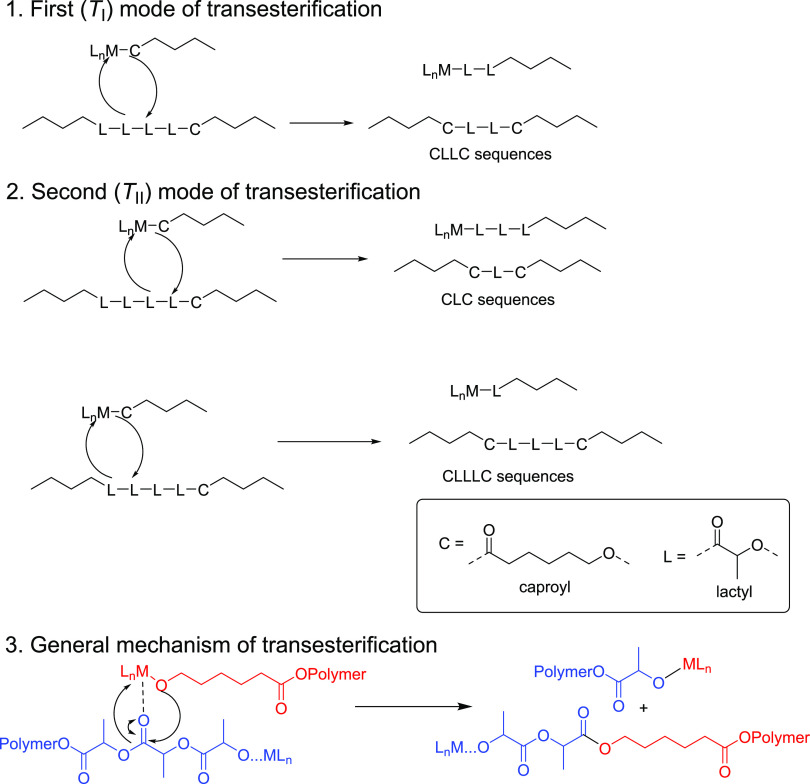
First (1) and second (2) modes of transesterification. L_n_ = ancillary ligand(s), M = metal. (3) General mechanism of intermolecular transesterification in poly(lactide-*co*-ε-caprolactone) synthesis.

In a significant contribution concerning poly(lactide-*co*-ε-caprolactone) synthesis, two different modes of transesterification were defined by Kasperczyk and Bero: the first (*T*_I_) maintaining “whole” lactide (LL) sequences, and the second (*T*_II_) leading to “anomalous” CLC sequences, i.e., those that cannot be formed via ring-opening as part of a chain growth polymerization (C = caproyl unit, L = lactyl unit, Figure 1(1 and 2)).^30^ The actual mechanism of transesterification for both *T*_I_ and *T*_II_ modes is identical: coordination of a carbonyl oxygen to lanthanum, attack of the metal alkoxide upon the carbonyl carbon forming a tetrahedral intermediate, reformation of the carbonyl moiety, and exchange of the ester/metal alkoxide group (Figure 1(3)). The difference between the *T*_I_ and *T*_II_ modes is the location of the bond-breaking: in the *T*_I_ mode, the bond that is broken leaves pairs or even numbers of lactyl units in a sequence; in the *T*_II_ mode, the bond that is broken leaves single, or odd numbers, of lactyl units in the copolymer chain. The mode of transesterification that occurs has been found to depend on the metal-initiating species.^33^ While acknowledging the complexity of the process, there is, to the best of our knowledge, no explanation for the differing levels of *T*_I_ and *T*_II_ transesterification with different metals. Thus, the process for quantifying transesterification is established, but the underlying factors affecting this process are far less well understood. Specifically, the complete preservation of LL sequences and copolymer regions containing even numbers of lactyl units during *T*_I_ transesterification is incompatible with a purely statistical process. In the recent literature, it is widely accepted that the occurrence of transesterification in poly(lactide-*co*-ε-caprolactone) synthesis is established by observation of the CLC sequence in copolymer ^13^C NMR spectra. However, this means that only the *T*_II_ mode of transesterification is identified. There are few instances where the *T*_I_ and *T*_II_ transesterification modes are distinguished,^22^ leading to the possibility of the occurrence of transesterification being missed unless the reaction is investigated in detail.

We are interested in controlling copolymerization and copolymer microstructure through lanthanide complex design and specifically wished to explore the influence of neutral donor groups in ancillary ligands. Hemilability is an attractive feature that has been used to good effect with lanthanides and rare-earth metals in ring-opening polymerization catalysis to tune reactivity.^34−41^ Recently, the role of neutral donors in isoselective ROP of *rac*-β-butyrolactone has been established.^42^

**Scheme 1:**
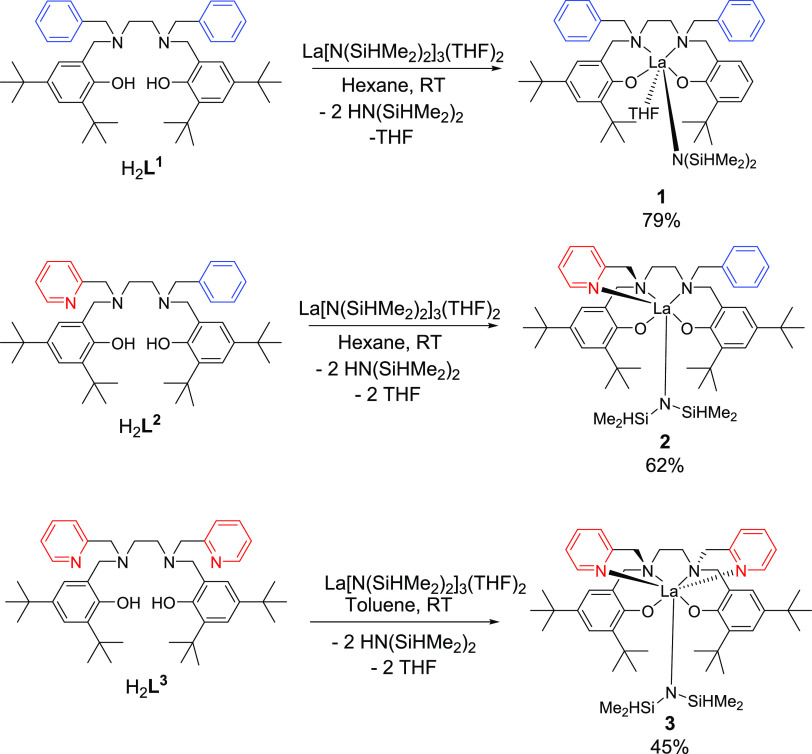
Synthesis of Complexes **1**–**3**

Although many lanthanide-based complexes have been reported for homopolymerization of LA, there are few examples of lanthanide catalysts for copolymerization of LA with ε-CL.^26−28,43^ The salan ligand framework was selected (salan = *N*,*N*′-*bis*(*o*-hydroxybenzyl)-1,2-diaminoethane) as a starting point, due to its prevalence in coordination chemistry and catalysis, modularity, and ease of functionalization.^44^ Three ligands were used in the study, incorporating 2-pyridyl or benzyl substituents on the nitrogen atoms and *tert*-butyl substituents in the ortho and para positions of the phenolate groups (Scheme 1). Related ligands have been explored previously in combination with titanium for the homopolymerization of *rac*-LA and ε-CL,^45^ with the neutral donors proposed to influence reactivity at the metal center.

## Results and Discussion

### Synthesis of **1**–**3**

Ligands H_2_**L**^**1**^ and H_2_**L**^**3**^ were prepared following modified literature procedures.^46,47^ H_2_**L**^**2**^ was prepared by a condensation reaction of *N*-benzyl ethylene diamine with pyridine-2-carboxaldehyde, reduction, and a double Mannich condensation reaction with 2,4-di-*tert*-butylphenol and formaldehyde and fully characterized by ^1^H and ^13^C NMR spectroscopy and mass spectrometry (ESI). The reaction of ligands H_2_**L**^**1**–**3**^ with 1 equiv of La[N(SiHMe_2_)_2_]_3_(THF)_2_ in either hexane or toluene at room temperature for 18 h gives colorless precipitates. Filtration under vacuum gave lanthanum complexes **1**–**3** in 45–79% yields (Scheme 1). The complexes were characterized by NMR spectroscopy, elemental analysis, and X-ray diffraction.

### Solid-state Characterization of **1**–**3**

**Figure 2:**
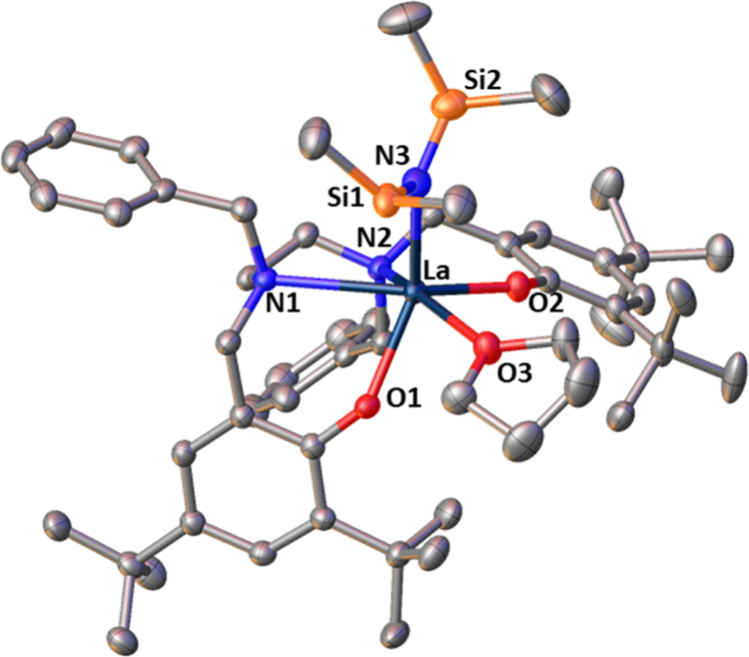
Molecular structure of complex **1**. Thermal ellipsoids are shown at 30% probability. Hydrogen atoms are removed for clarity. Selected bond distances (Å) and bond lengths (deg): La(1)–O(1) = 2.259(2), La(1)–O(2) = 2.287(2), La(1)–O(3) = 2.585(2), La(1)–N(1) = 2.715(3), La(1)–N(2) = 2.788(3), La(1)–N(3) = 2.428(3), O(1)–La–O(2) = 112.81(9), N(1)–La–N(2) = 66.27(8), Si(1)–N(3)–Si(2) = 129.11(19), Si(1)–N(3)–La(1) = 118.54(17), Si(2)–N(3)–La(1) = 112.23(17).

**Figure 3:**
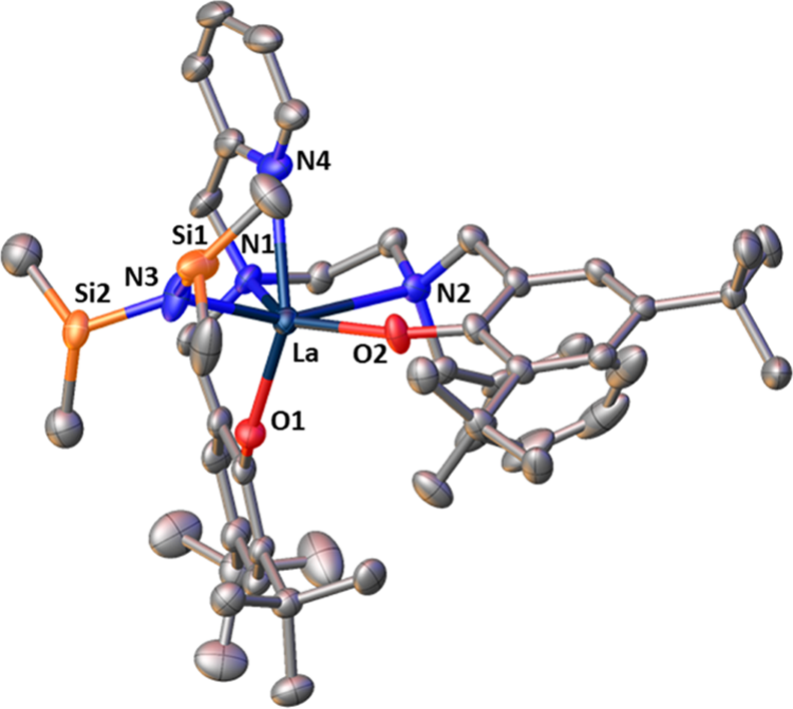
Molecular structure of complex **2**. Thermal ellipsoids are shown at 30% probability. Hydrogen atoms are removed for clarity. Selected bond distances (Å) and bond lengths (deg): La(1)–O(1) = 2.254(2), La(1)–O(2) = 2.246(2), La(1)–N(1) = 2.745(4), La(1)–N(2) = 2.740(5), La(1)–N(3) = 2.424(5), La(1)–N(4) = 2.644(5), O(1)–La–O(2) = 112.54(14), N(1)–La–N(2) = 66.68(13), Si(1)–N(3)–Si(2) = 115.7(3), Si(1)–N(3)–La(1) = 118.0(3), Si(2)–N(3)–La(1) = 124.6(3).

**Figure 4:**
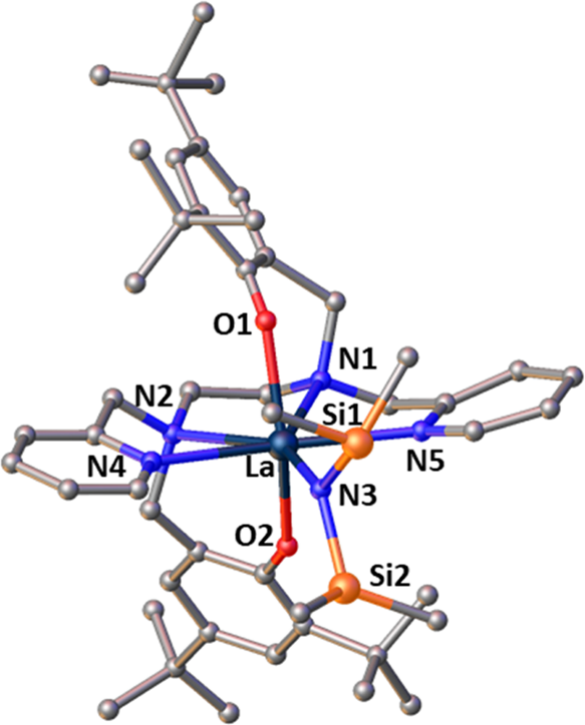
Molecular structure of complex **3** showing atom connectivity. Hydrogen atoms are removed for clarity.

Single crystals of the complexes were grown from hexane (**1** and **2**) or toluene (**3**) from solutions kept at −35 °C. Single-crystal X-ray diffraction studies show that **1** (Figure 2), **2** (Figure 3), and **3** (Figure 4) are all mononuclear species in the solid state. A lanthanum complex closely related to **1**, where methyl groups replace *tert*-butyl groups, adopts a dinuclear structure,^27^ suggesting that the *tert-*butyl groups in **1**–**3** provide sufficient steric bulk around the metal to prevent dimerization.

The La center in complex **1** is 6-coordinate, with distorted octahedral geometry (Figure 2). The complex can be described as having *cis*-β geometry based on the N_2_O_2_ salan ligand:^48,49^ N1, N2, and O2 of the tetradentate salan ligand occupy three equatorial positions and O3(THF) occupies the final equatorial position. O1(salan) and N3 (N(SiHMe_2_)_2_) occupy the apical positions. The angle between the mean planes N(1)–N(2)–O(1) and O(2) −La–N(1) is 74.69°. N(1) and N(2) have the expected tetrahedral geometry; the benzyl groups point away from the metal center, one angled up and one down with respect to the N(2)–N(1)–O(1)–O(3) plane. The La–O(1,2) and La–N(1,2) bond lengths are within the expected range for these bonds, when compared to other similar structures.^42,50−52^ The La–O(3) distance of 2.585(2) Å for **1** and the Ln–N(amido) distance of 2.428(3) Å are also within the range reported by others.

Complex **2** (Figure 3) also contains a six-coordinate lanthanum center and can also be described as having *cis*-β geometry based on the N_2_O_2_ salan ligand. In this case, the pyridyl nitrogen (N(4)) is designated an ancillary ligand and occupies one apical position and O1(salan) occupies the other apical position. The −N(SiHMe_2_)_2_ (N(3)) ligand lies trans to N(1). The angle between the mean planes N(1)–N(2)–O(1) and O(2) −La–N(1) is 75.09°. The La–N(4) bond length of 2.644(5) is similar to a lanthanum – N(pyridyl) bond length in a related structure.^25^

Detailed investigation of the solid-state structure of complex **3** (Figure 4) was somewhat hampered by disorder of the dimethylsilyl groups resulting in large thermal ellipsoids for the carbon atoms bound to silicone. However, it can clearly be observed that the La center is seven-coordinate, bound through the 4 nitrogen (N1, N2, N4, N5) and 2 oxygen atoms (O1, O2) in the salan ligand, plus the −N(SiHMe_2_)_2_ ligand. No THF is bound to the La center and the complex has *C*_2_ symmetry. The configuration of complex **3** is based on *cis*-α geometry: the phenoxide ligands (O1, O2) lie *trans* to each other and occupy the apical positions, and the pyridyl ligands (N4, N5), also mutually *trans*, occupy equatorial positions.

### Solution-State Characterization of **1**–**3**

In the solution state, complexes **1**–**3** were fully characterized using 1D and 2D NMR spectroscopic techniques. The ^1^H NMR spectra of complexes **1** and **2** both display significant fluxionality in solution at 298 K. In the ^1^H NMR spectrum of complex **1** at 298 K, there is a set of broad signals and a set of sharp signals, in a ratio of 1:4 (Figure S13). Heating the sample to 328 K has the effect of sharpening all of the signals. We suggest the exchange processes relate to the movement of the *N*-benzyl substituents and/or flexibility in the N–N ethylene diamine linker that is slow on the NMR time scale at ambient temperature.

In the ^1^H NMR spectrum of complex **2** at 298 K, the py-NC*H* proton appears sharp, as do the other py–C*H* protons, leading us to conclude that the fluxionality here arises from dynamic behavior associated with the benzyl group and/or flexibility in the ethylene diamine linker in the ligand rather than hemilability of the pyridyl group. Again, the fluxionality of complex **2** was investigated using variable-temperature NMR spectroscopy (Figure S14) and exchange between a major and minor species was observed.

For complex **3** the *C*_2_ symmetry adopted in the solid-state is reflected in the solution-state ^1^H NMR data, which contains a single set of sharp resonances (4 signals) for the pyridyl protons, two signals corresponding to the *tert*-butyl groups, and six resonances assigned to the ligand C*H*_2_ signals (Figure S11). Furthermore, the pyridyl NC*H* proton is shifted downfield from 8.39 ppm in the H_2_**L**^**3**^ ligand NMR spectrum to 9.38 ppm in **3**, indicating that the pyridyl nitrogen is coordinated to the metal.

^1^H DOSY NMR spectroscopy was used to rule out monomer–dimer equilibria being the cause of the solution-state fluxionality observed for **1** and **2**. Each complex displays a single diffusion coefficient (Figures S49–S51), and these are very similar for **1**–**3**, which suggests that only mononuclear species are present in solution.

^1^H–^15^N heteronuclear multiple bond coherence (HMBC) experiments were used to probe the coordination of the pyridyl ligand to lanthanum in **2**. The ^1^H–^15^N HMBC spectrum of complex **2** indicates there are four inequivalent nitrogen environments (Figure S15), as expected for the *C*_1_ symmetry of the complex. A signal at 315 ppm in the H_2_**L**^**2**^ spectrum is assigned to the pyridyl-*N*, and this signal shifts to 292 ppm in **2**, providing further evidence that the pyridyl nitrogen in **2** is coordinated to the metal.^53^

Finally, the behavior of **1**–**3** in the presence of triphenylphosphine oxide (TPPO) was investigated, to determine if the addition of this base would displace the pyridyl ligands in **2** and **3**. A single equivalent of TPPO was added to a solution of each complex in C_6_D_6_, and the resultant solution was studied by NMR spectroscopy. In the case of **1** and **2**, both the ^1^H and ^31^P{^1^H} NMR spectra suggest that TPPO coordinates to lanthanum, on the basis of chemical shift changes to signals in both the ^1^H NMR spectra and ^31^P{^1^H} NMR spectra compared to starting complexes and TPPO (Figure S52 and S53). Specifically, in the case of **2 +** TPPO, the ^31^P{^1^H} NMR spectrum contains a sharp signal at δ 34.8 ppm and a broad resonance at δ 25.5 ppm in an ∼5:1 ratio, compared to δ 24.9 ppm for TPPO (Figure S55). The chemical shift of the NC*H* pyridyl proton shifts from 8.52/8.78 ppm in **2** to 9.95 ppm in the Ph_3_P=O adduct, compared to 8.39 ppm in the free ligand (Figure S53). The fact that the NC*H* pyridyl proton shifts further downfield in comparison to H_2_**L**^**2**^ suggests that the presence of TPPO does not disrupt the pyridyl coordination in **2**. For complex **3**, however, the resonances in the ^1^H NMR spectrum assigned to **3** do not change at all upon addition of 1 equiv of TPPO and the ^31^P{^1^H} NMR spectrum of the reaction mixture contains a sharp signal at 25.1 ppm, comparable to the chemical shift for free TPPO (Figures S54, S55). This indicates both that pyridyl coordination in **3** is not disrupted by TPPO, and that TPPO does not coordinate to lanthanum when in the presence of **3**. In summary, both solid- and solution-state characterization data support discrete, mononuclear structures for **1**–**3**, in which the pyridyl groups, where present, remain coordinated to lanthanum with very little evidence for any hemilabile behavior.

### Homopolymerization of *rac*-LA and ε-CL

Complexes **1**–**3** were first tested as catalysts in the ROP of *rac*-LA and ε-CL in THF (Tables S1 and S2). All of the complexes were active, fully converting 200 equiv of monomer to polymer within minutes. Molecular weights were higher than calculated values, attributed to slow initiation by the −N(SiHMe_2_)_2_ group. Complexes **1** and **2** produced polymer with similar *M*_n_ (approximately 1.5 times higher than *M*_n_(calc) for PLA and 3 times higher than *M*_n_(calc) for PCL), while complex **3** produced polymer with a much higher *M*_n_ (approximately 6 times the calculated value for both polymers), which could reflect the greater steric congestion in **3** at the metal center afforded by the two 2-pyridyl groups. Dispersities ranged from 1.18 to 1.31. In *rac*-LA polymerization, the polymers obtained had a very slight heterotactic bias (*P*_r_ = 0.52–0.66). The fact that complexes **1**–**3** yield polymers with different molecular weights also strongly suggests that the pyridyl groups in **2** and **3** are not hemilabile. The ROP of ε-CL was also briefly explored in toluene, at [ε-CL]_0_ = 1 M and [ε-CL]_0_/[La] = 200 (Table S2). The reaction mixtures became highly viscous in <1 min in each case, and in ∼10 s in the presence of **3**. In this solvent, the *M*_n_ values for the polymer produced were all much higher than the *M*_n_(calc) of 28.8 kg mol^–1^. The gel permeation chromatography (GPC) traces obtained were also bi/multimodal, indicating a significant loss of polymerization control in the noncoordinating solvent.

### Copolymerization of LA and ε-CL

**Scheme 2:**
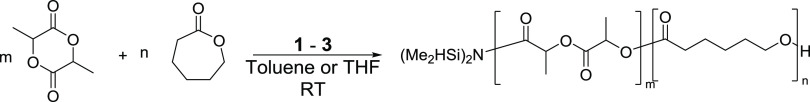
Copolymerization of *rac*-LA and ε-CL Initiated by **1**–**3**

Having established the high activity of **1**–**3** in homopolymerizations, we moved on to investigate the copolymerization of *rac*-LA or *S*,*S*-LA, and ε-CL (Scheme 2 and Table 1). A range of behavior was observed, depending on both the initiator structure and solvent used, demonstrating that initiator structure is key.

**Table 1:** Copolymerization of *rac*-LA or *S*,*S*-LA, and ε-CL Initiated by **1**–**3**^a^

entry	initiator [I]	LA	solvent	ε-CL/LA conv. (%)^b^	CL/LA (mol %)^b^	CL–LA/CL–CL (mol %)^c^	*L*_LL_/*L*_CL_^c^,^d^	*M*_n_ × 10^3^ (g mol^–1^)^e^	*M*_n_(calc) × 10^3^ (g mol^–1^)^f^	*Đ*^e^
**1**	**1**	*rac*-LA	toluene	13/99	13/87	78/22		39.0	15.8	1.44
**2**	**1**	*rac*-LA	THF	11/99	9/91	80/20		36.3	15.6	1.40
**3**	**2**	*rac*-LA	toluene	99/99	47/53	78/22	1.9/1.4	45.3	25.8	1.26
**4**	**2**	*rac*-LA	THF	23/99	17/83	79/21		36.8	17.0	1.30
**5**	**3**	*rac*-LA	toluene	99/99	57/43	29/71	5.0/2.9	69.3	25.8	1.34
**6**	**3**	*rac*-LA	THF	99/99	49/51	50/50	3.1/1.8	71.3	25.8	1.26
**7**	**1**	*S*,*S*-LA	toluene	12/99	12/88	62/38		33.1	16.1	1.30
**8**	**1**	*S*,*S*-LA	THF	11/99	12/88	80/20		38.7	15.7	1.25
**9**	**2**	*S*,*S*-LA	toluene	99/99	51/49	75/25	1.9/1.6	52.0	25.8	1.23
**10**	**2**	*S*,*S*-LA	THF	20/99	19/81	66/34		41.1	17.3	1.20
**11**	**3**	*S*,*S*-LA	toluene	99/99	52/48	22/78	8.9/3.4	57.6	25.8	1.33
**12**	**3**	*S*,*S*-LA	THF	99/99	46/54	46/54	3.2/2.1	68.5	25.8	1.28

a General polymerization conditions: [LA]_0_ = [CL]_0_ = 1.0M, ([LA]_0_ + [CL]_0_)/[I] = 200, 25 °C, 3 h. Each entry is representative of at least 2 reactions.

b Determined by ^1^H NMR spectroscopy at 400 MHz in CDCl_3_.

c Average sequence length of lactidyl (*L*_LL_) and caproyl (*L*_CL_) units determined by ^13^C NMR spectroscopy at 100 MHz in CDCl_3_.

d A blank entry indicates the values were not calculated due to the very low conversion of ε-CL.

e Number-average molecular weight (*M*_n_) and dispersity (*Đ* = *M*_w_/*M*_n_) determined by GPC-multiangle laser light scattering (MALLS) at 40 °C in THF using d*n*/d*c* values of 0.079 and 0.042 for PCL and PLA, respectively, and using d*n*/d*c* (copolymer) = (0.079 × CL molar ratio/100) + (0.042 × LA molar ratio/100).

f *M*_n_(calc) = {[114([ε-CL]_0_/[I]) × Conversion_CL_(%)/100] + [144[*rac*-LA]_0_/[I]_0_ × Conversion_LA_(%)/100]}.

### Monomer Conversion

For complex **1**, while the *rac*-LA reached full conversion over the 2 h reaction time, ε-CL only reached very low conversions regardless of the solvent used (13% in toluene and 11% in THF; Table 1, entries 1 and 2). The observation of far lower reactivity of ε-CL in the presence of LA than in the absence of LA has precedence.^9^ Full conversion of both monomers was achieved using **2** in toluene (entry 3), while THF hinders the incorporation of ε-CL into the copolymer (entry 4) for this initiator. For complex **3**, regardless of solvent used, full conversions of both *rac*-LA and ε-CL were observed (entries 5 and 6). In terms of both monomer conversion and copolymer composition, there is no difference between using *rac*- or *S*,*S*-LA for **1**–**3**, which is to be expected given the absence of any significant stereocontrol in *rac*-LA homopolymerizations. Each polymer produced a monomodal trace in GPC analysis, showing that copolymerization, rather than separate homopolymerizations, occurs (Figures S32–S43). Generally, the molecular weights follow the trend observed in homopolymerizations: for complexes **1** and **2***M*_n_ values are approximately twice the calculated values, whereas for complex **3** they are around 3 times the *M*_n_(calc) value. Nevertheless, the copolymers had narrow to moderate dispersities, suggesting that **1**–**3** are capable of mediating controlled copolymerizations of these monomers. Remarkably, the dispersities of copolymers prepared in either solvent are comparable and the poor polymerization control in ε-CL homopolymerization in toluene is not observed in the copolymerization.

Clearly, both the initiator structure and solvent heavily influence the ability to incorporate ε-CL into the copolymers. The presence of at least one pyridyl group in the ancillary ligand is crucial to achieving full conversion of both monomers. In complex **1**, incorporating a tetradentate ligand, it is possible that the LA reacts faster than the ε-CL, and the 5-membered chelate formed by κ^2^-coordination of the last-inserted *rac*-LA monomer to lanthanum severely hinders/prevents insertion of ε-CL. For complex **2**, the additional pyridyl group in the ligand facilitates full ε-CL conversion in a noncoordinating solvent, suggesting the additional electron density afforded by the pyridyl group disrupts the coordination of LA (either via weakening of the La–O-lactate bond or through steric effects) and allows incorporation of ε-CL. The use of THF as a solvent leads to a very low conversion of ε-CL, likely due to the competitive coordination of THF with ε-CL at lanthanum. For complex **3**, the hexadentate ligand containing two pyridyl groups increases the electron density at lanthanum considerably, as well as potentially disrupting the κ^2^-coordination of LA through the creation of steric congestion.

### Copolymer Characterization

**Figure 5:**
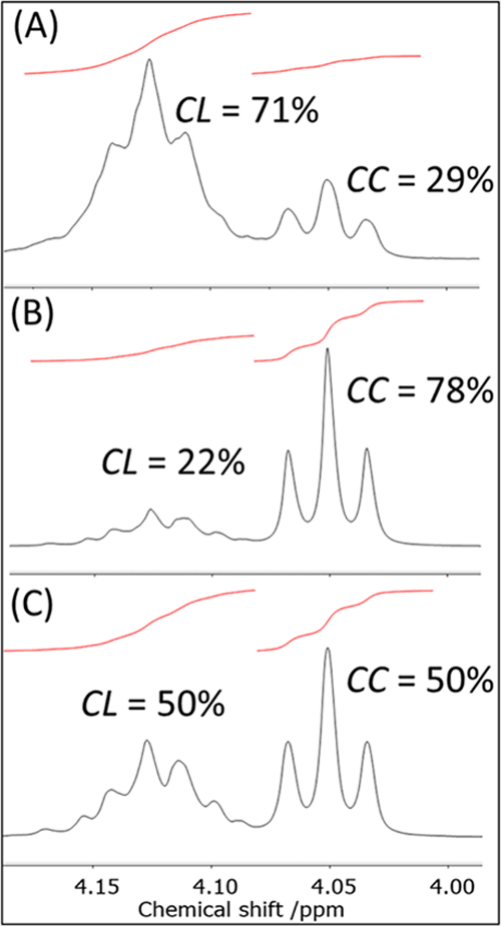
Detail of the ^1^H NMR spectra of copolymers formed by **2** in toluene (A), **3** in toluene (B), and **3** in THF (C), showing the relative integrations of signals from the −OC*H*_2_– caproyl unit protons. *CL* denotes a caproyl unit (*C*) lying next to a lactyl unit (*L*); *CC* denotes a caproyl unit (*C*) lying next to another caproyl unit (*C*).

Where full conversion of both monomers occurs (entries 3, 5–6, 9, 11–12), the copolymers were fully characterized by NMR spectroscopy, GPC, differential scanning calorimetry (DSC), and in some cases, matrix-assisted laser desorption/ionization time-of-flight mass spectrometry (MALDI-TOF MS). All of the data suggested that copolymers, rather than homopolymers, are formed. The ^1^H and ^13^C NMR spectra of copolymers were assigned by comparison with literature data.^31,44^ The chemical shift of the caproyl unit −OC*H*_2_– protons is sensitive to the nature of the neighboring unit; thus, the signal for a C*L* diad (a caproyl unit (*C*) next to a lactyl unit (*L*), 4.12 ppm) is distinct from C*C* diad (a caproyl unit lying next to a caproyl unit, 4.04 ppm, Figure 5). The polymers’ compositions, determined by ^1^H NMR spectroscopy, closely match the monomer feed ratio, with all three copolymers composed of approximately 50% of each monomer. The presence of heterodiads and heterotriads in ^1^H and ^13^C NMR spectra, respectively, is evidence for copolymer formation. The fact that the copolymers give rise to a monomodal trace in GPC is further strong evidence for copolymer formation. Some copolymers were additionally characterized by ^1^H DOSY NMR spectroscopy, and these display a single diffusion coefficient, as expected for a copolymer rather than a mixture of homopolymers (Figures S56–S64). Analysis of copolymers using differential scanning calorimetry (DSC) was carried out to determine their glass transition temperatures (*T*_g_, Table S4). Each copolymer displayed a *T*_g_ that was intermediate between that of the constituent homopolymers and in excellent agreement with the predicted value based on copolymer composition calculated using the Fox equation.^54^ This also indicates that copolymers rather than homopolymers have been prepared. The copolymer prepared using complex **3** in toluene displayed two glass transitions: one at −27.9 °C (intermediate between PCL and PLA), and a second at 50.1 °C, close to the *T*_g_ of PLA. This suggests some microphase separation occurs in PLA-rich regions of the copolymer,^55^ and is reflective of the longer average sequence lengths of each monomer.

### Copolymer Microstructure

**Figure 6:**
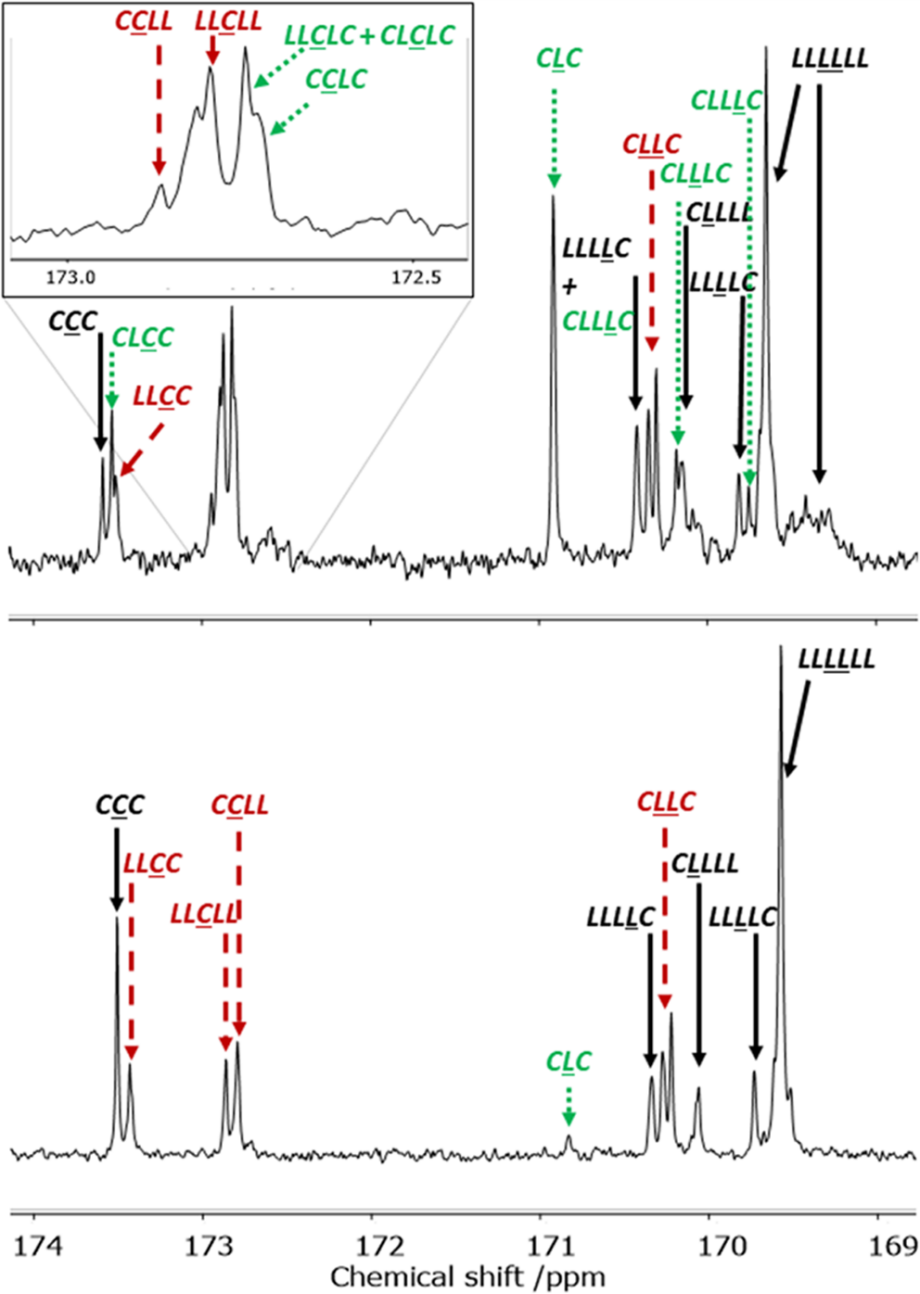
Carbonyl region in the quantitative ^13^C NMR spectra of copolymers formed in the copolymerization of *S,S*-LA and ε-CL by complex **2** in toluene (Table 1, entry 9; top spectrum) and complex **3** in THF (Table 1, entry 12; bottom spectrum). Signals relating to various monomer sequences are labeled: sequences not necessarily arising from transesterification (solid arrows, black), sequences arising from *T*_I_ mode of transesterification (long dash arrows, red), sequences arising from *T*_II_ mode of transesterification (short dash arrows, green). C = caproyl unit (−C(O)(CH_2_)_5_O−), L = lactyl unit (−C(O)CH(CH_3_)O−), LL = latidyl unit (−C(O)CH(CH_3_)OC(O)CH(CH_3_)O−).

Quantitative ^13^C NMR spectra of copolymers provide more detailed insight into the microstructure of the copolymers since data at a triad level can be extracted. In particular, the signals in the carbonyl region were assigned using literature data (Figure 6) and average sequence lengths of lactide (*L*_LL_) and caprolactone (*L*_CL_) sequence lengths of each monomer unit were determined (Table 1).^31,33^

Despite almost identical overall compositions, the copolymer microstructures are all different. For complex **1** in toluene, 79% of *C* units lie next to *L* units (although since the composition of the copolymer is only 12% CL this is not remarkable). For complex **2** in toluene, the data suggest that a copolymer with a microstructure intermediate between statistical and alternating is formed: 71% of *C* units lie next to *L* units, with *L*_LL_ = 1.9 and *L*_CL_ = 1.4 (*L* = 2 for a statistical copolymer and *L* = 1 for an alternating copolymer). For complex **3** in toluene, just 22% of *C* units lie next to *L* units, which leads to longer average sequence lengths (*L*_LL_ = 5.0 and *L*_CL_ = 2.9), and a copolymer with a blockier microstructure. For complex **3** in THF, the copolymer structure is close to statistical; 50% of *C* units lie next to *L* units and 50% next to *C* units. These differences are also reflected in the quantitative ^13^C NMR spectra (Figure 6). For **2** and **3**, the only difference between use of *rac*-LA and *S,S*-LA is longer average sequence lengths, which has been observed by others.^32^ These data are suggestive of an opposite effect of coordinating solvent (which reduces ε-CL incorporation) and increasing number of pyridyl groups (which lead to increased ε-CL incorporation into the copolymers).

### Reaction Mechanism

The only way to fully understand the relative reactivity of monomers is through the calculation of reactivity ratios. However, it was found that all of the LA monomer is consumed within a very short space of time (<5 min), while the ε-CL remains largely unpolymerized during this time (up to ≈5% conversion) for both **2** and **3**. No sensible reactivity ratio data could be extracted, indicative of a nonideal copolymerization process. Instead, the progress of an ε-CL/*rac*-LA copolymerization reaction initiated by **3** in THF was followed through removal of aliquots from the reaction mixture at regular intervals, and analysis by ^1^H NMR spectroscopy to determine monomer conversion and proportion of CL-LA/CL-CL linkages (Table 2).

**Table 2:** Time Monitored Copolymerization of ε-Caprolactone and *rac-*Lactide Promoted by Complex **3**^a^

entry	time (min)	ε-CL/*rac*-LA conv. (%)^b^	CL/LA (mol %)^b^	CL–LA/CL–CL (mol %)^b^	*L*_LL_/*L*_CL_^c^,^d^
**1**	5	5/99	3/97	85/15	
**2**	15	15/99	10/90	64/36	
**3**	30	18/99	14/86	57/43	13.4/1.7
**4**	40	20/99	15/85	57/43	13.6/2.0
**5**	50	25/99	16/84	62/38	13.4/1.7
**6**	60	48/99	25/75	56/44	8.5/1.7
**7**	70	58/99	28/72	51/49	6.9/2.0
**8**	80	50/99	25/75	58/42	6.9/1.7
**9**	95	83/99	34/66	53/47	5.1/2.0
**10**	105	80/99	43/57	46/54	4.4/2.1
**11**	110	81/99	37/63	52/48	4.9/1.9
**12**	120	80/99	36/64	54/46	4.1/1.8

a General polymerization conditions: THF as a solvent, [LA]_0_ = [CL]_0_ = 0.5 M, room temperature, ([LA]_0_ + [CL]_0_)/[I] = 400, complex **3** as the initiator.

b Determined by ^1^H NMR spectroscopy.

c Average sequence length of the lactidyl (*L*_LL_) and caproyl (*L*_CL_) units as determined by ^13^C NMR spectroscopy at 100 MHz in CDCl_3_.

d A blank entry indicates the values were not calculated due to a very low conversion of ε-CL.

The data show a transesterification mechanism operates, whereby all of the LA is first polymerized as a block, before the reaction of ε-CL, which is incorporated into the polymer via transesterification. After 5 min, *rac*-LA is 99% converted, and remains at this level of conversion throughout the remainder of the reaction; ε-CL is 5% converted after 5 min. The percentage conversion of ε-CL then increases over the subsequent 100 min to reach 80% conversion and a copolymer composition of 43% CL/57% LA. The ratio of C*L*:C*C* linkages also changes over the course of the reaction. After 5 min, this ratio is 85:15, i.e., of the 5% ε-CL incorporated into the copolymer, 85% of that lies next to a lactyl unit. As the percentage conversion of ε-CL increases, the C*L*:C*C* ratio decreases, to 62:38 at 25% conversion of ε-CL (at 50 min) and 56:44 at 48% conversion of ε-CL (at 60 min). This ratio is then approximately maintained until the reaction is quenched (120 min). Average sequence length data was calculated from quantitative ^13^C NMR spectra, showing that *L*_LL_ is very high in the early stages of the reaction (*L*_LL_ = 13.4 after 30 min), and decreases over the course of the reaction, while *L*_CL_ remains in the range of 1.7–2.0 throughout the reaction. If no transesterification were occurring, a block copolymer would result, with close to 100% C*C* linkages and no C*L* linkages detectable by ^1^H NMR.

Copolymerization via a transesterification mechanism is known in the literature: there are examples of poly(lactic acid–*co*–caprolactone) being prepared by this route.^23,24,27,28^ However, with one exception,^28^ high temperatures are always necessary, whereas here this behavior is observed at room temperature, highlighting the role of the pyridyl ligand substituents in **2** and **3** as facilitators of this reactivity.

**Scheme 3:**
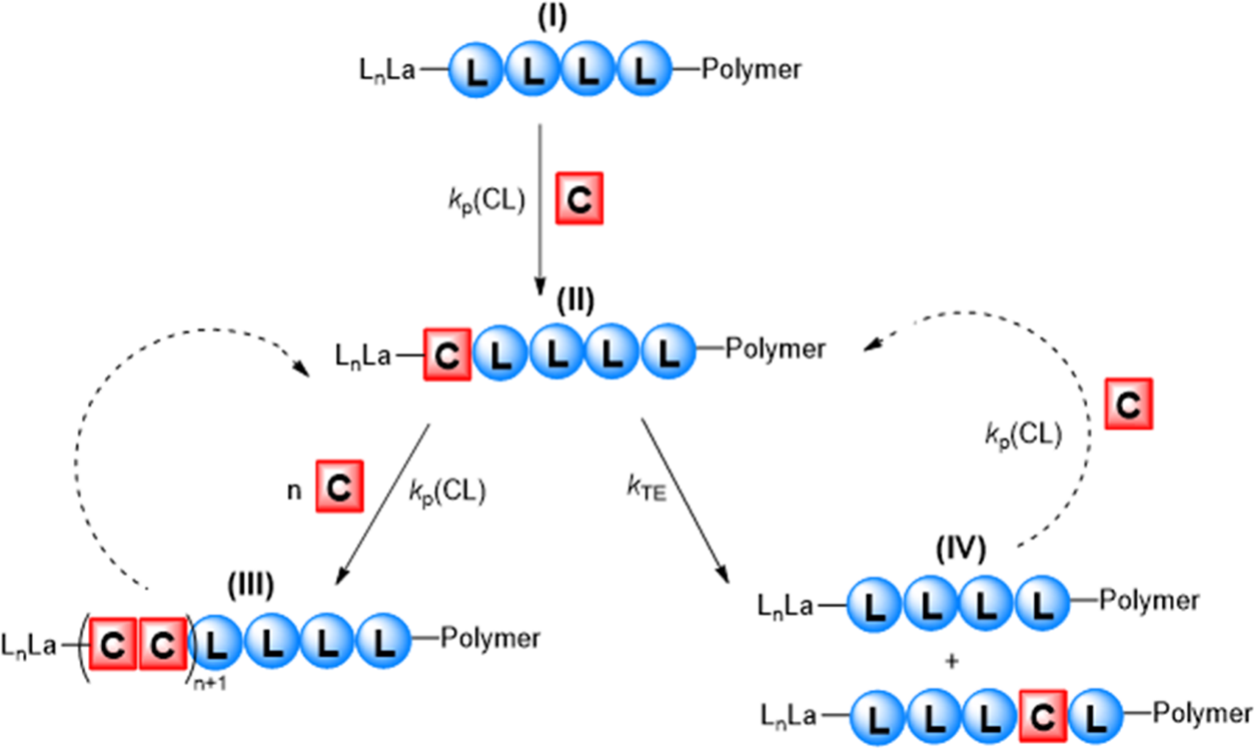
Mechanism of Copolymerization of LA and ε-CL by **2** and **3** Initially, all of the LA is polymerized (I). After ring-opening of ε-CL (II), either further ROP of ε-CL occurs (III), governed by *k*_p_(CL); or transesterification with an LA-rich polymer occurs (IV), governed by *k*_TE_.

The differing microstructures of the copolymers can be explained through consideration of the relative rates of the two competing processes occurring during the reaction once all LA monomer has been consumed: the propagation of ε-CL ROP (*k*_p_(CL)), and transesterification (*k*_TE_, Scheme 3). For **2**, the rate of transesterification (*k*_TE_) is very high compared to the rate of ε-CL propagation (*k*_p_(CL)), meaning that upon insertion of ε-CL into the L_n_La–L–L–polymer chain, it immediately undergoes transesterification and is incorporated into an L–L bond of another polymer chain. Only 6% of the caproyl carbonyl signal corresponds to the *C*C*C* sequence, while 60% of caproyl units are in LCL sequences, implying a relative *k*_TE_ much higher than *k*_p_(CL). The increased electron density at lanthanum and/or the steric bulk provided by the ancillary ligand in **3** (vs **2**) causes an increase in *k*_p_(CL) relative to *k*_TE_ for **3** and longer sequences of PCL are formed; 53% of the caproyl carbonyl signal corresponds to a *C*C*C* sequence. We attribute this to the weakening/reduction in the polarity of the La–O(lactate) bond and a more sterically congested metal center in **3** vs **2**, promoting ε-CL propagation over transesterification. When using complex **3** in THF, the solvent may disrupt the coordination of pyridyl groups to the metal, causing *k*_p_(CL) and *k*_TE_ to be similar (*C*C*C* makes up 35% of the caproyl carbonyl signal; in a statistical copolymer this would be 25%).

In summary, the rates of copolymerization of LA and ε-CL show a marked change from that observed in homopolymerizations, with LA essentially being consumed before ε-CL starts to react (while in the homopolymerizations the rates of polymerization of both monomers are comparable or activity in ε-CL ROP may be higher). Once LA is consumed and ε-CL starts to react, two competing processes, transesterification (governed by *k*_TE_) and propagation (governed by *k*_p_(CL)), occur, leading to different microstructures. However, the fact that the rate of ε-CL consumption is measurable over tens of minutes (compared to much more rapid homopolymerization) suggests that the polymerization process is different from that of homopolymerization. The rates of these competing processes, and thus the microstructures, are a function of the initiator structure and solvent. It is notable that where neutral donor ligands affect transesterification, suppression or reduction of transesterification events tends to be observed;^34^ here, the relationship between the presence of neutral donors and the occurrence of transesterification is more nuanced.

Computational calculations were carried out on complexes **1**–**3** to establish whether differences in their electronic structure could explain the differing initiator behaviors observed experimentally. Complexes were analyzed both in the absence of monomer and after the insertion of one equivalent of LA into the La–N(SiHMe_2_)_2_ bond. Analysis of the natural bonding orbitals (NBOs, Figure S65) shows minimal metallic character in the orbitals corresponding to ligand–metal bonding interactions, as expected from the stereotypically weak ligand-field interactions formed with lanthanide ions. Additionally, the frontier orbitals (Figure S66), show consistent highest occupied molecular orbital (HOMO) character on the metal-bound pyridyl moiety of complexes **2** and **3**, with lowest unoccupied molecular orbital (LUMO) character situated on the metal-coordinated phenol moiety. Together, this suggests minimal electronic differences in the interactions between the lanthanum and the initiating group in **1**–**3**. In an attempt to further characterize the metal–ligand interactions present, QTAIM metrics were analyzed for each complex (Table S5). There is minimal variation in any QTAIM parameters between the complexes in the absence of monomer. An exception is noted for the ellipticity, where large percentage changes are observed. However, these values show negligible absolute change relative to what is considered to represent a significant change in the bonding character. The same lack of variation between complexes is observed after reaction with one equivalent of LA (Tables S5, S6), with the exception of the NBO charges of the lanthanum and the coordinating oxygen of the lactone. While small, the differences between these charges are notable and may suggest that discovery of the determining factor in the catalytic behavior of these complexes lies not in their native electronic structure but rather in their physical accommodation of the intermediates and transition states required to undergo ROP.

We also sought to distinguish between the two modes of transesterification. In the ^13^C NMR spectrum of poly(lactic acid–*co*–caprolactone), a signal at 170.8 ppm is assigned to the *C*=O in the *C*L*C* sequence.^31^ This sequence can only be formed through the *T*_II_ mode of transesterification. In the polymers obtained by **2** in toluene, this signal is very prominent, accounting for 14% of the total lactate carbonyl *C* signal. Furthermore, peaks for related sequences that would arise from the *T*_II_ mode of transesterification (*CL*C*C*, *LL*C*LC*, *CL*C*LC*, *CL*C*LL*) are present (Figure 6). Signals that could only arise from the *T*_I_ mode of transesterification (*CLLC*, at 170.20 and 170.15 ppm) are also present, indicating that both *T*_I_ and *T*_II_ modes of transesterification operate for **2**.

In the copolymers formed by **3**, the formation of 49% C*L* linkages after initial full conversion of all *rac*-LA monomers can only be explained by a transesterification mechanism. A closer examination of the ^13^C NMR spectrum revealed that the *C*L*C* sequence signal is barely visible, comprising 1.5% of the total lactate carbonyl *C* signal. But it is notable that signals indicating the presence of *CLLC* sequences are present and represent 20% of the lactate carbonyl *C* signal. Signals for the *LL*C*C* and *C*C*LL* sequences also each contribute 18% of the caproyl carbonyl signal. In the case of **3**, since LL sequences are preserved, the *T*_I_ mode of transesterification must be almost solely responsible. This suggests that the *type* of transesterification can be controlled through the catalyst structure.

A reaction was conducted whereby equimolar amounts of two active homopolymer chains (PLA and PCL) were mixed and left to react (Scheme S1). Even after 24 h, the resultant mixture consisted of the homopolymers and there was no evidence for the occurrence of any transesterification (Figures S45 and S46). This result strongly suggests that the transesterification occurs in tandem with propagation at the active metal caproyl site, immediately after insertion of one or more CL units into the polymer chain, rather than as an uncontrolled process.

The exact nature of the metal-lactate interaction was probed by a stoichiometric reaction of **3** with ethyl-(*S*)-lactate. In particular, it was important to establish whether the presence of the lactate moiety influences the behavior of the pyridyl groups. Complex **3** was reacted with 1 equiv. ethyl-(*S*)-lactate in an NMR scale reaction in benzene-*d*_6_. Although the resulting ^1^H NMR spectrum is broad, the signal arising from the pyridyl NC*H* protons shifts from a single doublet at 9.38 ppm in **3** to 2 doublets at 9.32 and 9.28 ppm after the addition of ethyl-(*S*)-lactate (Figure S47). The number of signals in the aromatic region for this species also increases, confirming the loss of the *C*_2_ symmetry **3** exhibits in solution. The decrease in the chemical shift for this signal, to a chemical shift intermediate between the free ligand (8.37 ppm) and the metal-bound complex, could imply that the pyridyl groups are hemilabile with the addition of the lactate ligand. The ^13^C {^1^H} NMR spectrum of the reaction mixture contains a broad signal at 185.4 ppm, assigned to the ethyl lactate carbonyl carbon (Figure S48). When compared to free ethyl-(*S*)-lactate (176.2 ppm) the downfield chemical shift is quite large, suggesting that the carbonyl group does coordinate to the lanthanum center, However, the broadness of the resonance does not rule out some dynamic behavior involving this carbonyl moiety.

Low molecular weight samples of copolymer were prepared by conducting polymerization reactions at a low monomer: catalyst ratio. ^1^H and ^13^C NMR spectra of the copolymers confirmed that their microstructure was identical to that in copolymers prepared at a higher catalyst:monomer ratio. The MALDI-ToF spectra obtained of these copolymers were complex, due to the presence of two monomers, and the possibility of both lactyl (L) and lactidyl (LL) units being present. The spectrum from polymer prepared using **2** in toluene contains signals corresponding to both polymer chains composed of [H–(*x*L + *y*CL)–N(SiHMe_2_)_2_]K^+^ and also [H–(*x*LL + *y*CL)–N(SiHMe_2_)_2_]K^+^, i.e., there is evidence that transesterification breaks the lactidyl unit (Figure S43). There is also a series of signals present, of lower intensity, that correspond to cyclic copolymer: [(*x*L + *y*CL)]K^+^ and [(*x*LL + *y*CL)]K^+^, again both with odd and even numbers of lactyl units. We therefore cannot rule out the formation of cyclic polymers at a higher catalyst:monomer ratio.

Comparison of this spectrum to those from catalyst **3** (Figure S44), where the evidence from ^13^C NMR spectroscopy confirms that CLC sequences are not formed in copolymers, reveals remarkably similar spectra (i.e., the presence of both lactyl and lactidyl units), suggesting that the overall composition of polymer chains in terms of monomer units is very similar for **2** and **3**. Evaluated alongside the ^13^C NMR spectroscopic data they indicate that, at least at low catalyst: monomer ratios, transesterification reactions are significant for both **2** and **3**. However, as shown by NMR spectroscopy, the *sequence* in which monomers are arranged in the polymer chains is quite different, depending on the catalyst and the reaction solvent used. Given that the LA fully reacts before ε-CL reaction begins, it is proposed that transesterification reactions during the LA homopolymerization process generate polymer chains containing both even and odd numbers of lactyl units, and it is from this starting point that the incorporation of ε-CL commences. Thus, the initial ε-CL unit can insert at any point on the polymer chain, and LL units are not necessarily preserved. However, for catalyst **3**, subsequent ε-CL units are only inserted at intervals of 2 L units (i.e., LL) from the initial C unit on a given polymer chain, while for catalyst **2**, there is no such limitation.

Consideration was given to the different modes of transesterification promoted by **2** (*T*_I_ and *T*_II_) and **3** (*T*_I_ only), and how this behavior relates to their very similar structures. Specifically, we observe that with **3**, LL sequences in the polymer remain intact. This means that some ester linkages in the polymer must react preferentially as ε-CL is incorporated into a PLA block in a transesterification process which is currently understood to be statistical. From this study, it is clear that small changes in ligand structure can cause one mode of transesterification to be strongly favored, and produce copolymers with very different microstructures.

## Conclusions

In conclusion, the synthesis and characterization of three salan-supported lanthanide complexes are reported, which incorporate additional 2-pyridyl groups. The characterization data supports complex structures whereby the 2-pyridyl groups, where present, are metal-coordinated in both the solid and solution states. The complexes were explored as initiators in the ring-opening copolymerization of *rac*-LA and ε-CL under ambient conditions. Both solvent (coordinating or noncoordinating) and initiator structure, namely, the number of 2-pyridyl groups present in the ligand, heavily influence the reactivity of the complexes as initiators. In particular, without the presence of at least one 2-pyridyl group in the ligand, very little incorporation of ε-CL into the copolymer occurs (complex **1**). With either one (**2**) or two (**3**) 2-pyridyl moieties, copolymers with compositions that are close to the monomer feed can be obtained under ambient conditions (room temperature).

The copolymer microstructure is tunable via initiator design and solvent choice, with the possibility to prepare copolymers tending toward alternating (complex **2** in toluene), blocky (complex **3** in toluene) or statistical (complex **2** in THF). The reaction mechanism was probed by determining both the consumption of ε-CL and copolymer composition over time, revealing that a transesterification mechanism operates. All of the lactide is polymerized very rapidly (within 5 min) and the ε-CL is then incorporated into the polymer through ROP and transesterification. The microstructure of the copolymer is determined by the relative rates of ε-CL propagation and transesterification, which in turn are controlled by ligand structure. Importantly, we have shown that catalyst design can control the *type* of transesterification (*T*_I_ or *T*_II_), as well as its extent.

There is good evidence that copolymer microstructure is crucial to copolymer degradation and that very minor changes in monomer sequence can have a large impact upon degradation behavior.^56,57^ Here, we highlight both the potential to access controlled (room temperature) transesterification and its use as a route to control monomer sequence in copolymers. Some key considerations for catalyst design are explored: the use of 2-pyridyl groups to vary metal coordination number and electronic environment. Given the increased reactivity observed of ε-CL in the presence of LA, it is possible that the strategy of incorporating neutral donors in ancillary ligands could provide a more general method to tune copolymer microstructure in cyclic ester copolymers.

## Experimental Section

### Materials

Catalysts **1**–**3** and all polymers and copolymers were prepared under an inert (nitrogen) atmosphere using standard Schlenk or glovebox (Innovative Technologies) techniques. Dry solvents (toluene, hexane, tetrahydrofuran) were obtained from an Inert SPS (solvent purification system). All solvents and chemicals were purchased from commercial suppliers (Sigma-Aldrich, Strem, Alfa Aesar, TCI) and used as received except where otherwise specified. *Rac*-LA and (*S,S*)-LA were recrystallized once from dry toluene and sublimed once under vacuum at 50 °C. ε-CL was stirred over CaH_2_ under N_2_ at 50 °C overnight, distilled under vacuum, and degassed via 3 freeze–pump–thaw cycles. Benzene-*d*_6_ and toluene-*d*_8_ were dried over activated 4 Å molecular sieves and degassed via 3 freeze–pump–thaw cycles. Ligands H_2_L^1^,^46^ H_2_L^3^,^45^*N*-benzyl-*N*′-(2-pyridinyl)-1,2-ethylene diamine,^58^ and [La(N(SiHMe_2_)_2_)_3_(THF)_2_]^59^ were prepared following literature procedures.

### Methods

^1^H and ^13^C NMR spectra were recorded at 400.1 and 100.6 MHz, respectively, on a Bruker Avance III 400 MHz spectrometer equipped with a broadband-observe probe (BBFO). ^1^H NMR spectra are referenced to residual solvent signals; ^13^C NMR spectra are referenced to the signal for the solvent ^13^C nucleus. The temperature was calibrated using deuterated methanol, and unless stated otherwise, were all acquired at 298.0 K. Spectra were processed using ACD/NMR Processor. Chemical shifts are reported in ppm and coupling constants in Hz. ^1^H–^15^N HMBC experiments were recorded on Bruker Avance III 400 and Avance III 700 spectrometers for complete characterization of complex **2**. These experiments (pulse sequence hmbcgpndqf, optimized for *^n^*J_NH_ 5 Hz) were used to obtain the ^15^N chemical shifts. For quantitative ^13^C NMR experiments, the *T*_1_ of representative poly(CL-*co*-LA) copolymer samples was measured using inversion recovery experiments. The longest *T*_1_ was determined as 6 s. The quantitative 1D ^13^C spectra were run with inverse-gated decoupling and a relaxation delay of 30 s.

X-ray data was collected on an Agilent Supernova Dual AtlasS2 diffractometer. Suitable crystals were mounted on a Mitegen loop using Paratone-N oil. Structures were solved using Olex2;^5^ structure solution was achieved with the ShelXT program using intrinsic phasing.

Molecular weights of polymers were determined by gel permeation chromatography (GPC) multiangle laser light scattering (MALLS) in chloroform using a Shimadzu liquid chromatograph equipped with a Shimadzu LC-20AD pump and autosampler, two Phenogel 5 μm linear (2) columns (300 × 7.8 mm), a Shimadzu RID-20A refractive index detector, a Wyatt miniDAWN treos LLS detector, and a Wyatt ViscoStar-II viscometer. The column temperature was maintained at 40 °C and the flow rate was 1 mL min^–1^. Samples were dissolved in tetrahydrofuran at an approximate concentration of 10 mg mL^–1^ and filtered prior to analysis. Data was processed using ASTRA software using d*n*/d*c* values of 0.042 and 0.079 for PLA and PCL, respectively.

Density functional calculations were conducted using the PBE0 functional,^60−62^ and the cc-pVTZ basis set,^63,64^ utilizing the Gaussian09 software;^65^ orbitals were visualized utilizing GaussView5.^66^ Quantum theory of atoms in molecules (QTAIM) analysis was conducted utilizing the AIMAll software,^67^ and natural bonding orbitals (NBOs) were calculated using NBO version 3^68−75^ within Gaussian09.

#### Synthesis of H_2_L^2^

*N*-phenyl-*N*′-(2-pyridinyl)1,2-ethylene diamine (2.38 g, 9.86 mmol) was combined with 2,4-tert-butylphenol (4.06 g, 19.72 mmol) and formaldehyde (2.98 mL of a 37% w/w solution in water, 27.60 mmol) in methanol (20 mL), and stirred at reflux for 24 h. The reaction mixture was allowed to cool to room temperature. The precipitate formed was filtered under vacuum and washed with ice-cold methanol to give H_2_L^2^ as a colorless powder (3.33 mmol, 34%). ^1^H NMR (400 MHz, CDCl_3_): δ 10.67 (1H, br s, O*H*), 10.44 (1H, br s, O*H*), 8.56 (1H, m, ArNC*H*), 7.62–7.58 (1H, td, *J* = 7.68, 1.78 Hz, Ar*H*), 7.26–7.22 (5H, m, Ar*H*), 7.19 (d, *J* = 2.45 Hz, 1H, Ar*H*), 7.18–7.15 (m, 1H, Ar*H*), 7.14–7.12 (m, 2H, Ar*H*), 6.83–6.82 (1H, d, *J* = 2.32 Hz, Ar*H*), 6.79–6.78 (1H, d, *J* = 2.45 Hz, Ar*H*), 3.72 (4H, s, C*H*_2_), 3.69 (2H, s, C*H*_2_), 3.52 (2H, s, C*H*_2_), 2.83–2.69 (4H, m, C*H*_2_), 1.43 (s, 18H, C(C*H*_3_)_3_), 1.29 (s, 9H, C(C*H*_3_)_3_), 1.27 (s, 9H, C(C*H*_3_)_3_); ^13^C NMR (100 MHz, CDCl_3_): δ 157.5, 153.9, 153.8, 149.1, 140.6, Q140.6, 136.7, 136.6, 135.6, 129.5, 128.4, 127.4, 124.1, 123.7, 123.6, 123.1, 122.9, 122.3, 121.23, 121.1, 59.5 (N*C*H_2_), 59.2 (N*C*H_2_), 59.0 (N*C*H_2_), 57.5 (N*C*H_2_), 50.5 (N*C*H_2_), 49.5 (N*C*H_2_), 34.88 (*C*(CH_3_)_3_), 34.82 (*C*(CH_3_)_3_), 34.11 (*C*(CH_3_)_3_), 34.09 (*C*(CH_3_)_3_), 31.68 (C(*C*H_3_)_3_), 31.65 (C(*C*H_3_)_3_), 29.57 (C(*C*H_3_)_3_), 29.55 (C(*C*H_3_)_3_); ESI-MS *m*/*z* calc [C_45_H_63_N_3_O_2_H]^+^ 678.4993, found 678.4988.

#### Synthesis of **1**

To a vial was added La[N(SiHMe_2_)_2_]_3_(THF)_2_ (0.340 g, 0.5 mmol) and H_2_L^1^ (0.339 g, 0.5 mmol). Hexane (8 mL) was added and the reaction mixture was stirred at room temperature for 20 h, over which time a colorless precipitate formed. The precipitate was filtered under vacuum and washed with hexane, giving the product as a colorless powder (0.380 g, 0.39 mmol, 79%). It was possible to obtain further product by leaving the filtrate in a freezer (−35 °C). ^1^H NMR (400 MHz, C_6_D_6_): δ 7.56 (m, 2H, Ar*H*), 7.14–7.03 (m, 8H, Ar*H*), 6.92 (m, 3H, Ar*H*), 6.80 (m, 1H, Ar*H*), 5.38 and 5.28 (2m, 2H total (ratio 2:1), Si*H*), 4.29 (br s, 1H, C*H*_2_), 4.09 (m, 4H, OC*H*_2_), 3.92 (br s, 2H, C*H*_2_), 3.82 (d, 1H, *J* = 13.6 Hz, C*H*_2_), 3.72 (d, 1H, *J* = 13.6 Hz, C*H*_2_), 3.55 (d, 1H, *J* = 12.8 Hz, C*H*_2_), 3.24 (br s, 1H, C*H*_2_), 2.94 (m, 2H, C*H*_2_), 2.36 (br s, 1H, C*H*_2_), 2.18 (m, 2H, C*H*_2_), 1.73 (s, 6H, C(C*H*_3_)_3_), 1.63 (br s, 12H, C(C*H*_3_)_3_), 1.56 (m, 4H, C*H*_2_ (THF)), 1.35 (br s, 12H, C(C*H*_3_)_3_), 1.34 (s, 6H, C(C*H*_3_)_3_), 0.464, 0.462, and 0.41 (3d, 12H total *J* = 2.9 Hz (ratio 1:1:1), Si(C*H*_3_)_2_); ^13^C NMR (100 MHz, C_6_D_6_): δ 162.67 (Ar*C*-O), 162.13 (Ar*C*-O), 137.53 (Ar*C*), 137.45 (Ar*C*), 137.10 (Ar*C*), 136.99 (Ar*C*), 132.2 (Ar*C*H), 131.4 (Ar*C*H), 129.7 (Ar*C*H), 129.0 (Ar*C*H), 127.6 (Ar*C*H), 126.8 (Ar*C*H), 124.9 (Ar*C*H), 124.0 (Ar*C*), 123.8 (Ar*C*), 70.2 (O*C*H_2_), 60.4 (*C*H_2_), 35.9 (*C*(CH_3_)_3_), 34.6 (*C*(CH_3_)_3_), 34.5 (*C*(CH_3_)_3_), 32.5 (C(*C*H_3_)_3_), 32.3 (*C*(CH_3_)_3_), 30.9 (C(*C*H_3_)_3_), 30.7 (C(*C*H_3_)_3_), 26.0 (*C*H_2_, THF), 4.1 (Si(*C*H_3_)_2_), 3.9 (Si(*C*H_3_)_2_); 3.4 (Si(*C*H_3_)_2_); Anal. Calc. (C_55_H_87_LaN_3_O_3_Si_2_): C, 63.93; H, 8.49; N, 4.07 Found C, 63.76; H, 8.38; N, 4.53.

#### Synthesis of **2**

Complex **2** was prepared in a manner identical to complex **1** using La[N(SiHMe_2_)_2_]_3_(THF)_2_ (0.340 g, 0.5 mmol) and H_2_L^2^ (0.339 g, 0.5 mmol) in hexane (8 mL), giving the product as a colorless powder (0.294 g, 0.31 mmol, 62%). At 298 K, there was evidence in the ^1^H NMR spectrum of a major and minor species in a 4:1 ratio. Where possible, these species have been distinguished in the assignment with integration relative to the signals at 8.45 and 8.75 ppm (1H each), respectively. Signals not assignable to one species are integrated relative to the total of the signals at 8.45 and 8.75 ppm (1H total). ^1^H NMR (400 MHz, C_7_D_8_): δ Major: 8.45 (m, 2H, ArNC*H*), 7.55 (d, 1H, *J* = 2.6 Hz, Ar*H*), 7.50 (d, 1H, *J* = 2.6 Hz, Ar*H*), 6.85 (m, 1H, Ar*H*), 6.72 (m, 2H, Ar*H*), 6.43 (m, 1H, Ar*H*), 6.35 (m, 1H, Ar*H*), 5.26 (m, 2H, Si*H*), 1.83 (s, 9H, C(C*H*_3_)_3_), 1.55 (s, 9H, C(C*H*_3_)_3_), 1.50 (s, 9H, C(C*H*_3_)_3_), 1.37 (s, 9H, C(C*H*_3_)_3_); Minor: 8.75 (m, 2H, ArNC*H*), 7.52 (d, 1H, *J* = 2.6 Hz), 7.47 (d, 1H, *J* = 2.6 Hz), 6.79 (m, 1H, Ar*H*), 6.75 (m, 2H, Ar*H*), 6.43 (m, 1H, Ar*H*), 6.32 (m, 1H, Ar*H*), 5.35 (m, 2H, Si*H*), 1.77 (s, 9H, C(C*H*_3_)_3_), 1.64 (s, 9H, C(C*H*_3_)_3_), 1.42 (s, 9H, C(C*H*_3_)_3_), 1.34 (s, 9H, C(C*H*_3_)_3_), 0.48 (d, *J* = 2.9 Hz 6H, Si(C*H*_3_)_2_), 0.45 Si(C*H*_3_)_2_); Additional signals: 7.10–6.93 (m, 8H), 4.29 (d, 0.8H, *J* = 12.0 Hz, C*H*_2_), 4.19 (br d, 0.7H, *J* = 12.2 Hz, C*H*_2_), 4.06 (d, 0.3H, *J* = 14.9 Hz, C*H*_2_), 3.89 (d, 1H, *J* = 14.4 Hz, C*H*_2_), 3.72 (m, 0.6H, C*H*_2_), 3.60 (br d, 0.4H, C*H*_2_), 3.45 (d, 1.2H, *J* = 14.4 Hz, C*H*_2_), 3.25 (d, 1.8H, *J* = 12.6 Hz, C*H*_2_), 2.99 (br d, 0.8H, *J* = 13.7 Hz, C*H*_2_), 2.93 (d, 1H, *J* = 15.0 Hz, C*H*_2_), 2.84 (m, 1.3H, C*H*_2_); ^13^C NMR (100 MHz, C_7_D_8_): δ 162.7 (Ar*C*-O), 162.4 (Ar*C*-O), 158.5 (ArN*C*), 149.6 (ArN*C*H), 141.6, 139.4, 137.2, 136.9, 136.73, 136.67, 133.5, 131.5, 131.4, 126.7, 126.4, 124.1, 123.6, 123.3, 123.2, 122.6, 121.4, 65.0 (*C*H_2_), 64.1 (*C*H_2_), 60.3 (*C*H_2_), 52.4 (*C*H_2_), 35.71 (*C*(CH_3_)_3_), 35.68 (*C*(CH_3_)_3_), 35.6 (*C*(CH_3_)_3_), 34.33 (*C*(CH_3_)_3_), 34.31 (*C*(CH_3_)_3_), 34.24 (*C*(CH_3_)_3_), 34.22 (*C*(CH_3_)_3_), 32.4 (C(*C*H_3_)_3_), 32.3 (C(*C*H_3_)_3_), 32.2 (C(*C*H_3_)_3_), 32.1 (C(*C*H_3_)_3_), 30.49 (C(*C*H_3_)_3_), 30.46 (C(*C*H_3_)_3_), 30.44 (C(*C*H_3_)_3_), 30.3 (C(*C*H_3_)_3_), 3.96 (Si(*C*H_3_)_2_), 3.94 (Si(*C*H_3_)_2_), 3.87 (Si(*C*H_3_)_2_); Anal. Calc. (C_49_H_75_LaN_4_O_2_Si_2_): C, 62.13; H, 7.98; N, 5.91 Found C, 65.49; H, 7.82; N, 5.60.

#### Synthesis of **3**

To a vial was added La[N(SiHMe_2_)_2_]_3_(THF)_2_ (0.340 g, 0.5 mmol) and H_2_L^3^ (0.340 g, 0.5 mmol). Toluene (8 mL) was added and the reaction mixture was stirred at room temperature for 20 h, over which time a colorless precipitate formed. The precipitate was filtered under vacuum and washed with toluene, giving the product as a colorless powder (0.213 g, 0.23 mmol, 45%). It was possible to obtain further product by leaving the filtrate in a freezer (−35 °C). ^1^H NMR (400 MHz, C_6_D_6_): δ 9.38 (m, 2H, ArNC*H*), 7.51 (d, 2H, *J* = 2.6 Hz, Ar*H*), 6.90 (2H, td, *J* = 7.7, 1.7 Hz, Ar*H*), 6.85 (d, 2H, *J* = 2.6 Hz, Ar*H*), 6.69 (m, 2H, Ar*H*), 6.41 (d, 2H, *J* = 7.7 Hz, Ar*H*), 5.71 (m, 2H, Si*H*), 4.00 (d, 2H, *J* = 14.2 Hz, NC*H*_2_), 3.87 (d, 2H, *J* = 12.3 Hz, NC*H*_2_), 2.80 (br s, 2H, NC*H*_2_), 2.33 (d, 2H, *J* = 14.2 Hz, NC*H*_2_), 2.26 (d, 2H, *J* = 12.3 Hz, NC*H*_2_), 2.06 (br s, 2H, NC*H*_2_), 1.42 (s, 18H, C(C*H*_3_)_3_), 1.35 (s, 18H, C(C*H*_3_)_3_), 0.72 (d, 6H, *J* = 3.0 Hz, Si(C*H*_3_)_2_), 0.69 (d, 6H, *J* = 3.0 Hz, Si(C*H*_3_)_2_); ^13^C NMR (100 MHz, C_6_D_6_): δ 163.7 (Ar*C*-O), 159.27 (ArN*C*), 151.81 (ArN*C*H), 138.96 (Ar*C*H), 136.2 (Ar*C*), 135.90 (Ar*C*), 126.0 (Ar*C*), 125.73 (Ar*C*H), 124.5 (Ar*C*H), 123.7 (Ar*C*H), 123.2 (Ar*C*H), 63.75 (*C*H_2_), 35.78 (*C*(CH_3_)_3_), 34.51 (*C*(CH_3_)_3_), 32.7 (C(*C*H_3_)_3_), 30.8 (C(*C*H_3_)_3_), 4.9 (Si(*C*H_3_)_2_), 4.6 (Si(*C*H_3_)_2_). Anal. Calc. (C_48_H_74_LaN_5_O_2_Si_2_.C_7_H_8_): C, 63.50; H, 7.25; N, 6.73 Found C, 63.47; H, 7.25; N, 6.94. The single molecule of toluene per molecule of **3** (present in the unit cell of the solid-state structure) could not be removed even after extended periods of time under high vacuum. Single crystals suitable for X-ray diffraction experiments were grown from a toluene solution of **3** at −35°. The structure of **3** was found to contain a high level of disorder, particularly of the dimethylsilyl groups and efforts to model this were unsuccessful, leading to large thermal ellipsoids, a high R1, and high residual electron density. Additionally, a solvent mask was applied to account for 1.5 molecules of disordered toluene per unit cell.

#### General Procedure for Homopolymerizations

To a vial charged with a stir bar were added either *rac*-LA or ε-CL (2 mmol) and solvent (toluene or THF, 1.5 mL). Separately, a solution of initiator in solvent (toluene or THF, 0.5 mL) was prepared. The initiator solution was added to the stirred monomer mixture. At a known time, the reaction was quenched by removing from the glovebox and adding hexane. The volatiles were removed *in vacuo* to give the crude polymer. Purification was achieved by precipitation: a solution of the polymer in the minimum required volume of chloroform was added dropwise with stirring into methanol (50 mL). The colorless precipitate was isolated by filtration under reduced pressure and dried further in a vacuum oven at 70 °C overnight.

#### General Procedure for Copolymerization Reactions

The procedure was identical to that for homopolymerizations, except both *rac*-LA or (*S,S*)-LA (2 mmol) and ε-CL (2 mmol), were dissolved in solvent in the first step.

## Supporting Information Available

^1^H and ^13^C NMR spectra of all ligands, complexes and copolymers, GPC traces of all copolymers, MALDI-ToF mass spectra, NMR spectra relating to additional experiments, and figures and data relating to computational calculations (PDF)
