# Supplementary material for: Selective Transesterification to Control Copolymer Microstructure in the Ring-Opening Copolymerization of Lactide and ε-Caprolactone by Lanthanum Complexes
Source: Inorg Chem. 2023 Dec 21;63(1):280–93. doi: 10.1021/acs.inorgchem.3c03120 (PMC10777408; doi:10.1021/acs.inorgchem.3c03120)
Supplement: Supplementary file 1 — ic3c03120_si_001.pdf [file ic3c03120_si_001.pdf]

Electronic Supplementary Information

Selective Transesterification to Control Copolymer Microstructure in the Ring-Opening Copolymerization of Lactide and ε-Caprolactone by Lanthanum Complexes

Bette Beament[a], Daniel Britton[a], Thomas Malcomson[b#], Geoffrey R. Akien[a], Nathan R. Halcovitch[a], Michael P. Coogan[a], Rachel H. Platel[a*]

[a] Department of Chemistry, Lancaster University, Lancaster, LA1 4YB, United Kingdom.

[b] Department of Chemistry, University of Manchester, Oxford Road, Manchester, M13 9PL, United Kingdom.

1. NMR Spectra of Ligands ... 5
Figure S1: ^1H NMR Spectrum of H_2L^1 in $CDCl_3$ ... 5
Figure S2: ^{13}C NMR Spectrum of H_2L^1 in $CDCl_3$ ... 5
Figure S3: ^1H NMR Spectrum of H_2L^2 in $CDCl_3$ ... 6
Figure S4: ^{13}C NMR Spectrum of H_2L^2 in $CDCl_3$ ... 6
Figure S5: ^1H NMR Spectrum of H_2L^3 in $CDCl_3$ ... 7
Figure S6: ^{13}C NMR Spectrum of H_2L^3 in $CDCl_3$ ... 7
2. NMR Spectra of Complexes ... 8
Figure S7: ^1H NMR Spectrum of Complex **1** in C_6D_6 ... 8
Figure S8: ^{13}C NMR Spectrum of Complex **1** in C_6D_6 ... 8
Figure S9: ^1H NMR Spectrum of Complex 2 in C_6D_6 ... 9
Figure S10: ^{13}C NMR Spectrum of Complex **2** in C_6D_6 ... 9
Figure S11: ^1H NMR Spectrum of Complex 3 in C_6D_6 ... 10
Figure S12: ^{13}C NMR Spectrum of Complex **3** in C_6D_6 ... 10
Figure S13: Variable Temperature ^1H NMR Spectra of Complex **1** in benzene-d_6 ... 11
Figure S14: Variable Temperature ^1H NMR Spectra of Complex **2** in toluene-d_8 ... 12
Figure S15: ^{15}N – ^1H HMBC NMR Spectrum of Complex **2** in toluene-d_8 ... 13
3. Data from Homopolymerization Studies ... 13
Table S1: *Rac*-LA Polymerization by **1** – **3**[a] ... 13
Table S2: *ε*-CL Polymerization by **1** – **3**[a] ... 14
4. NMR Spectra of Copolymers ... 14
Figure S16: ^1H NMR Spectrum of polymer from Table 1, entry 1 ... 14
Figure S17: ^1H NMR Spectrum of polymer from Table 1, entry 2 ... 15
Figure S18: ^1H NMR Spectrum of polymer from Table 1, entry 3 ... 15
Figure S19: ^1H NMR Spectrum of polymer from Table 1, entry 4 ... 16
Figure S20: ^1H NMR Spectrum of polymer from Table 1, entry 5 ... 16
Figure S21: ^1H NMR Spectrum of polymer from Table 1, entry 6 ... 17
Figure S22: Carbonyl region of the ^{13}C NMR Spectrum (quantitative) of polymer from Table 1, entry 3 ... 17
Figure S23: Carbonyl region of the ^{13}C NMR Spectrum (quantitative) of polymer from Table 1, entry 5 ... 18
Figure S23: Carbonyl region of the ^{13}C NMR Spectrum (quantitative) of polymer from Table 1, entry 6 ... 18
Figure S24: ^1H NMR Spectrum of polymer from Table 1, entry 7 ... 19
Figure S25: ^1H NMR Spectrum of polymer from Table 1, entry 8 ... 19
Figure S25: ^1H NMR Spectrum of polymer from Table 1, entry 9 ... 20

Figure S26: ^{1}H NMR Spectrum of polymer from Table 1, entry 10 20

Figure S27: ^{1}H NMR Spectrum of polymer from Table 1, entry 11 21

Figure S28: ^{1}H NMR Spectrum of polymer from Table 1, entry 12 21

Figure S29: Carbonyl region of the ^{13}C NMR Spectrum (quantitative) of polymer from Table 1, entry 9 22

Figure S30: Carbonyl region of the ^{13}C NMR Spectrum (quantitative) of polymer from Table 1, entry 11 22

Figure S31: Carbonyl region of the ^{13}C NMR Spectrum (quantitative) of polymer from Table 1, entry 12 23

4. GPC Traces of Copolymers 23

Figure S32: GPC Trace of sample from Table 1 entry 1 23

Figure S33: GPC Trace of sample from Table 1 entry 2 24

Figure S34: GPC Trace of sample from Table 1 entry 3 24

Figure S35: GPC Trace of sample from Table 1 entry 4 25

Figure S36: GPC Trace of sample from Table 1 entry 5 25

Figure S37: GPC Trace of sample from Table 1 entry 6 26

Figure S38: GPC Trace of sample from Table 1 entry 7 26

Figure S39: GPC Trace of sample from Table 1 entry 8 27

Figure S40: GPC Trace of sample from Table 1 entry 9 27

Figure S41: GPC Trace of sample from Table 1 entry 10 28

Figure S42: GPC Trace of sample from Table 1 entry 11 28

Figure S43: GPC Trace of sample from Table 1 entry 12 29

5. X-ray data 29

Table S3**:** Single Crystal X-ray Data 29

6. MALDI-ToF Data 30

Figure S43: MALDI-ToF spectrum of a copolymer formed by reaction of complex **2** with LA and ε-CL with $[LA]_0$=[ε-CL]$_0$= 0.4 M and [**2**]:$[LA]_0$:[ε-CL]$_0$ = 1:20:20 in toluene. 30

Figure S44: MALDI-ToF spectrum of a copolymer formed by reaction of reaction of complex **3** with LA and ε-CL in the presence of 1 equiv ^{i}PrOH. $[LA]_0$=[ε-CL]$_0$= 0.25 M and [**3**]:$[LA]_0$:[ε-CL]$_0$ = 1:5:5 in toluene. 31

7. Additional Experiments 31

Scheme S1: Copolymerisation of active PLA and PCL chains promoted by complex **3** in THF. 31

Figure S45: ^{1}H NMR spectrum of the methine and methylene region ($CDCl_3$) of the copolymerisation of active PCL and PLA chains using complex **3** in THF. 32

Figure S46: Quantitative ^{13}C NMR spectrum of the carbonyl region ($CDCl_3$) of the copolymerisation of active PCL and PLA chains using complex **3** in THF. 32

Figure S47: ^{1}H NMR spectrum of the NMR scale reaction between **3** and 1 equiv (*S*)-ethyl-lactate in benzene-d_6. 33

Figure S48: (A) The carbonyl region of the ^{13}C NMR spectrum of the NMR scale reaction between **3** and 1 equiv (*S*)-ethyl-lactate in benzene-d_6 and (B) The carbonyl region of the ^{13}C NMR spectrum of (*S*)-ethyl-lactate in benzene-d_6 for comparison. 33

Figure S49: ^{1}H DOSY NMR spectrum of **1** 34

Figure S50: ^{1}H DOSY NMR spectrum of **2** 34

Figure S51: ^{1}H DOSY NMR spectrum of **3** 35

Figure S52: Showing ^{1}H NMR spectra from the NMR scale reaction of complex **1** with 1 equivalent $Ph_3P=O$ in C_6D_6. 37

Figure S53: Showing ^{1}H NMR spectra from the NMR scale reaction of complex **2** with 1 equivalent $Ph_3P=O$ in C_6D_6. 37

Figure S54: Showing ^{1}H NMR spectra from the NMR scale reaction of complex **3** with 1 equivalent $Ph_3P=O$ in C_6D_6. 38

Figure S55: Showing $^{31}P\{^{1}H\}$ NMR spectra from the NMR scale reaction of complexes **1**-**3** with 1 equivalent $Ph_3P=O$ in C_6D_6. 38

Table S4: Thermal characterisation data for selected copolymers. 39

Figure S56: ^{1}H NMR spectrum of a copolymer prepared by complex **2** in toluene* for DOSY analysis 39

Figure S57: ^{1}H DOSY NMR spectrum of a copolymer prepared by complex **2** in toluene 40

Figure S58: GPC trace of a copolymer prepared by complex **2** in toluene for DOSY analysis 40

Figure S59: ^{1}H NMR spectrum of a copolymer prepared by complex **3** in THF* for DOSY analysis 41

Figure S60: ^{1}H DOSY NMR spectrum of a copolymer prepared by complex **3** in THF 41

Figure S61: GPC trace of a copolymer prepared by complex **3** in THF for DOSY analysis 42

Figure S62: ^{1}H NMR spectrum of a copolymer prepared by complex **3** in toluene* for DOSY analysis 42

Figure S63: ^{1}H DOSY NMR spectrum of a copolymer prepared by complex **3** in toluene 43

Figure S64: GPC trace of a copolymer prepared by complex **3** in toluene for DOSY analysis 43

8. Calculations 44

Table S5: QTAIM metrics for complexes in their initial state (left) and when bound with ring-opened lactide (right). 44

Table S6: Percentage (%) difference in QTAIM metrics for complexes in their initial state (left) and when bound with ring-opened lactide (right), relative to complex **1**. 44

Figure S65: Natural bond orbitals representing interactions between the lanthanum ion and the ligand framework showing character from both the donating ligand atom and the lanthanum for complex **1** (top), **2** (middle), and **3** (bottom). 45

Figure S66: HOMO (left) and LUMO (right) molecular orbitals for complexes **1** (top), **2** (middle), and **3** (bottom). 45

1. NMR Spectra of Ligands

Figure S1: ^{1}H NMR Spectrum of H_2L^1 in $CDCl_3$

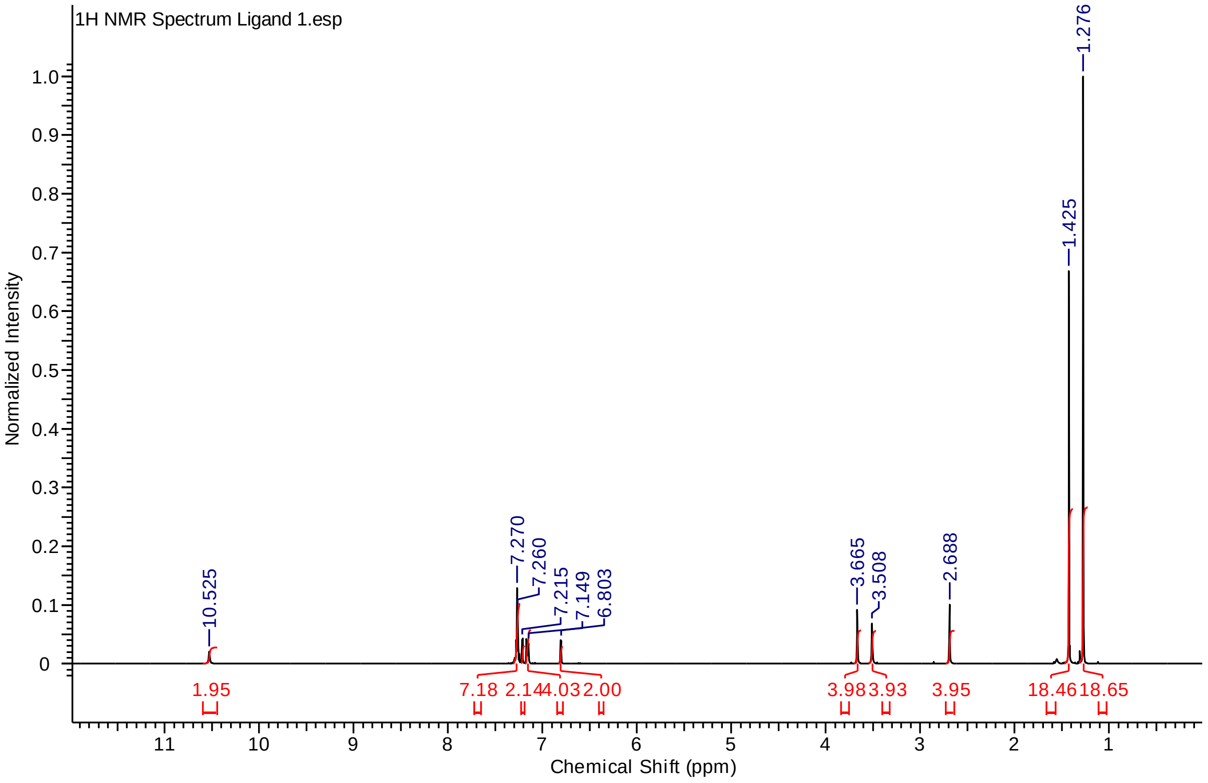

Figure S2: ^{13}C NMR Spectrum of H_2L^1 in $CDCl_3$

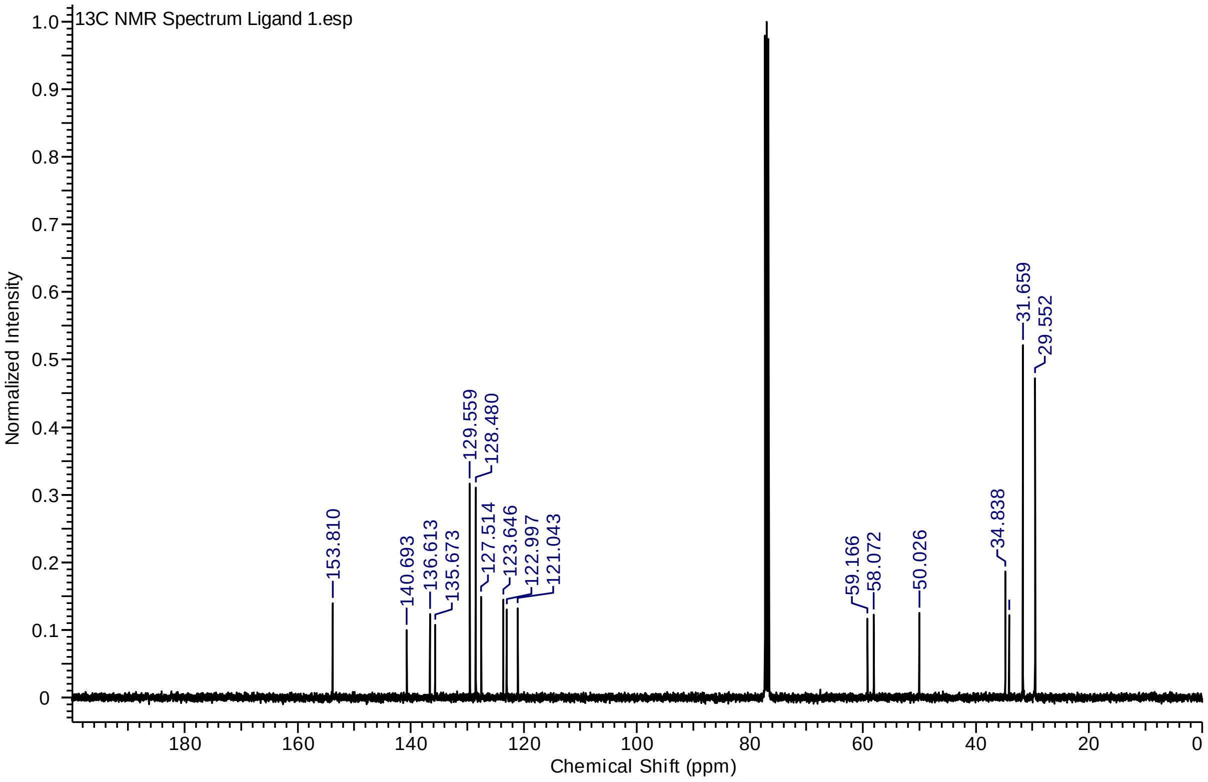

Figure S3: ^{1}H NMR Spectrum of H_2L^2 in $CDCl_3$

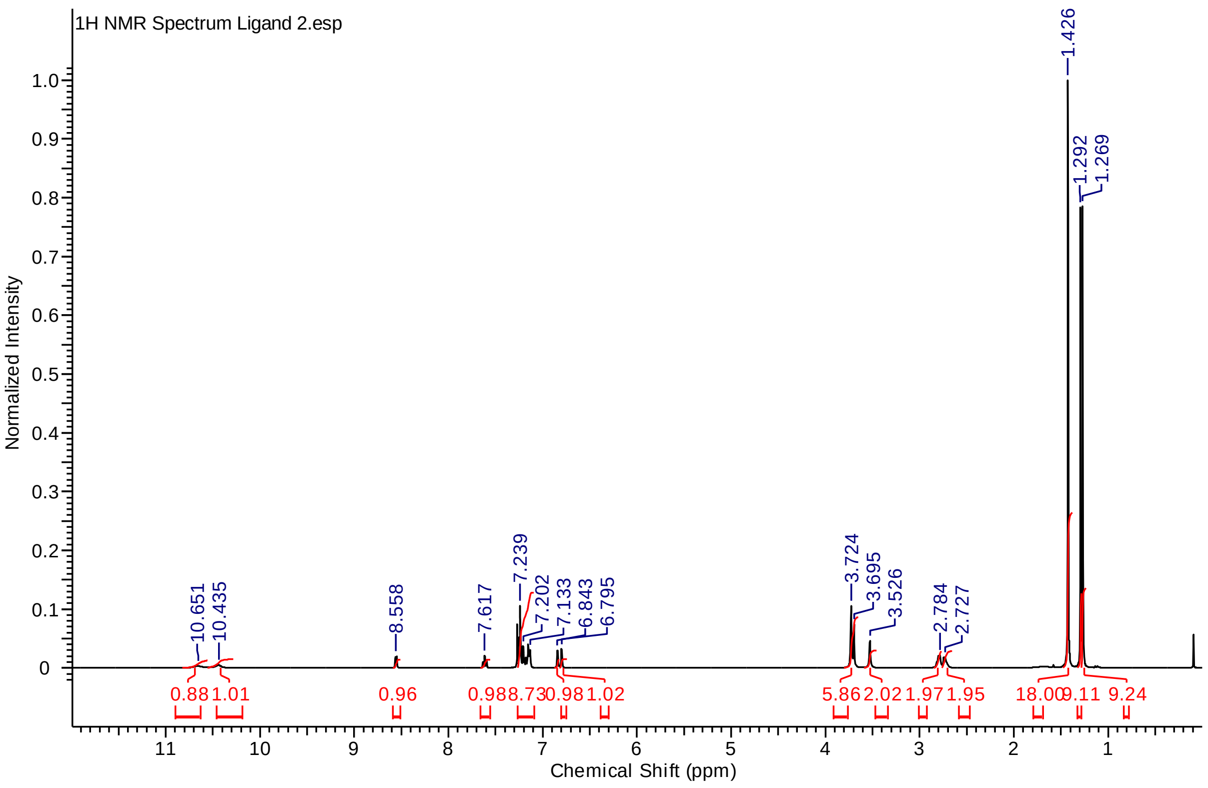

Figure S4: ^{13}C NMR Spectrum of H_2L^2 in $CDCl_3$

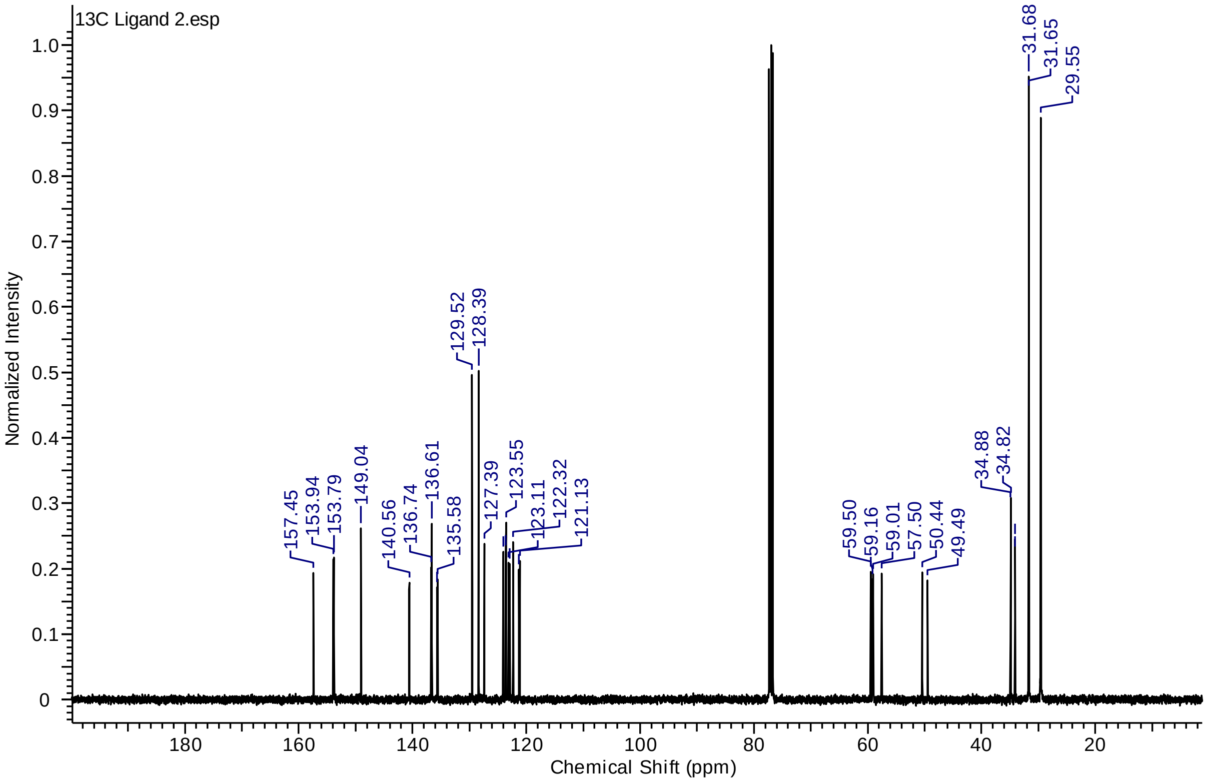

Figure S5: ^{1}H NMR Spectrum of H_2L^3 in $CDCl_3$

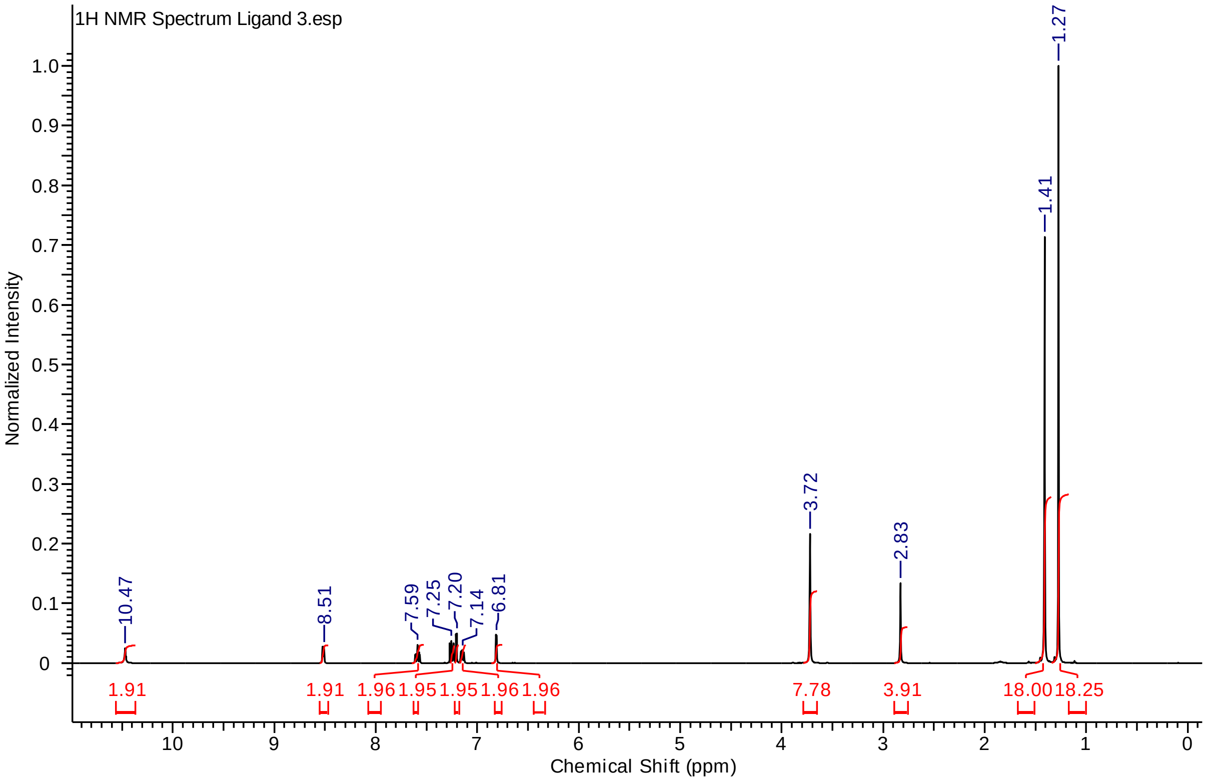

Figure S6: ^{13}C NMR Spectrum of H_2L^3 in $CDCl_3$

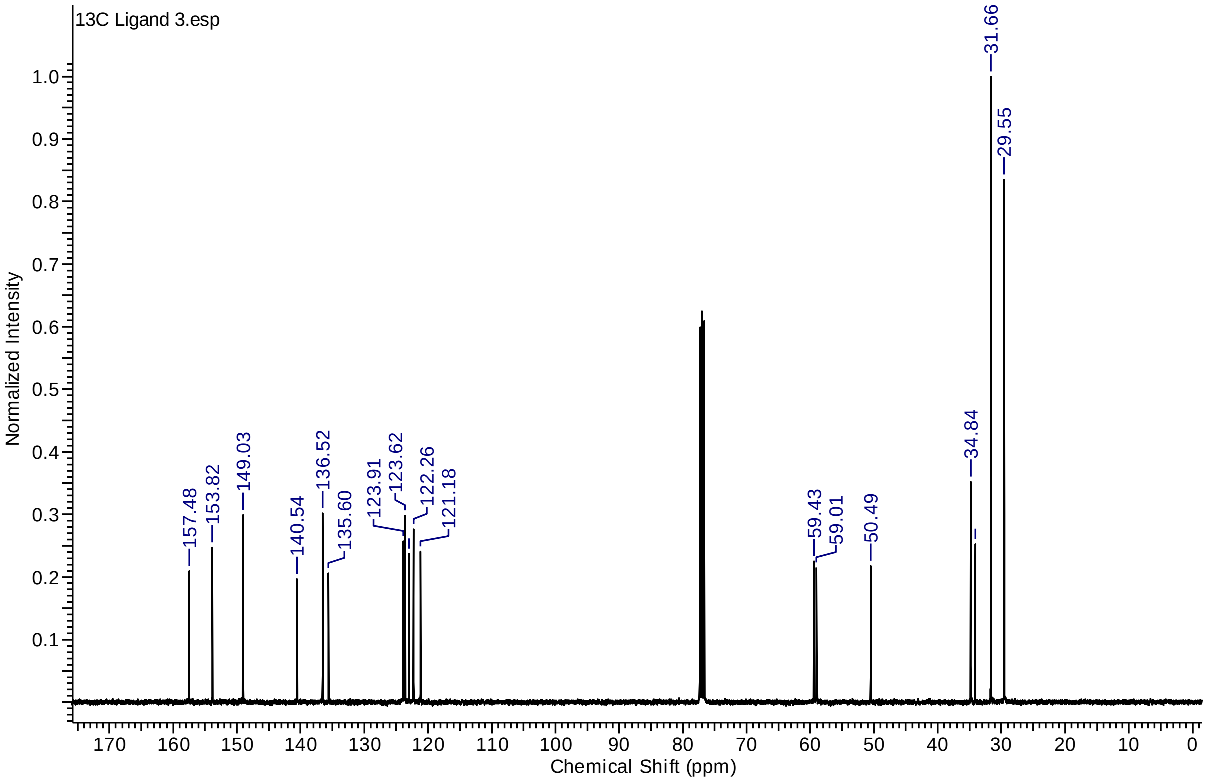

2. NMR Spectra of Complexes

Figure S7: ^{1}H NMR Spectrum of Complex **1** in C_6D_6

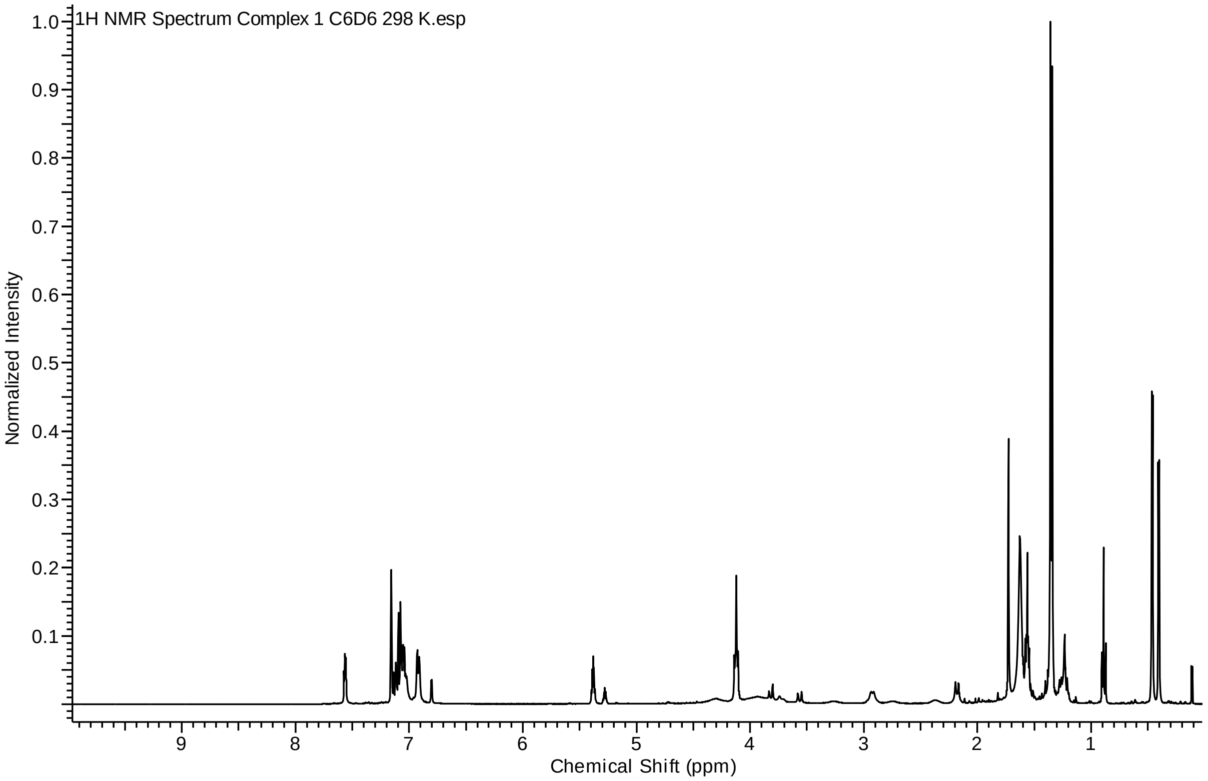

Figure S8: ^{13}C NMR Spectrum of Complex **1** in C_6D_6

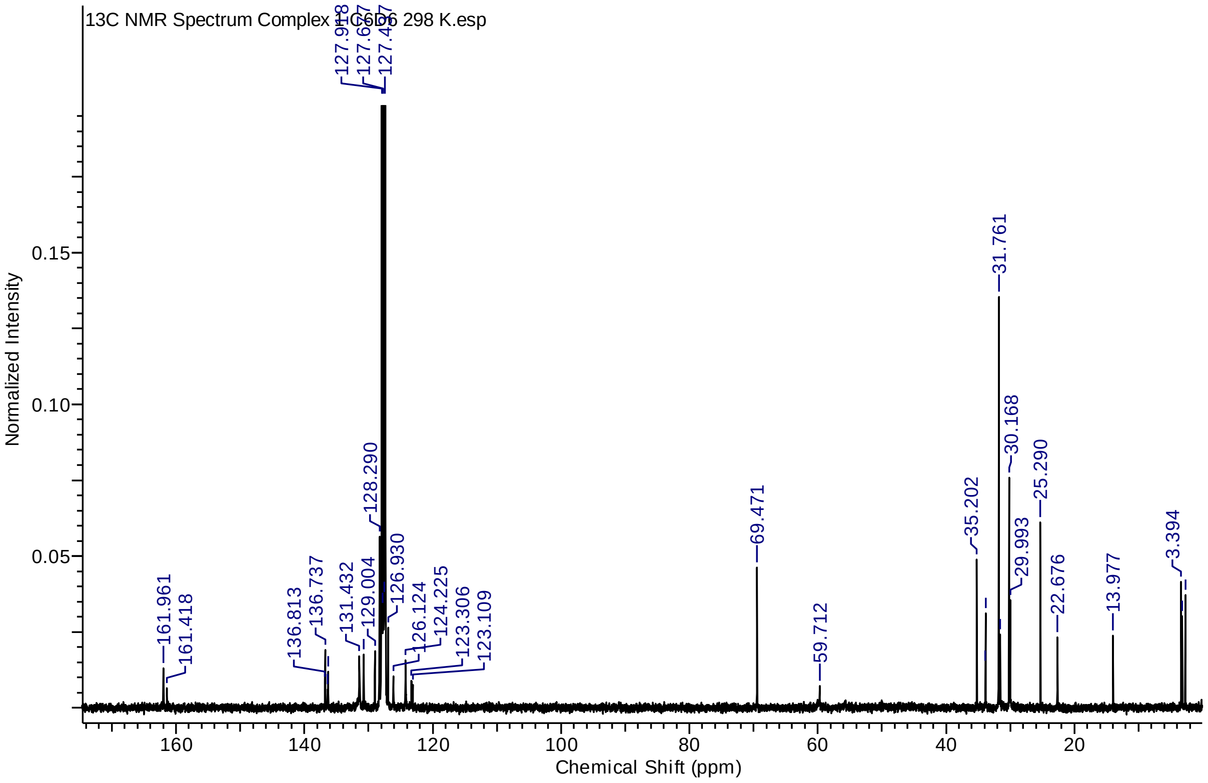

Figure S9: ^{1}H NMR Spectrum of Complex 2 in C_6D_6

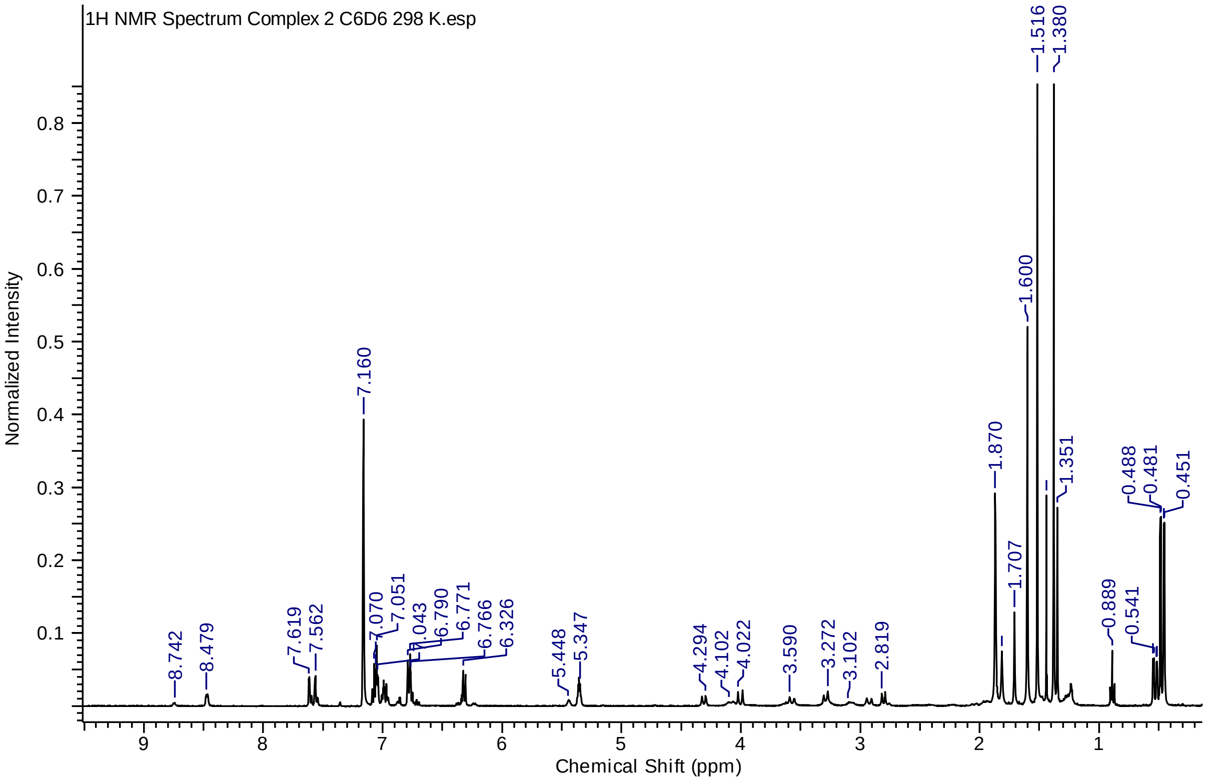

Figure S10: ^{13}C NMR Spectrum of Complex **2** in C_6D_6

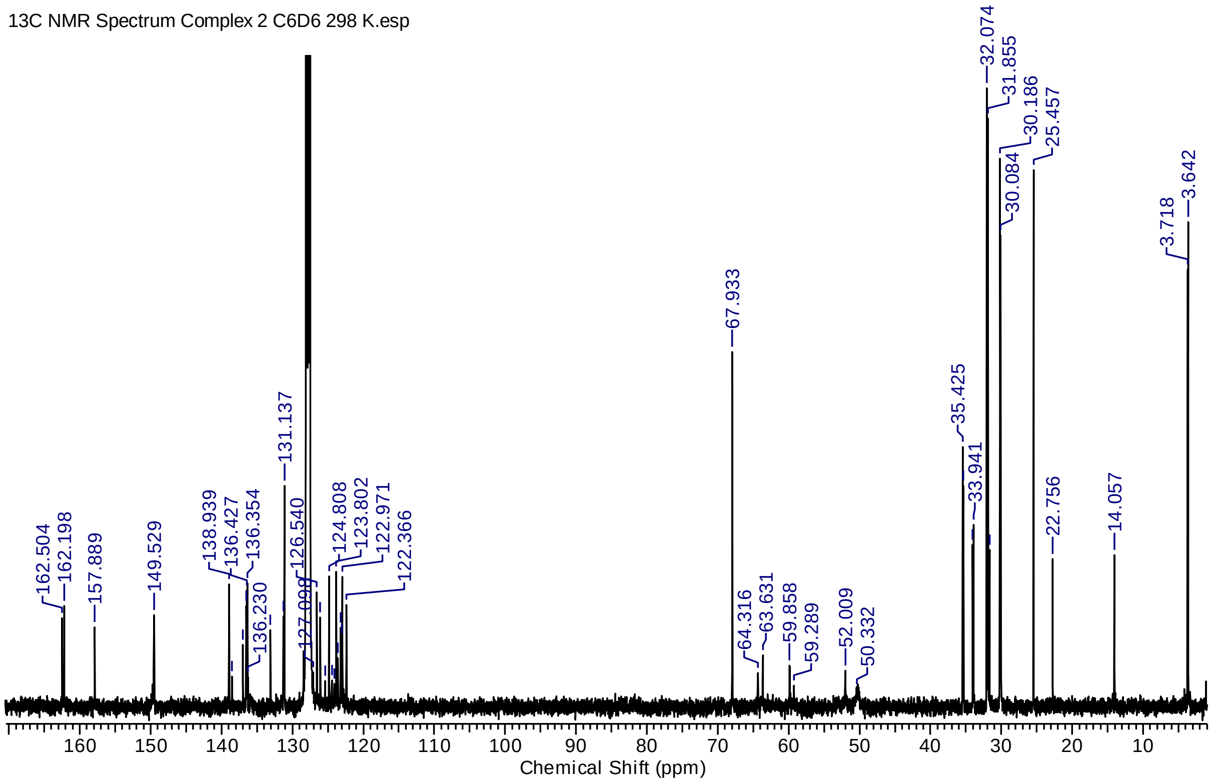

Figure S11: ^{1}H NMR Spectrum of Complex 3 in C_6D_6

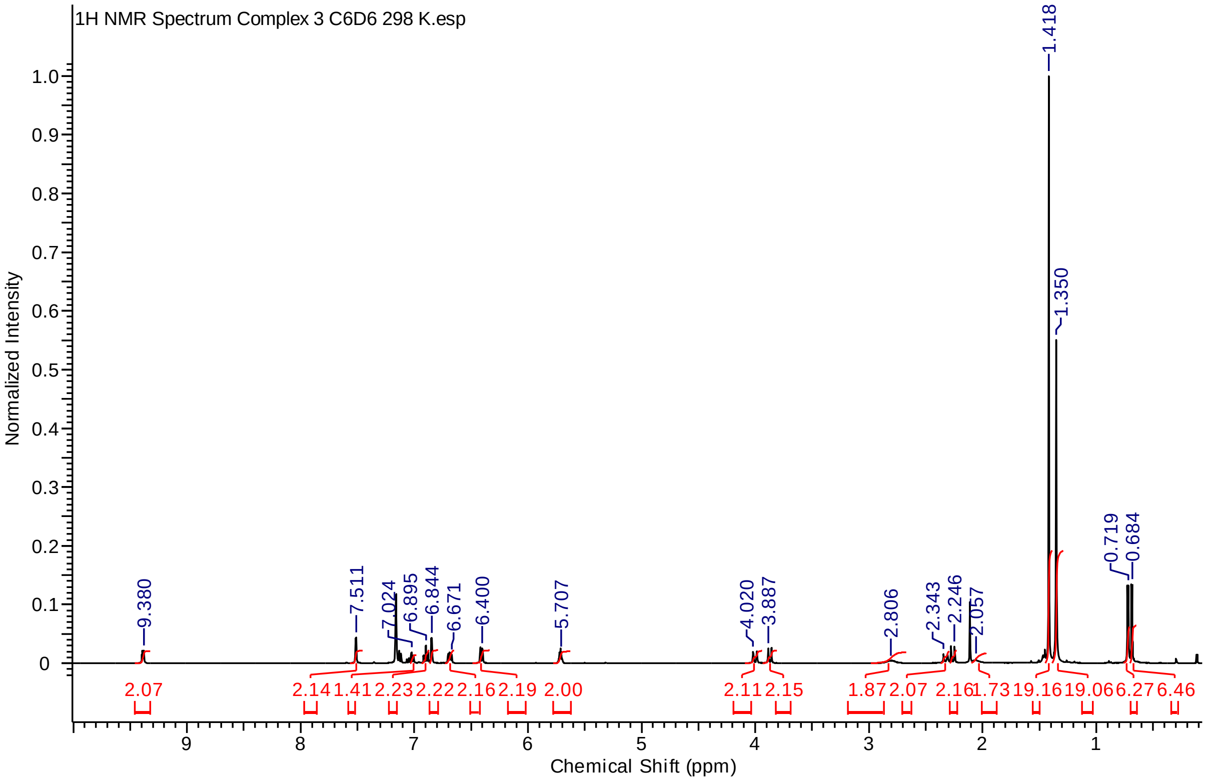

Figure S12: ^{13}C NMR Spectrum of Complex **3** in C_6D_6

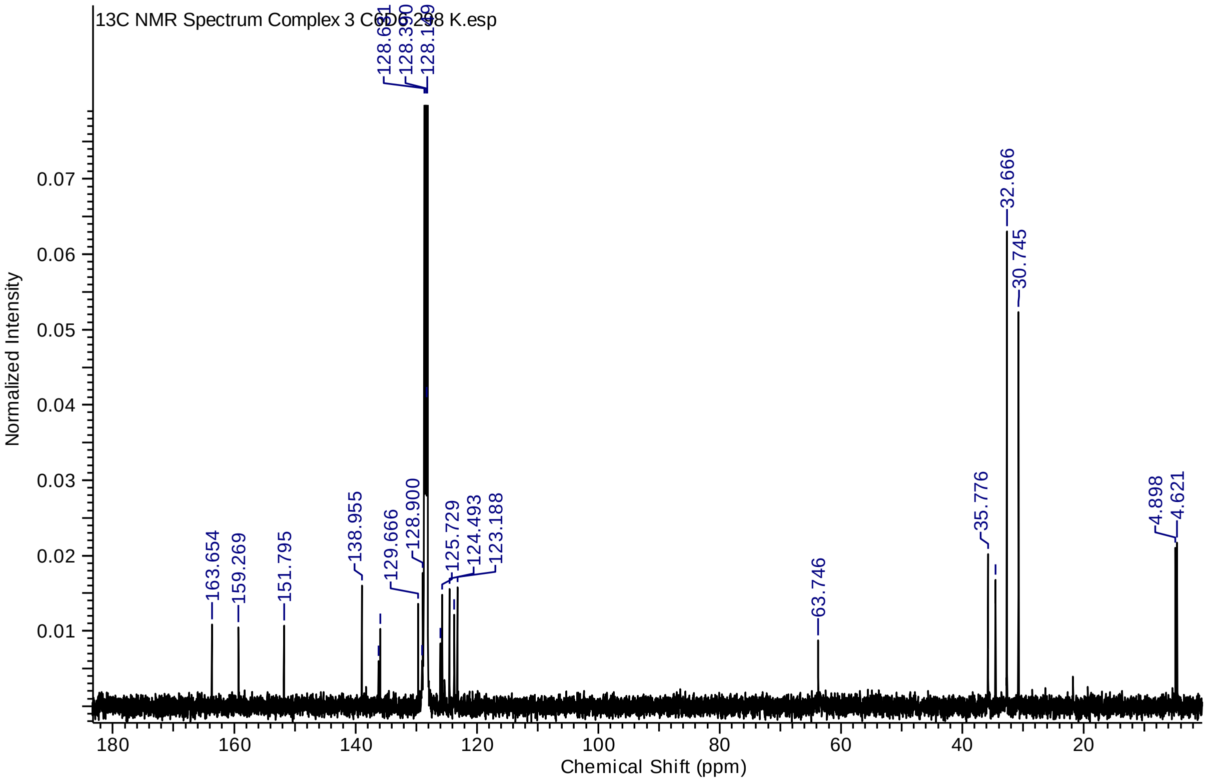

Figure S13: Variable Temperature ^{1}H NMR Spectra of Complex **1** in benzene-d_6

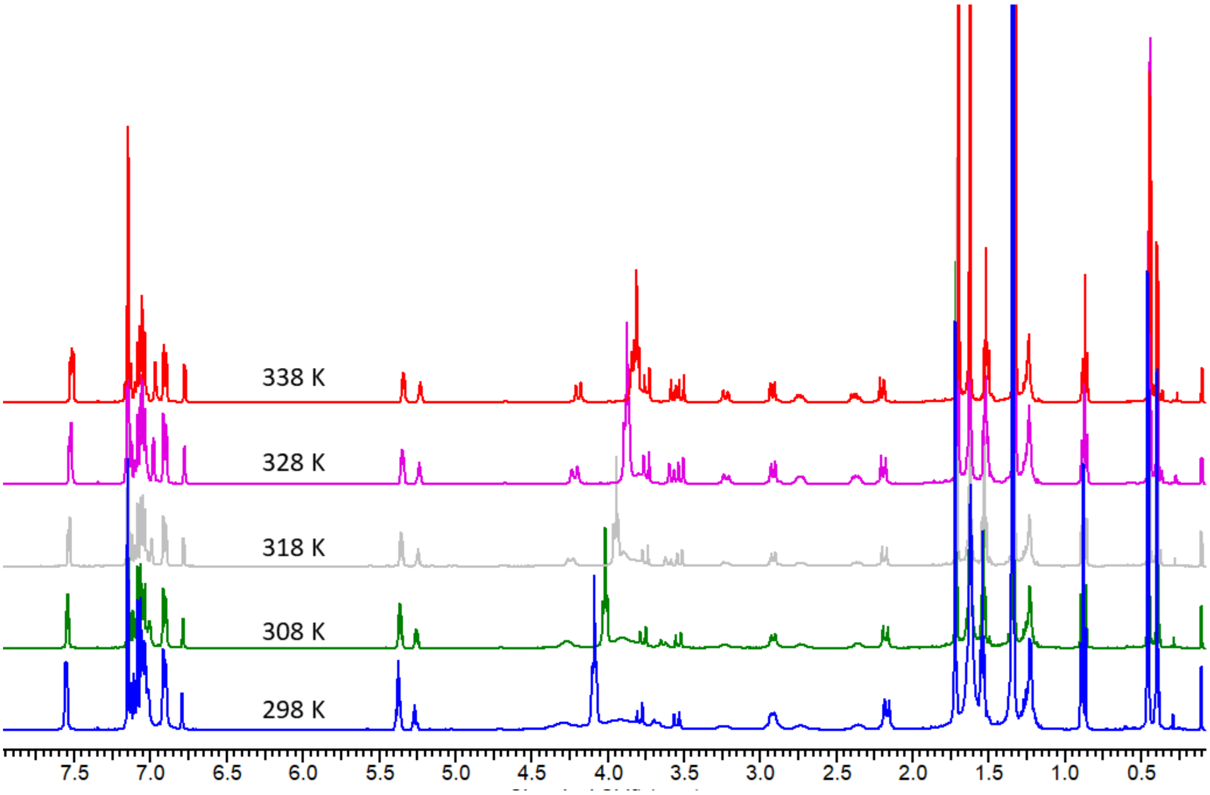

Figure S14: Variable Temperature ^{1}H NMR Spectra of Complex **2** in toluene-d_8

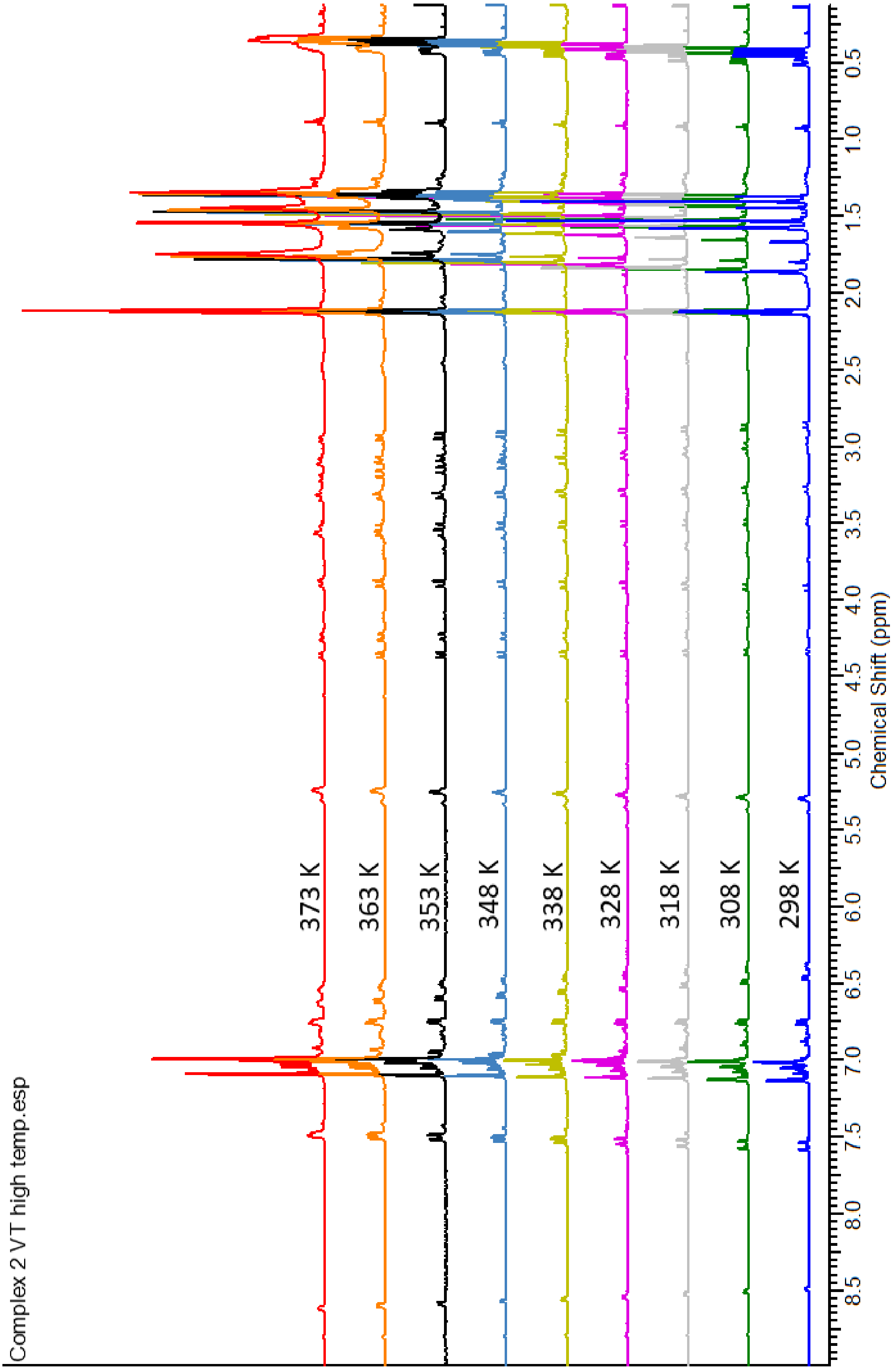

Figure S15: $^{15}N - ^{1}H$ HMBC NMR Spectrum of Complex **2** in toluene-d_8

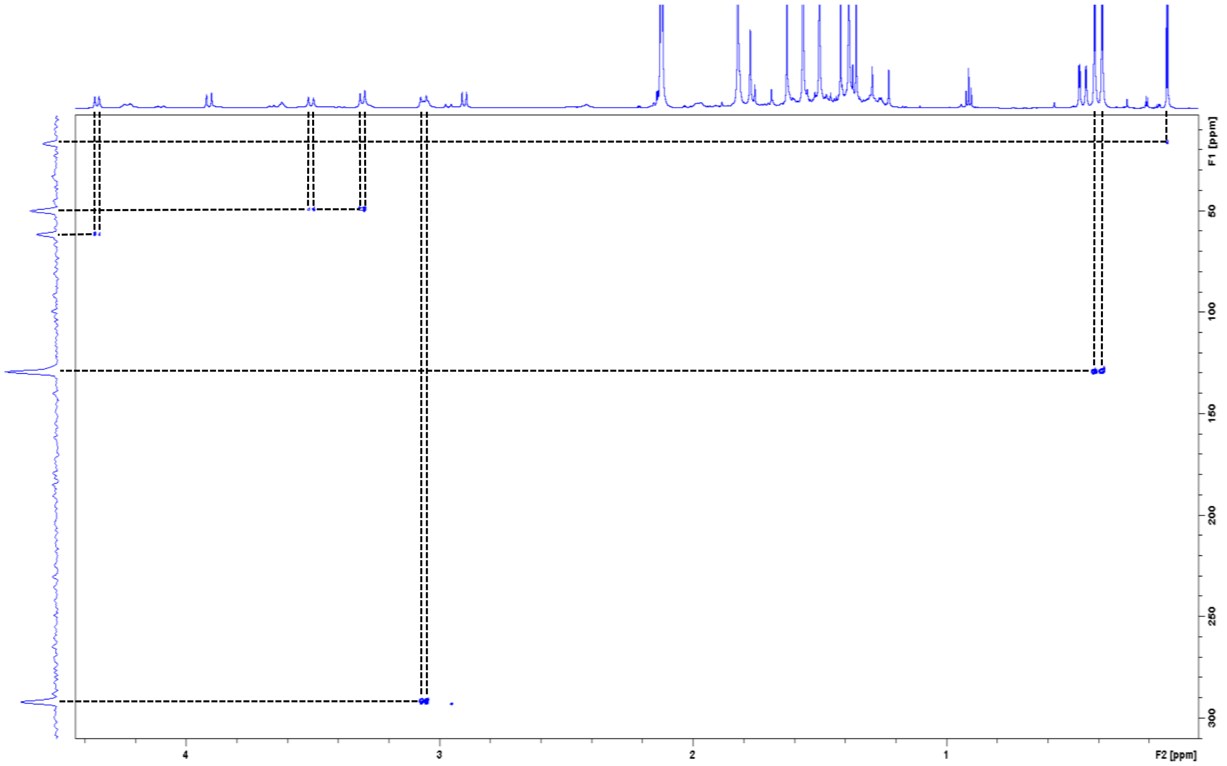

	328 K	328 K (55 °C)	
	Ligand H_2L^2 δ/ppm	Major δ/ppm	Minor δ/ppm
Pyridyl	315.6	292.0	293.2
Alkyl N (1)	44.5	49.5	nd[a]
Alkyl N (2)	41.1	62.7	nd[a]
SiN	-	129.0	129.0

nd = not detected

3. Data from Homopolymerization Studies

Table S1: *Rac*-LA Polymerization by **1** – **3**[a]

Complex	Monomer Conversion[b] /%	M_n x 10^3 /g mol^{-1} [c]	M_n(calc) x 10^3 /g mol^{-1}	$Đ$[c]
**1**	>99	46.2	28,800	1.28
**2**	>99	43.6	28,800	1.31
**3**	>99	183.2	28,800	1.17

[a]General polymerisation conditions: $[LA]_0$ = 1.0M in THF, $[LA]_0/[La]$ = 200, rt, 2 min. [b] Determined by ^1H NMR spectroscopy at 400 MHz in $CDCl_3$ [c] Number-average molecular weight (M_n) and dispersity ($Đ = M_w/M_n$) determined by GPC-MALLS at 40 °C in THF using a dn/dc value of 0.042 for PLA. [d]M_n(calc) = (144 x 200 x (% conversion LA/100)).

Table S2: ε-CL Polymerization by **1 – 3**[a]

Complex	Solvent	Monomer Conversion[b] /%	M_n x 10^3 /g mol^{-1} [c]	M_n(calc) x 10^3 /g mol^{-1}	$Đ$[c]
**1**	THF	>99	84.3	22,800	1.30
**2**	THF	>99	74.3	22,800	1.36
**3**	THF	>99	146.4	22,800	1.24
**1**	Toluene	>99	80.5[d]	22,800	1.32
**2**	Toluene	>99	107.4[d]	22,800	1.41
**3**	Toluene	>99	72.6[d]	22,800	1.33

[a]General polymerisation conditions: [ε-CL]$_0$ = 1.0M, [ε-CL]$_0$/[La] = 200, rt. [b] Determined by ^{1}H NMR spectroscopy at 400 MHz in $CDCl_3$ [c] Number-average molecular weight (M_n) and dispersity ($Đ = M_w/M_n$) determined by GPC-MALLS at 40 °C in THF using a dn/dc value of 0.079 for PCL. [d]M_n(calc) = (114 x 200 x (% conversion ε-CL/100)). [d] Bimodal distribution.

4. NMR Spectra of Copolymers

Figure S16: ^{1}H NMR Spectrum of polymer from Table 1, entry 1

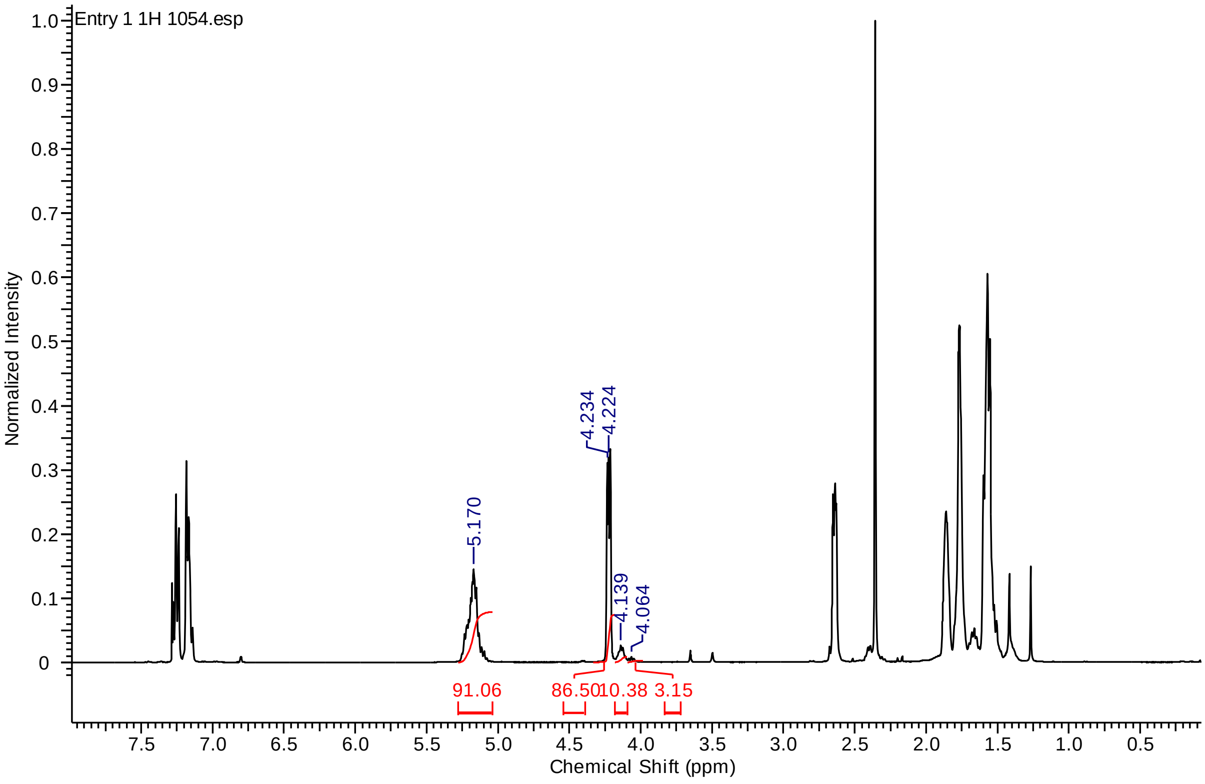

Figure S17: ^{1}H NMR Spectrum of polymer from Table 1, entry 2

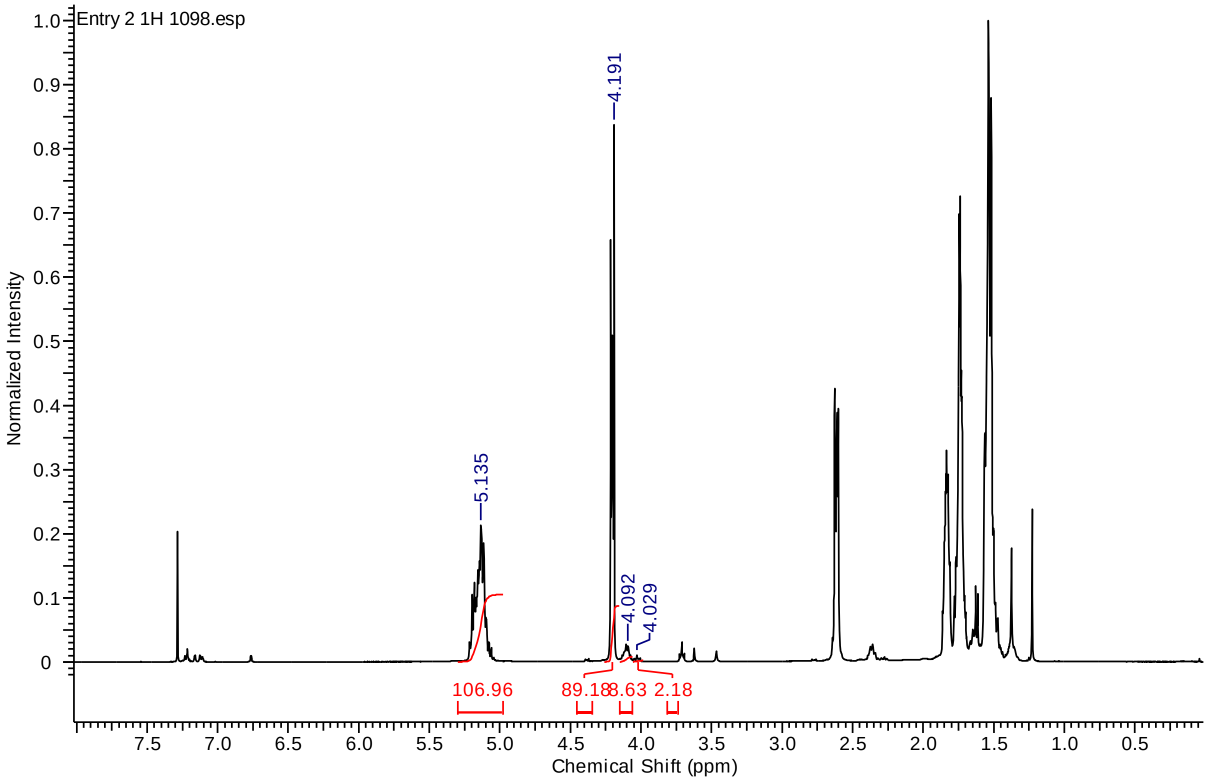

Figure S18: ^{1}H NMR Spectrum of polymer from Table 1, entry 3

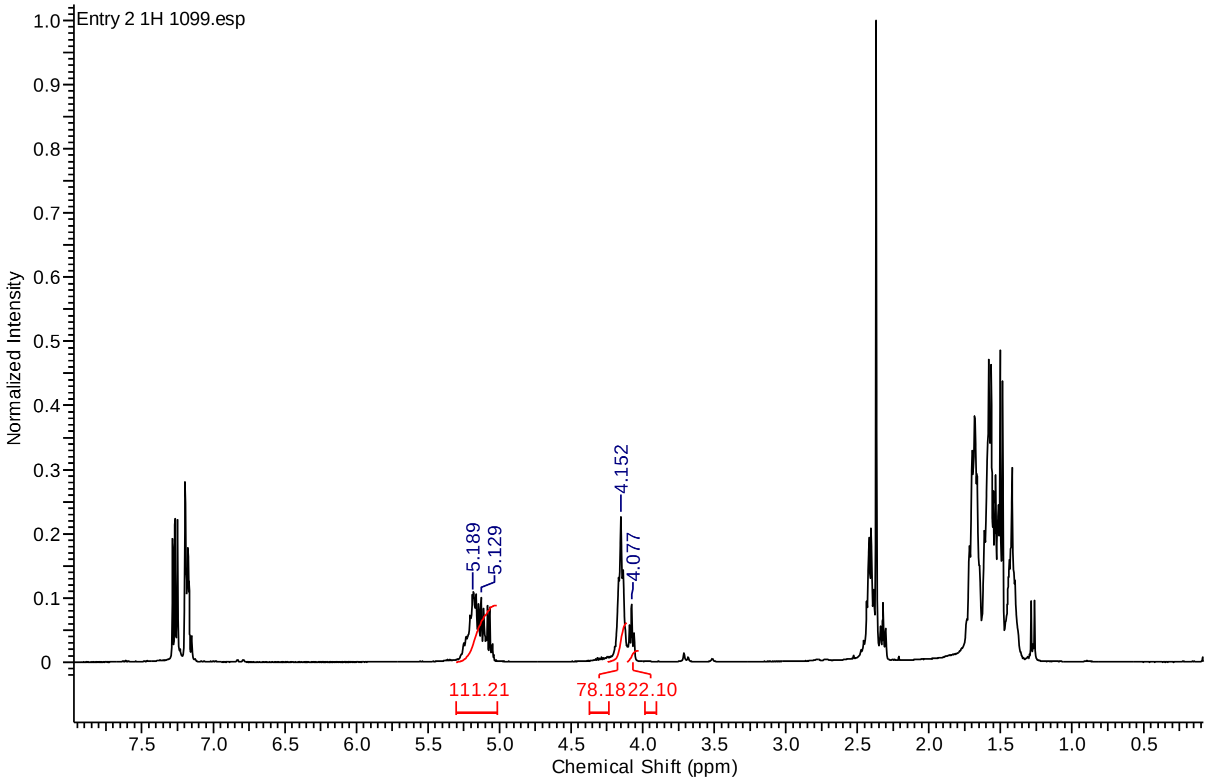

Figure S19: ^{1}H NMR Spectrum of polymer from Table 1, entry 4

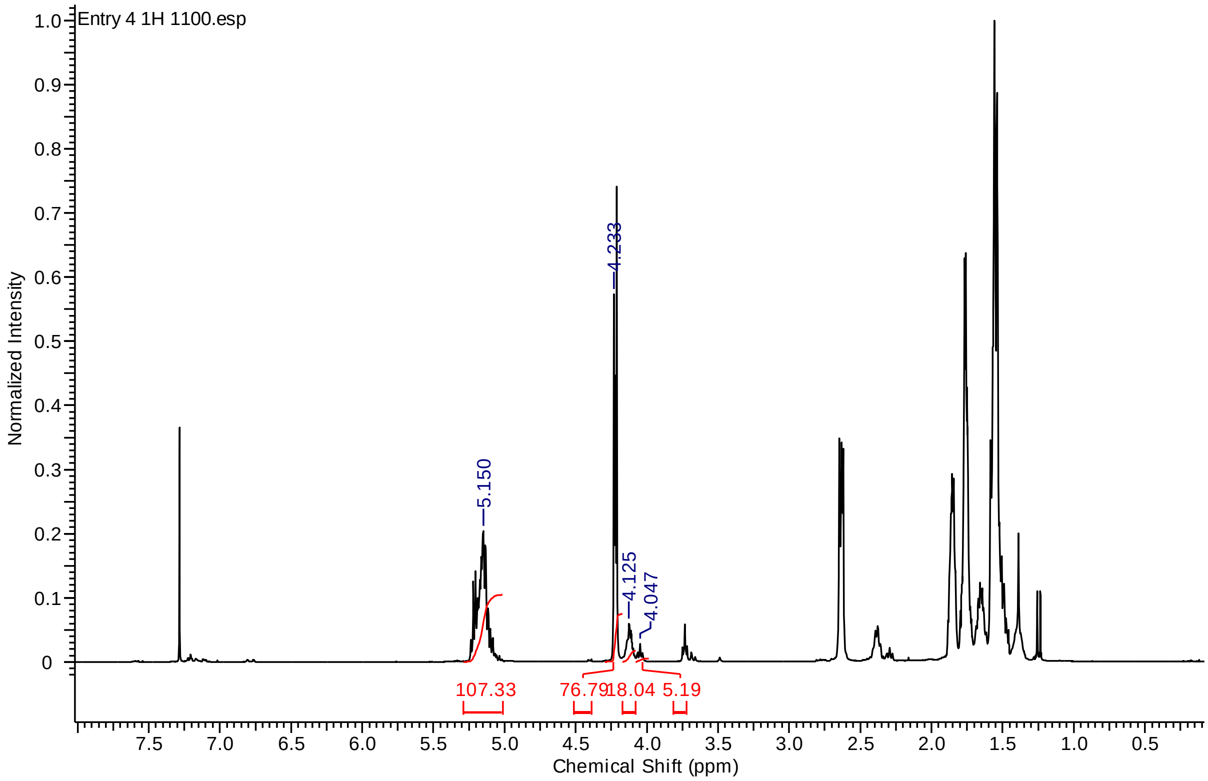

Figure S20: ^{1}H NMR Spectrum of polymer from Table 1, entry 5

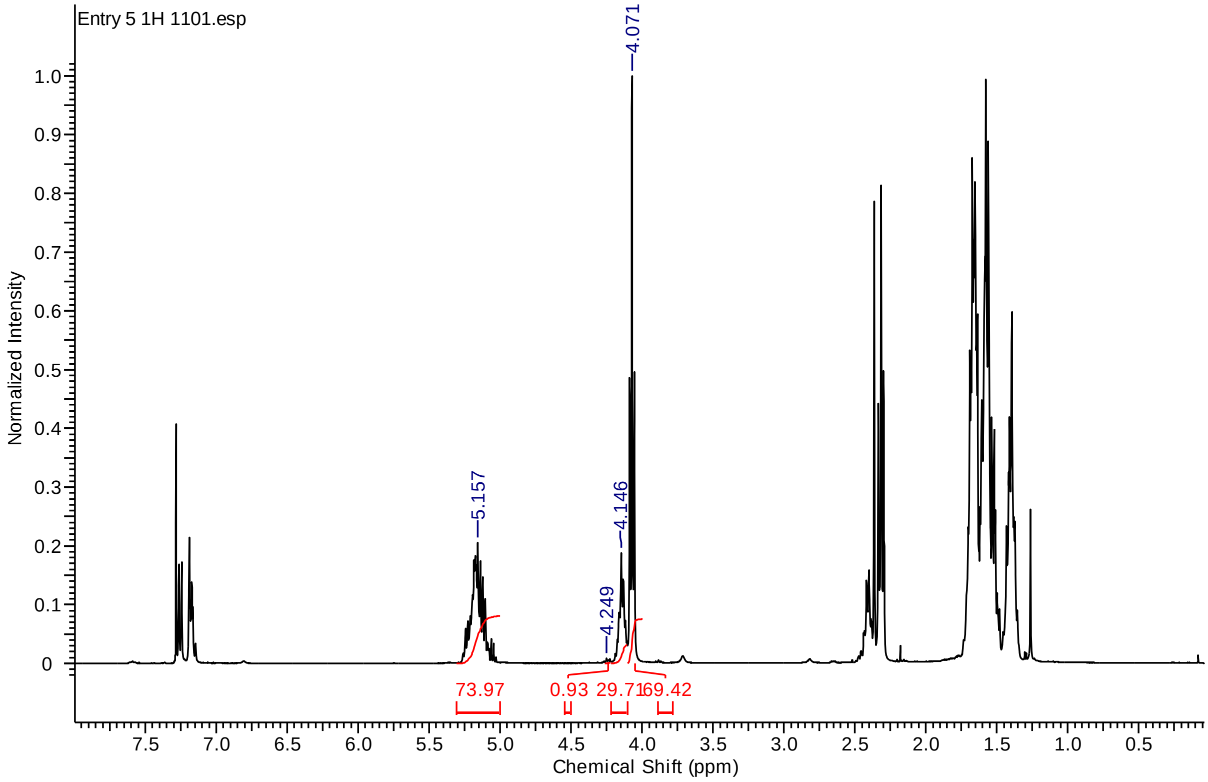

Figure S21: ^{1}H NMR Spectrum of polymer from Table 1, entry 6

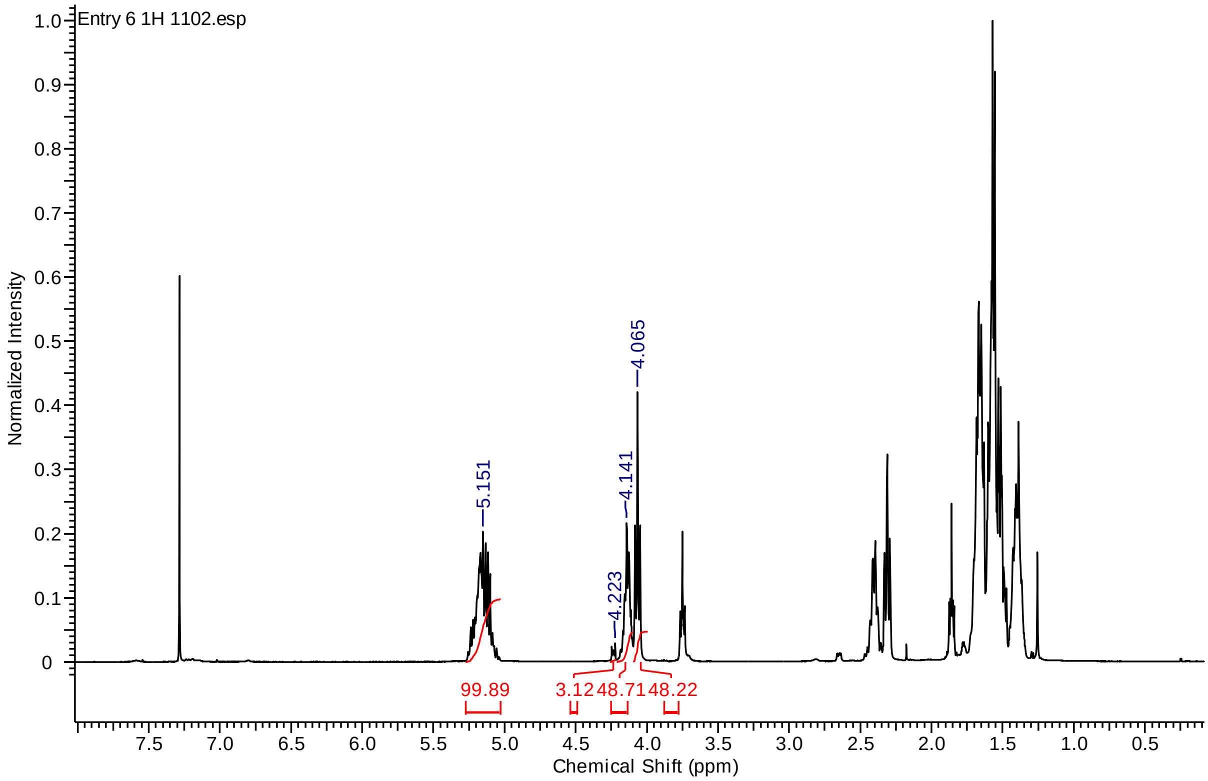

Figure S22: Carbonyl region of the ^{13}C NMR Spectrum (quantitative) of polymer from Table 1, entry 3

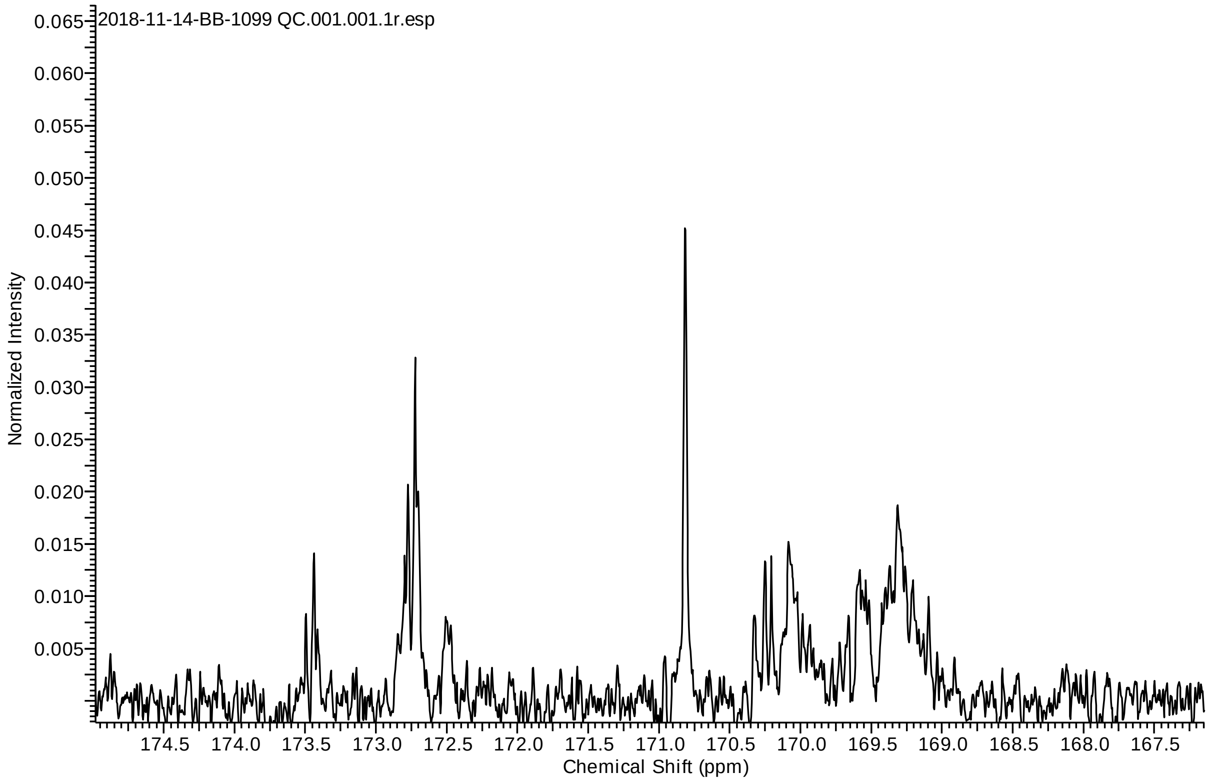

Figure S23: Carbonyl region of the ^{13}C NMR Spectrum (quantitative) of polymer from Table 1, entry 5

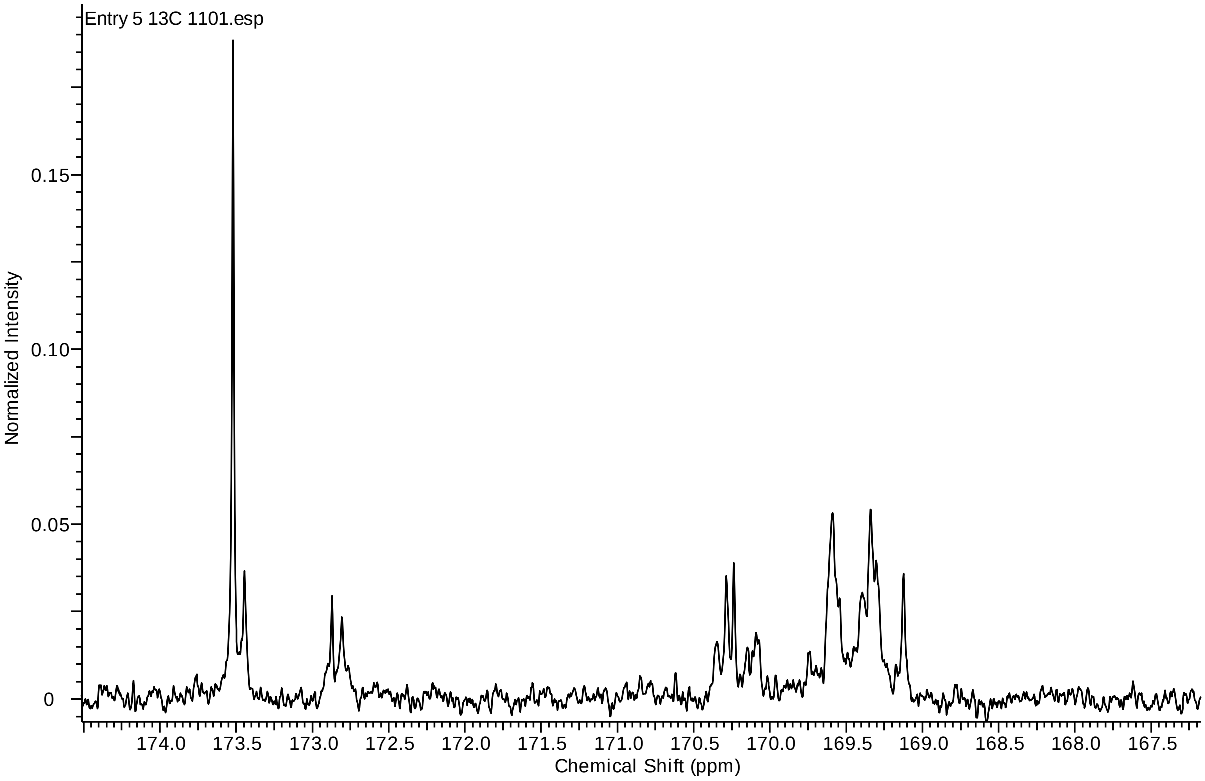

Figure S23: Carbonyl region of the ^{13}C NMR Spectrum (quantitative) of polymer from Table 1, entry 6

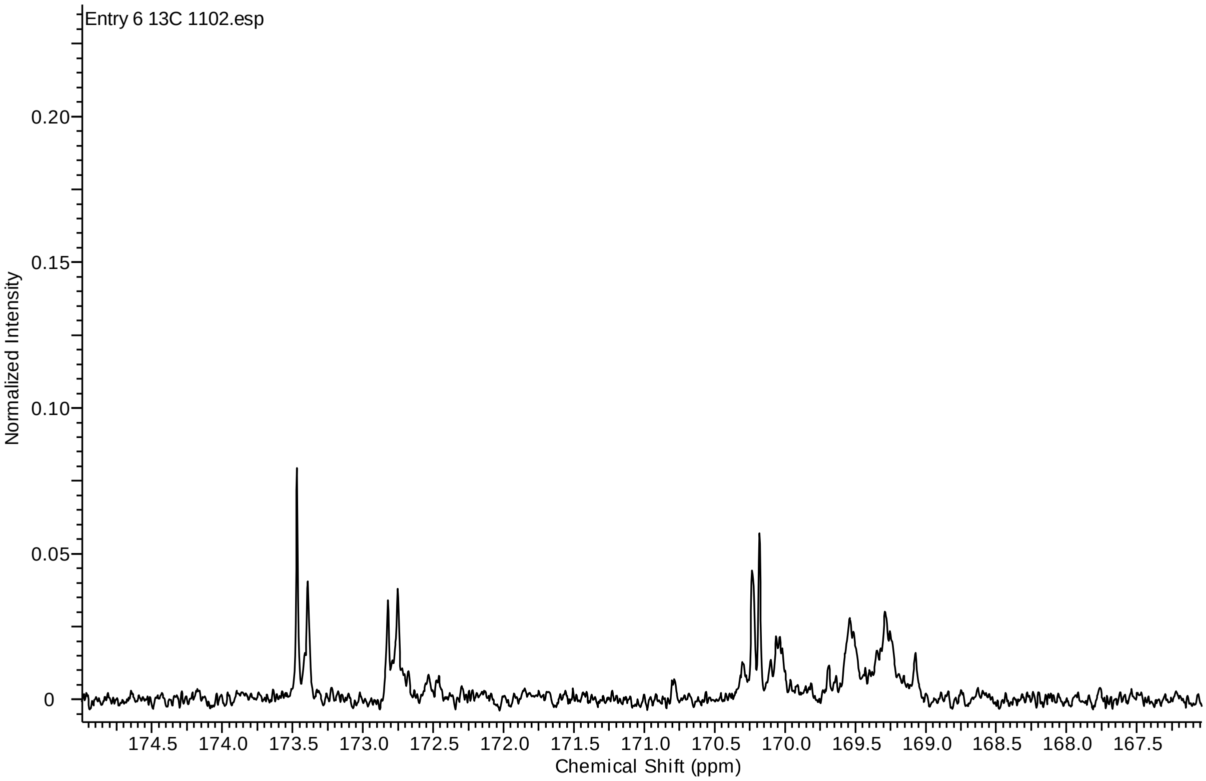

Figure S24: ^{1}H NMR Spectrum of polymer from Table 1, entry 7

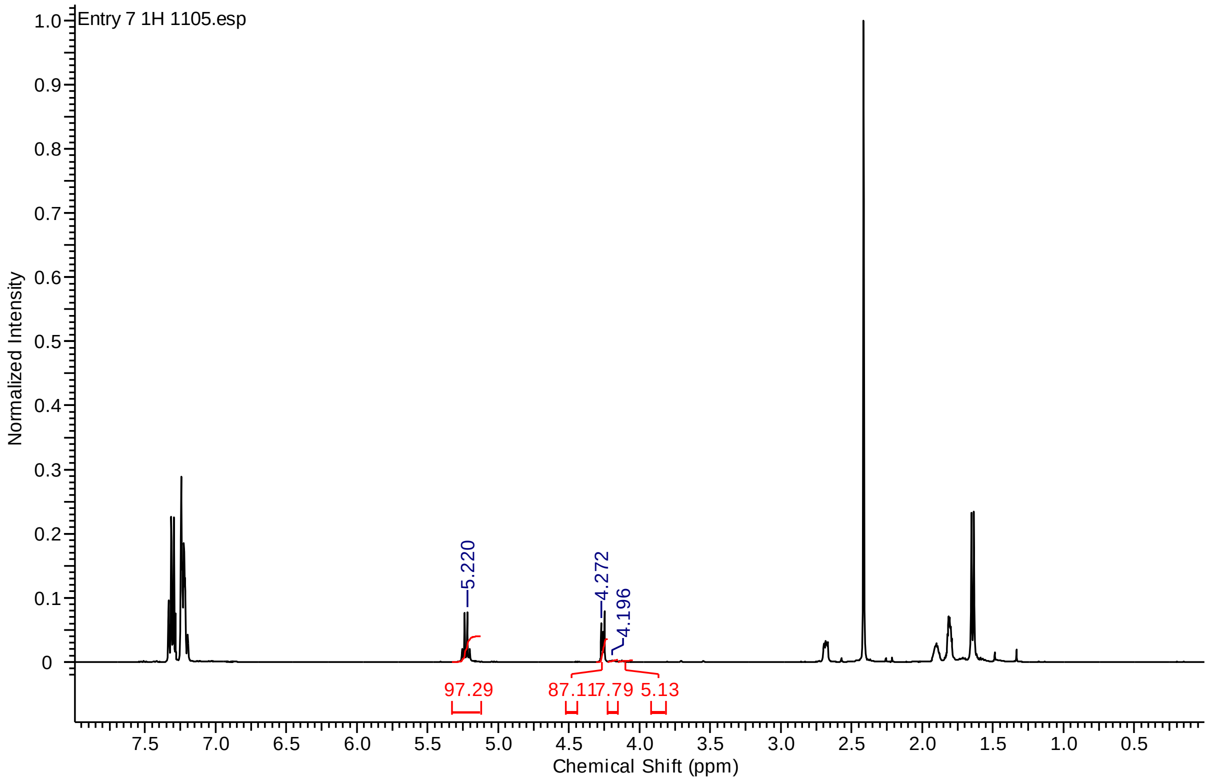

Figure S25: ^{1}H NMR Spectrum of polymer from Table 1, entry 8

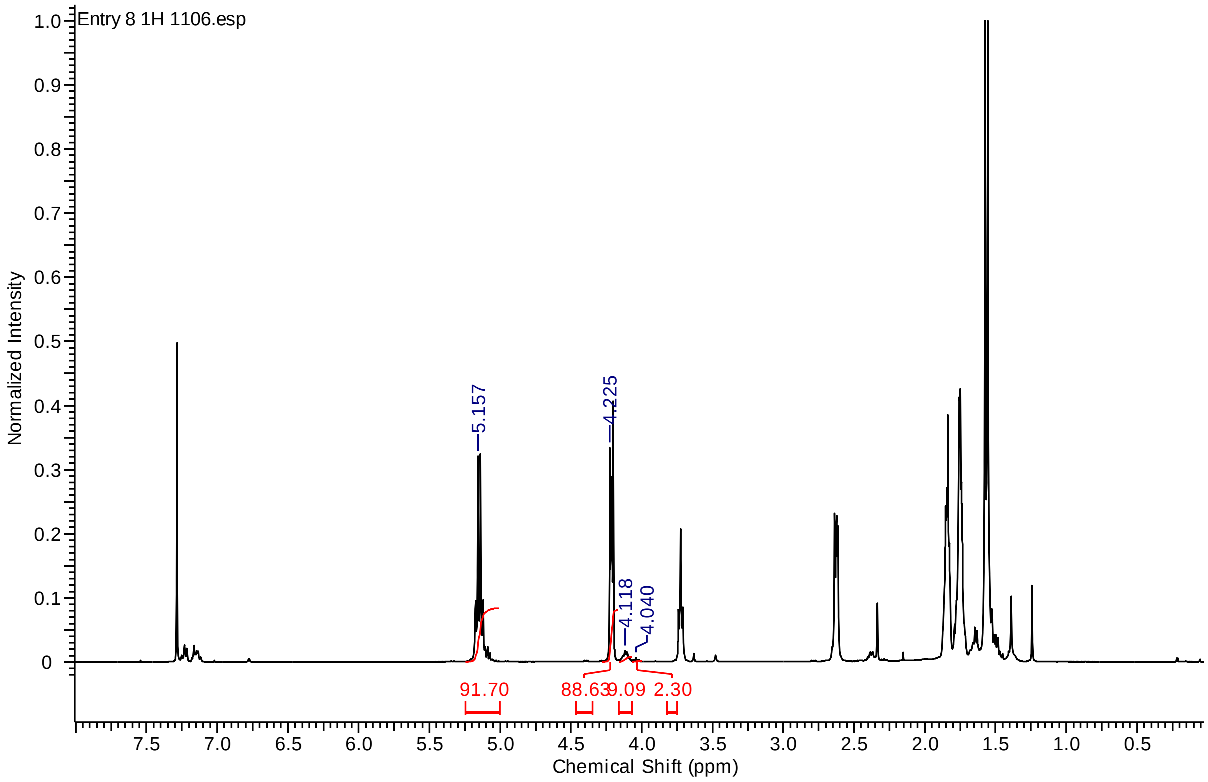

Figure S25: ^{1}H NMR Spectrum of polymer from Table 1, entry 9

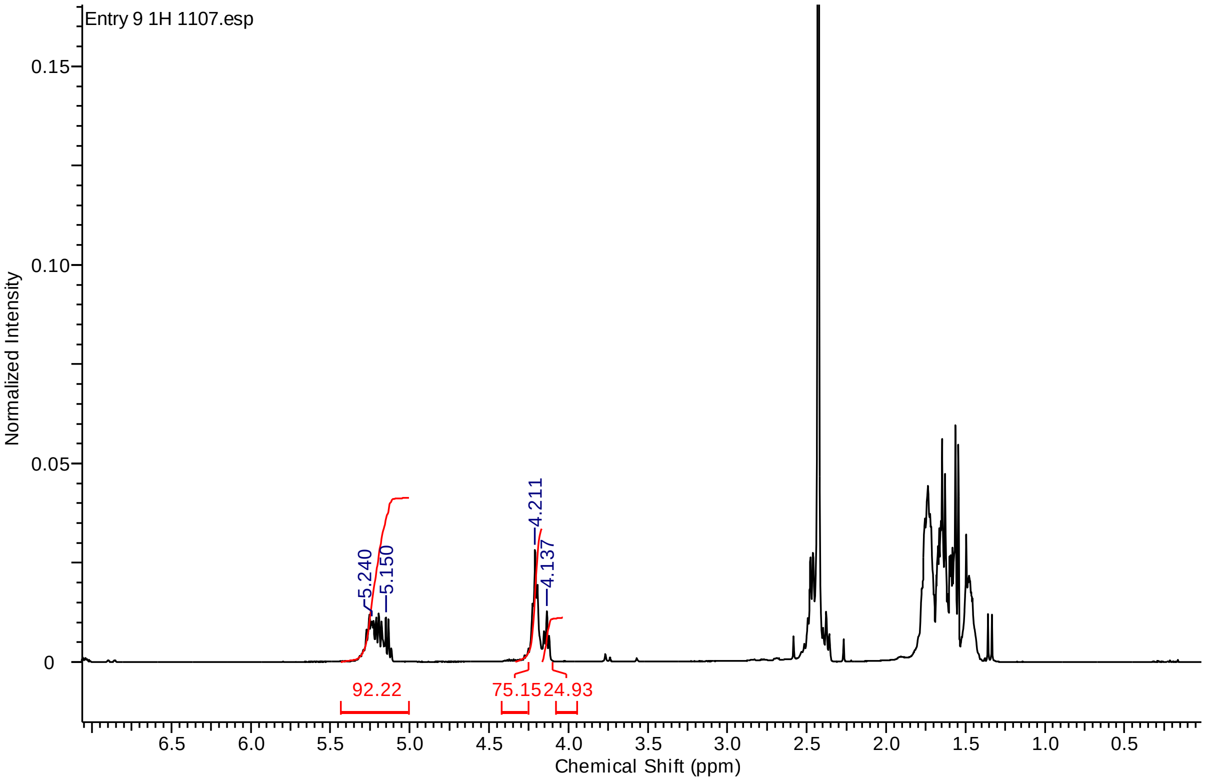

Figure S26: ^{1}H NMR Spectrum of polymer from Table 1, entry 10

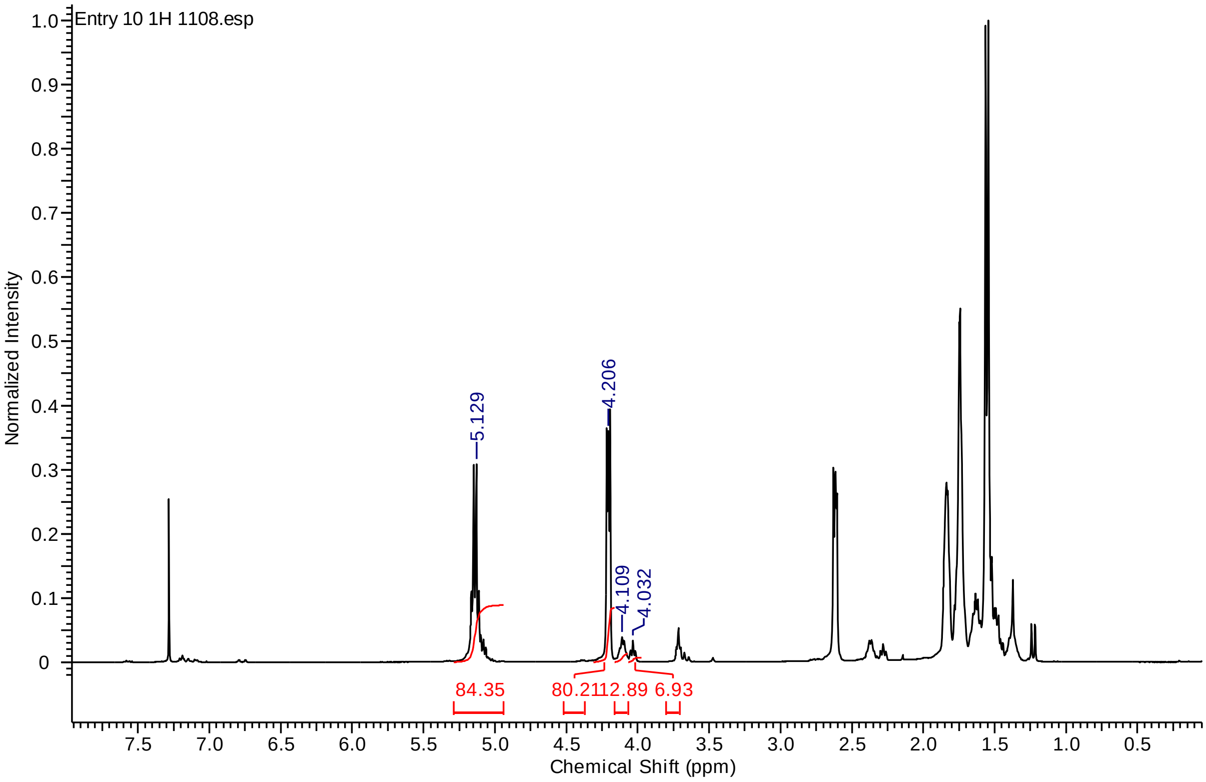

Figure S27: ^{1}H NMR Spectrum of polymer from Table 1, entry 11

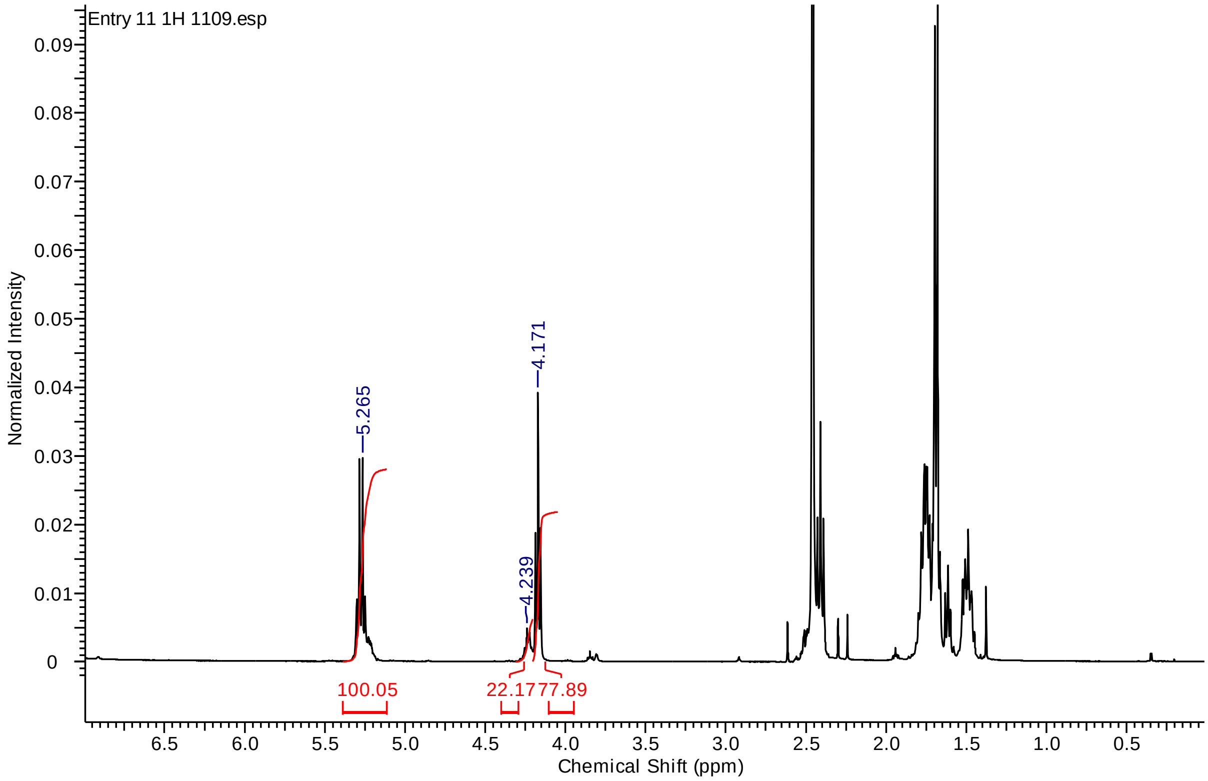

Figure S28: ^{1}H NMR Spectrum of polymer from Table 1, entry 12

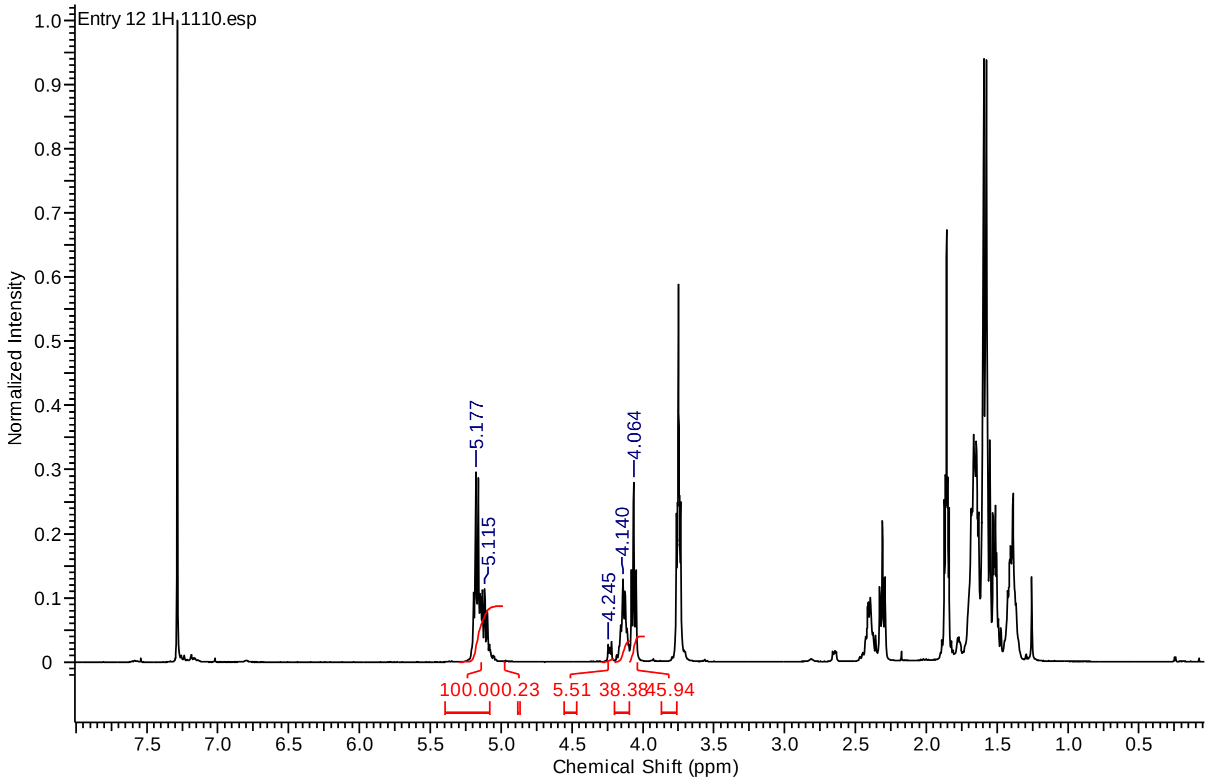

Figure S29: Carbonyl region of the ^{13}C NMR Spectrum (quantitative) of polymer from Table 1, entry 9

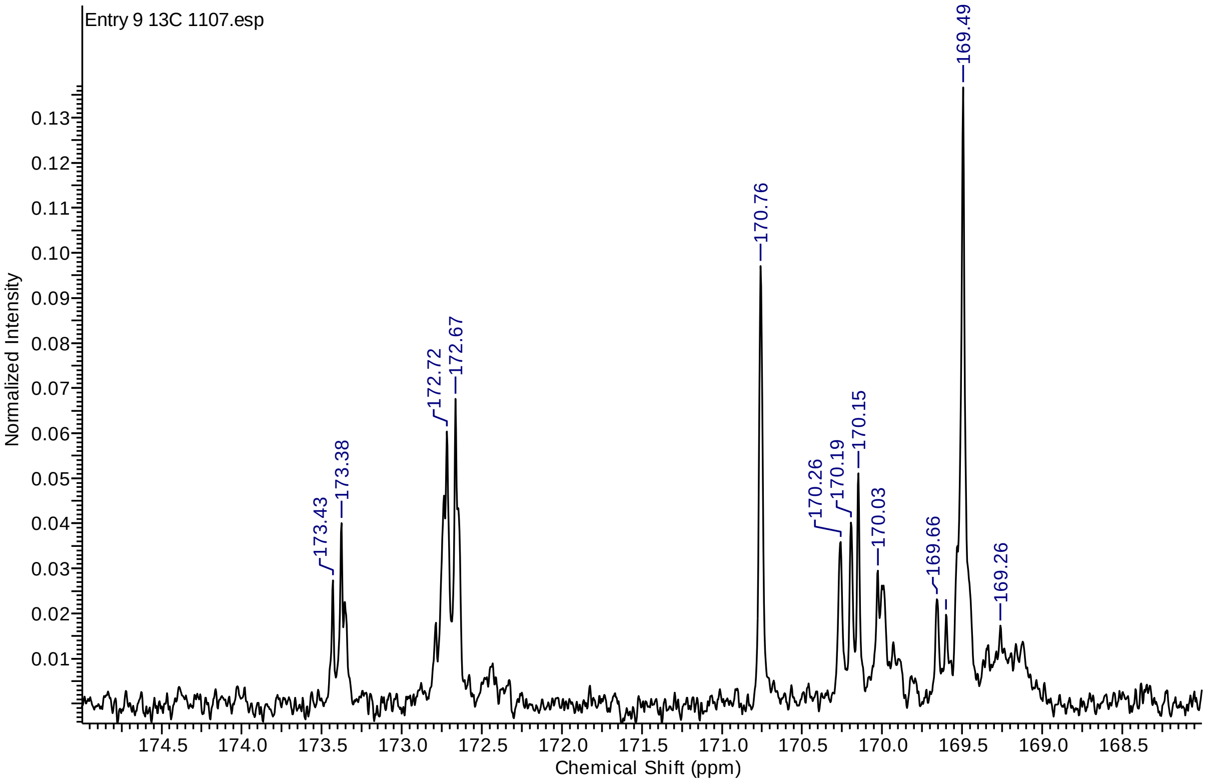

Figure S30: Carbonyl region of the ^{13}C NMR Spectrum (quantitative) of polymer from Table 1, entry 11

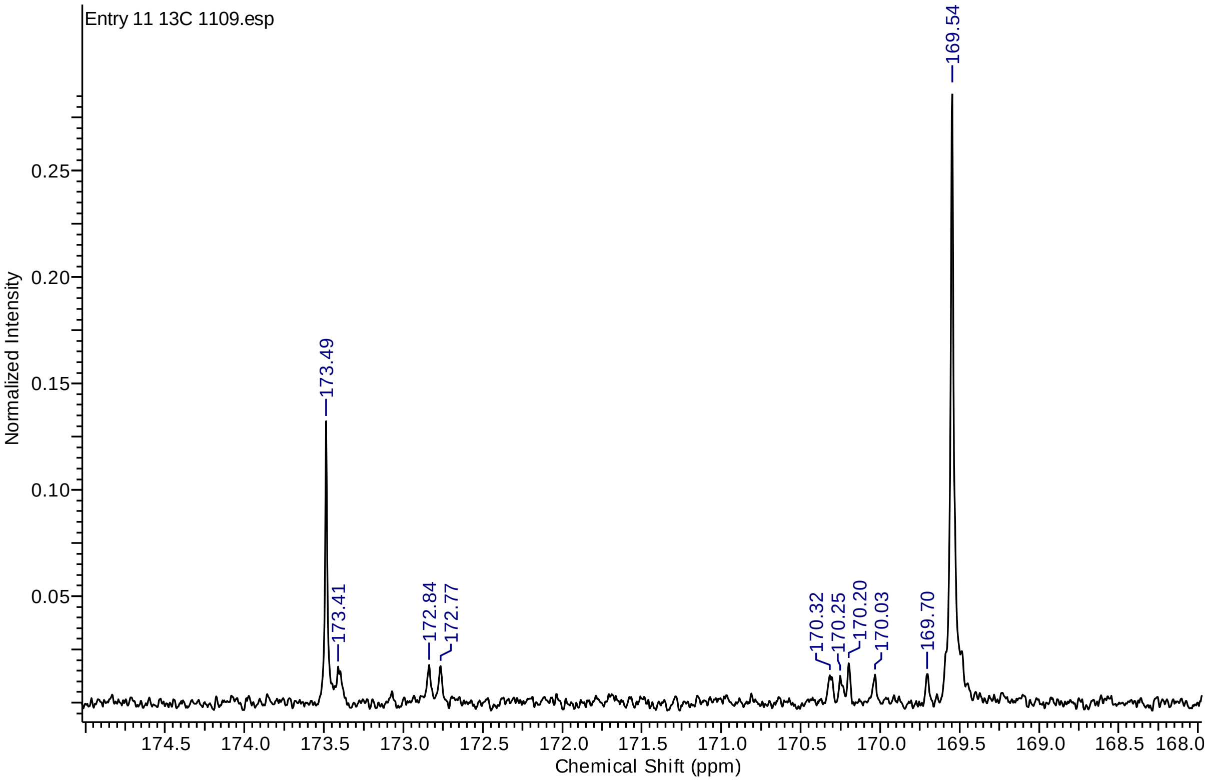

Figure S31: Carbonyl region of the ^{13}C NMR Spectrum (quantitative) of polymer from Table 1, entry 12

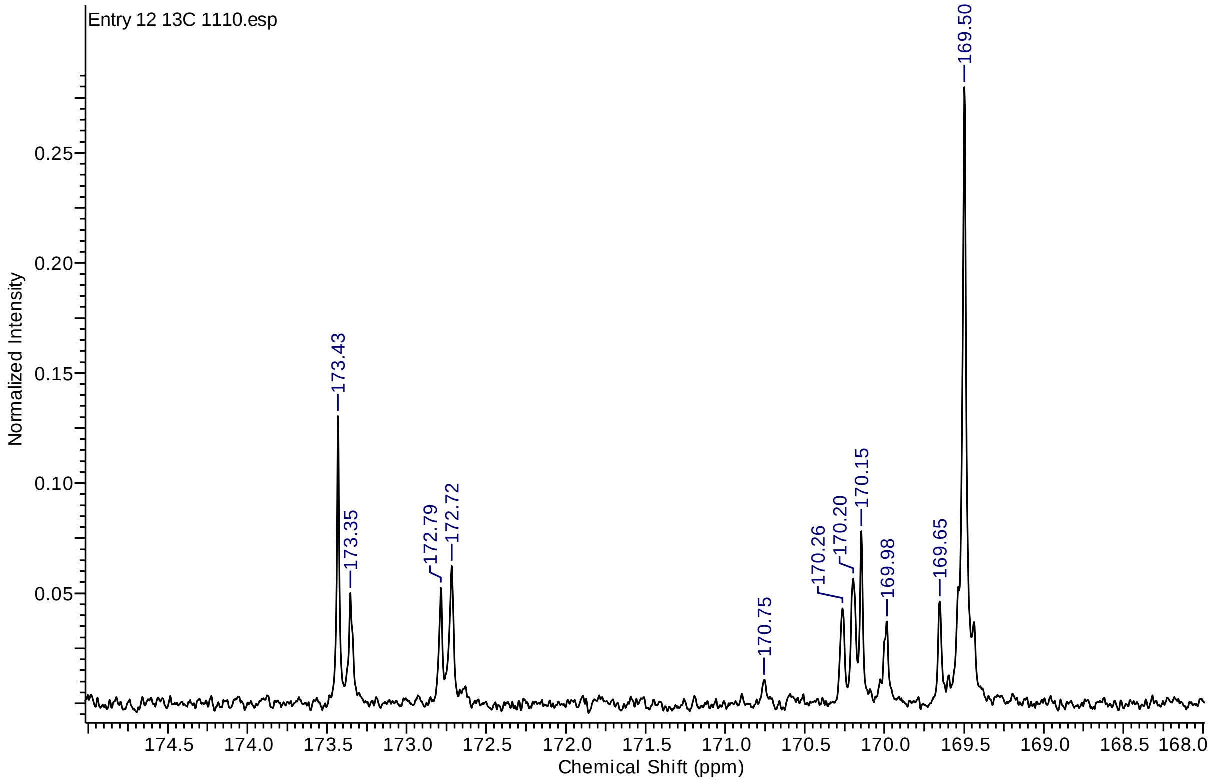

4. GPC Traces of Copolymers

Figure S32: GPC Trace of sample from Table 1 entry 1

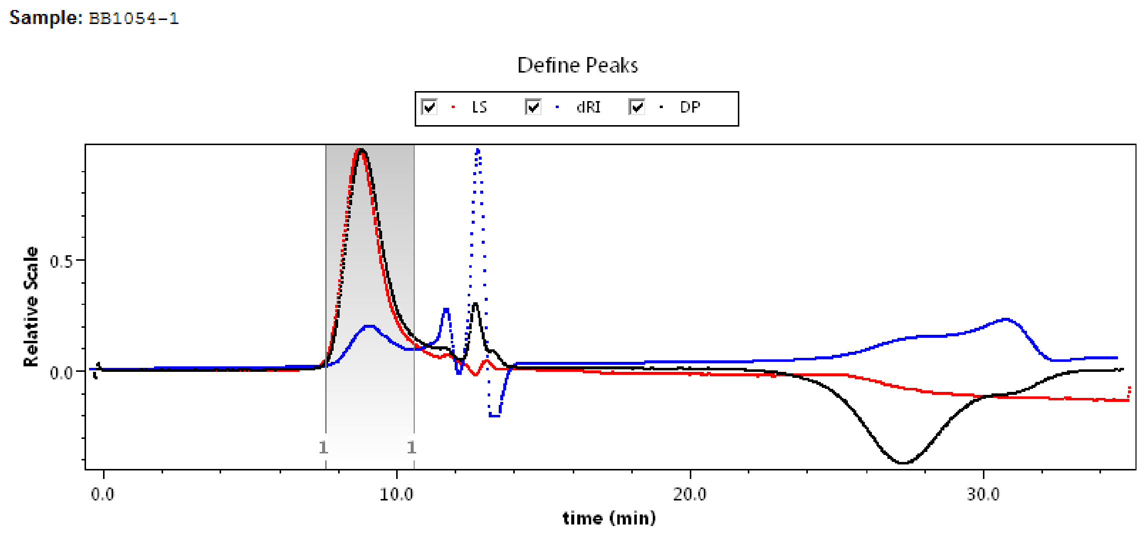

Figure S33: GPC Trace of sample from Table 1 entry 2

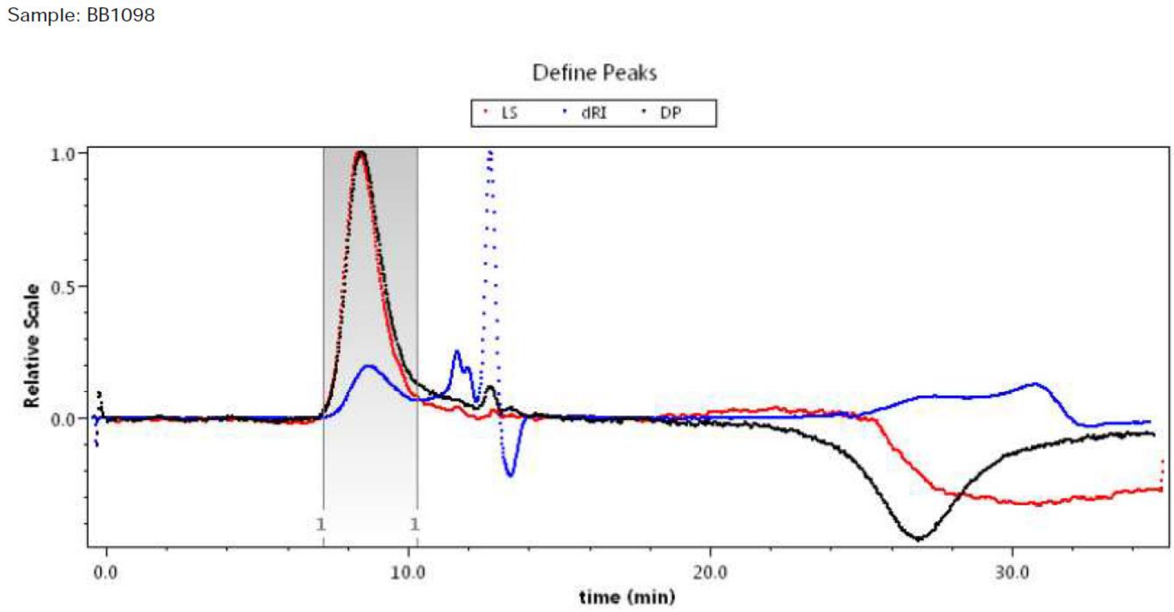

Figure S34: GPC Trace of sample from Table 1 entry 3

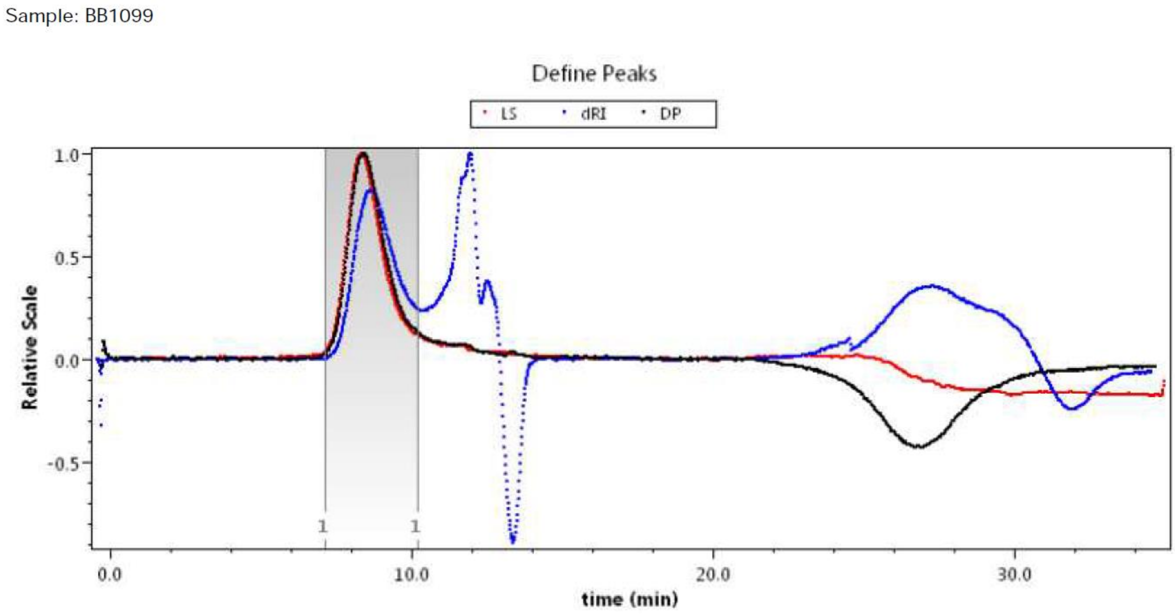

Figure S35: GPC Trace of sample from Table 1 entry 4

Sample: BB1100

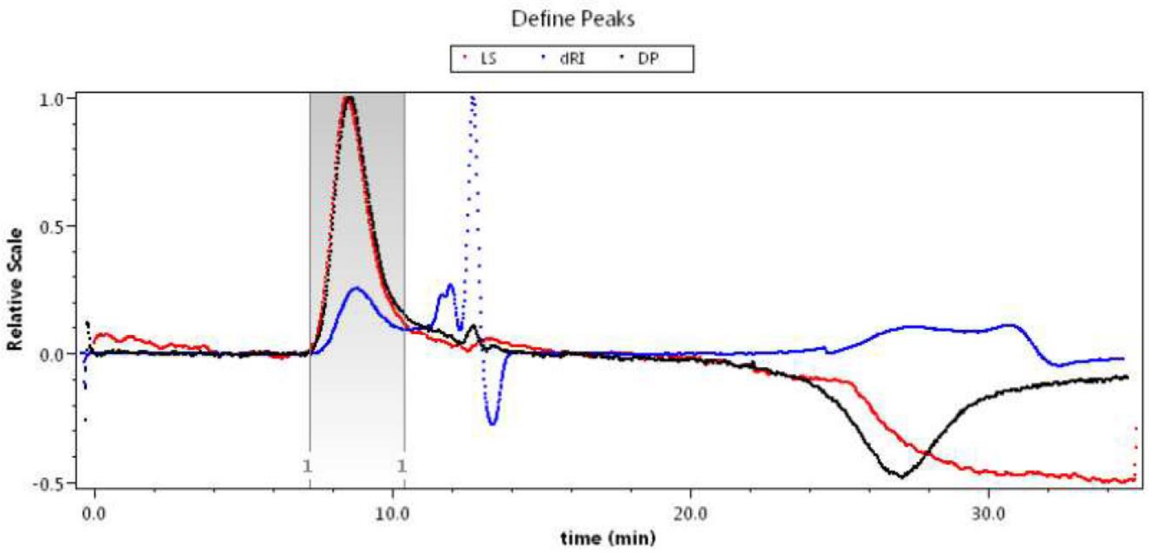

Figure S36: GPC Trace of sample from Table 1 entry 5

Sample: BB1101

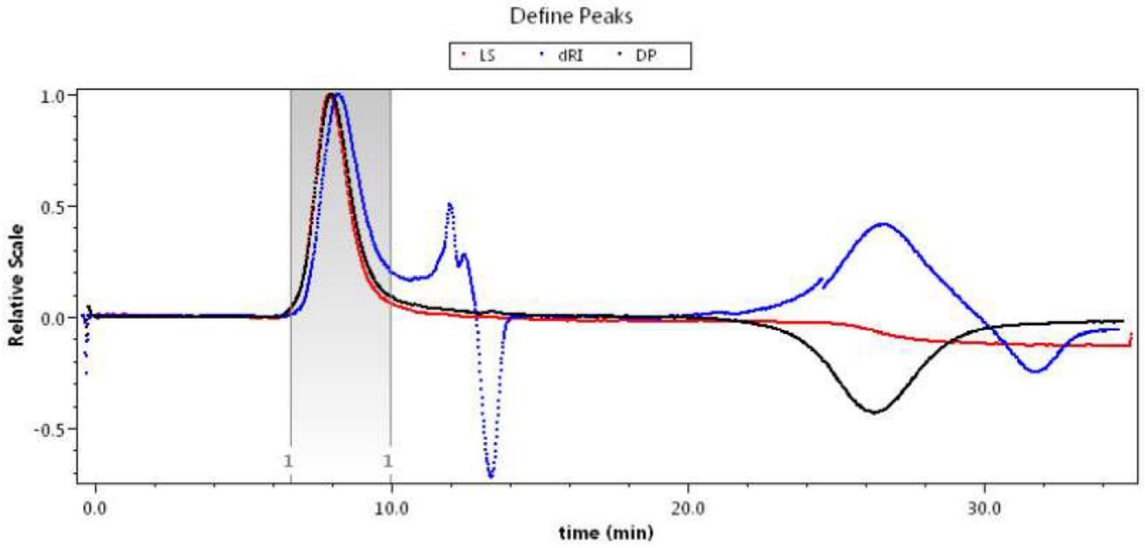

Figure S37: GPC Trace of sample from Table 1 entry 6

Sample: BB1102

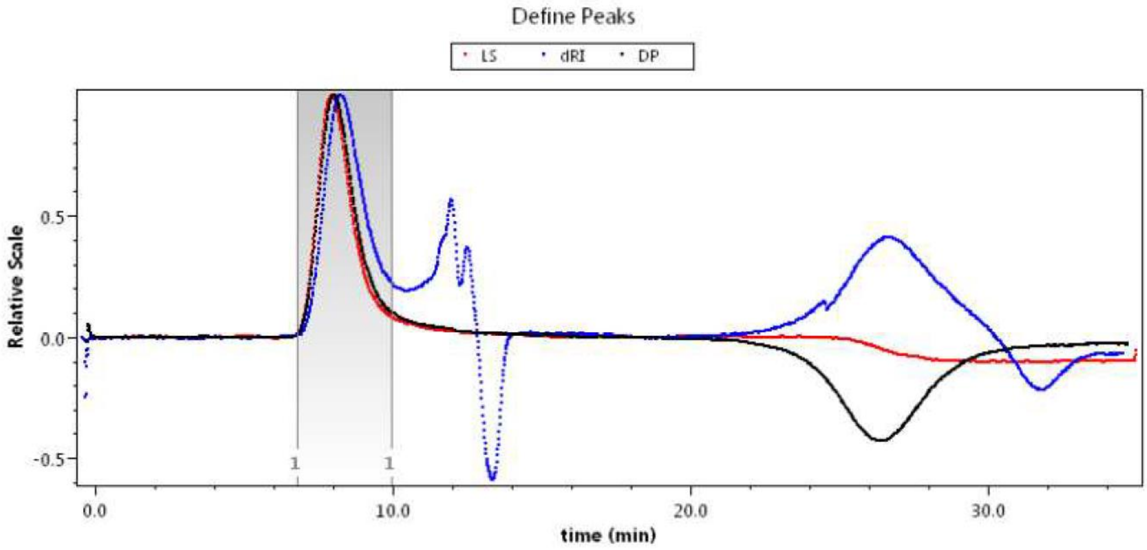

Figure S38: GPC Trace of sample from Table 1 entry 7

Sample: BB1105

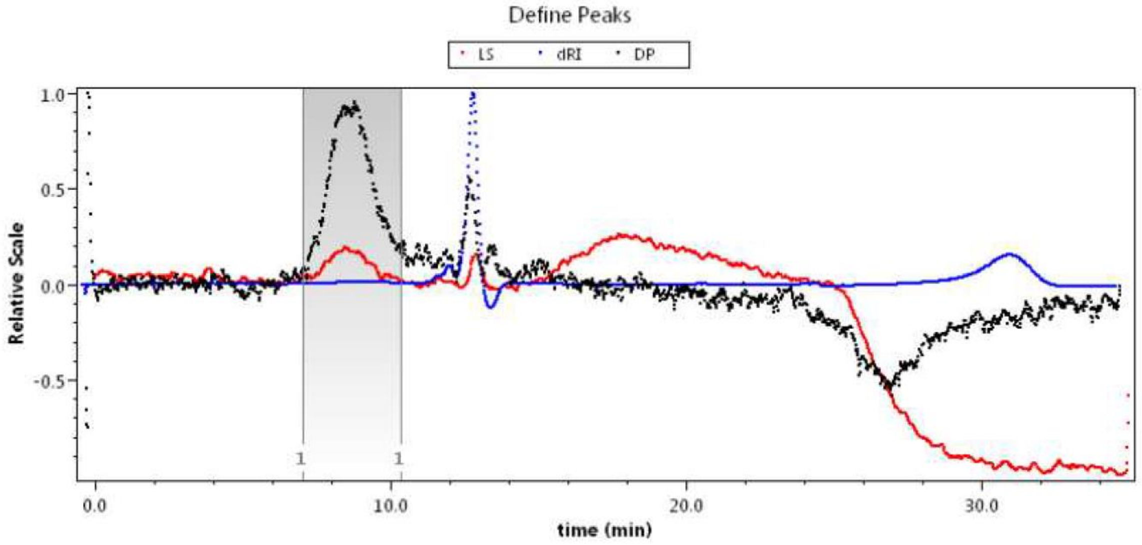

Figure S39: GPC Trace of sample from Table 1 entry 8

Sample: BB1106

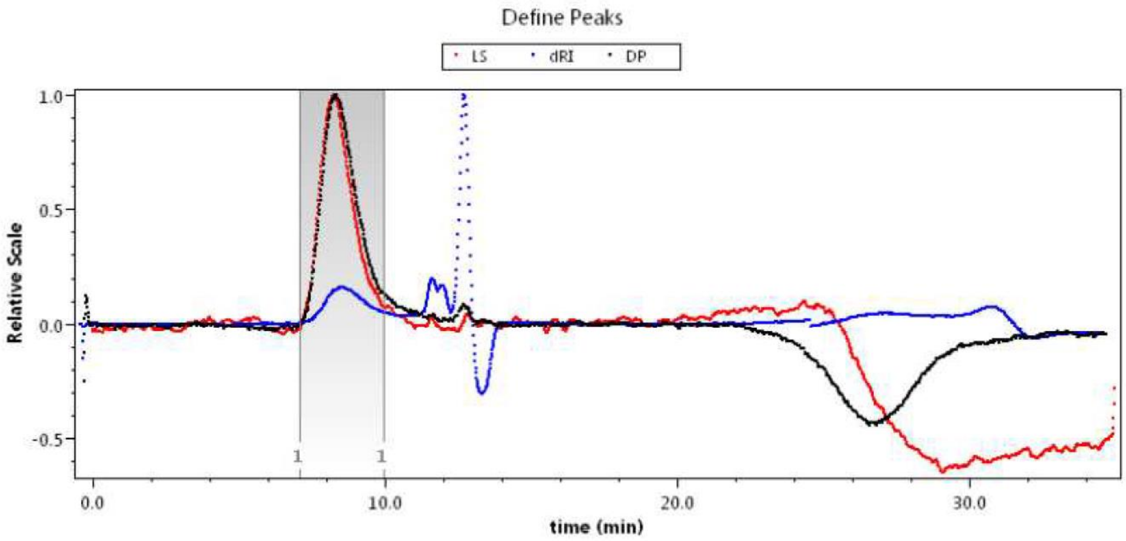

Figure S40: GPC Trace of sample from Table 1 entry 9

Sample: BB1107

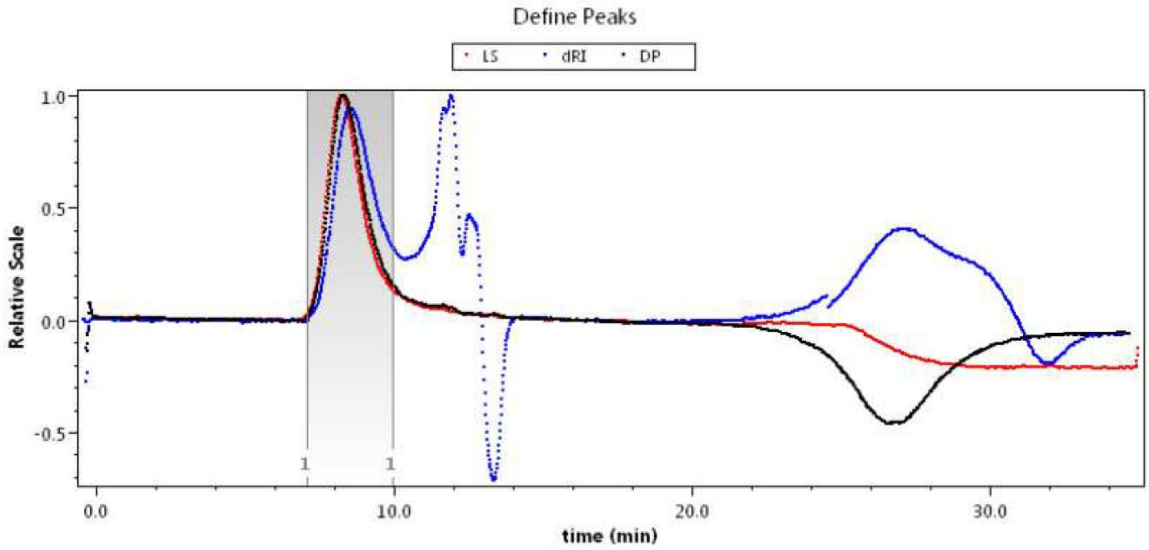

Figure S41: GPC Trace of sample from Table 1 entry 10

Sample: BB1108

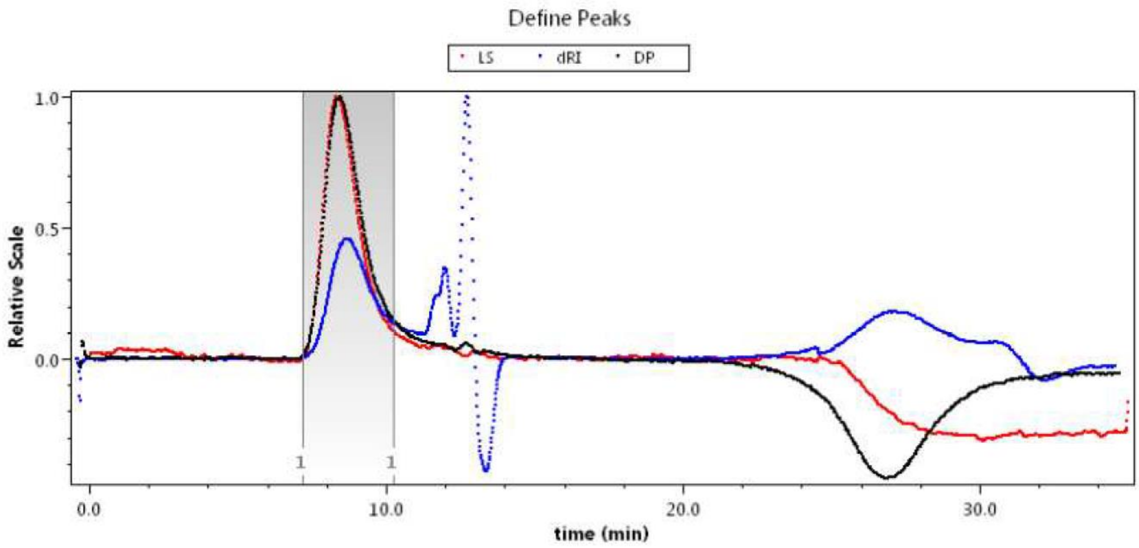

Figure S42: GPC Trace of sample from Table 1 entry 11

Sample: BB1109

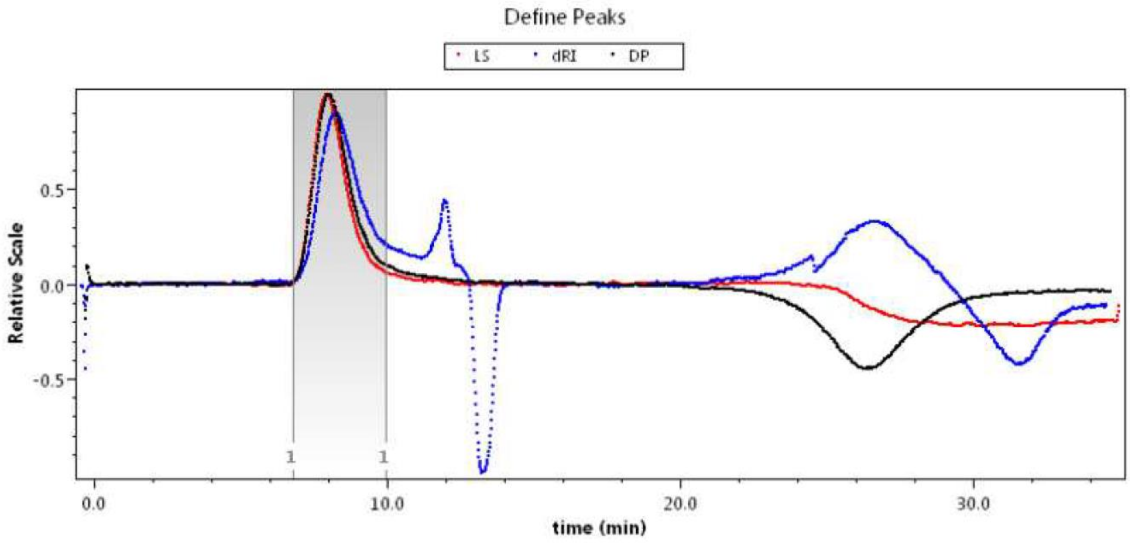

Figure S43: GPC Trace of sample from Table 1 entry 12

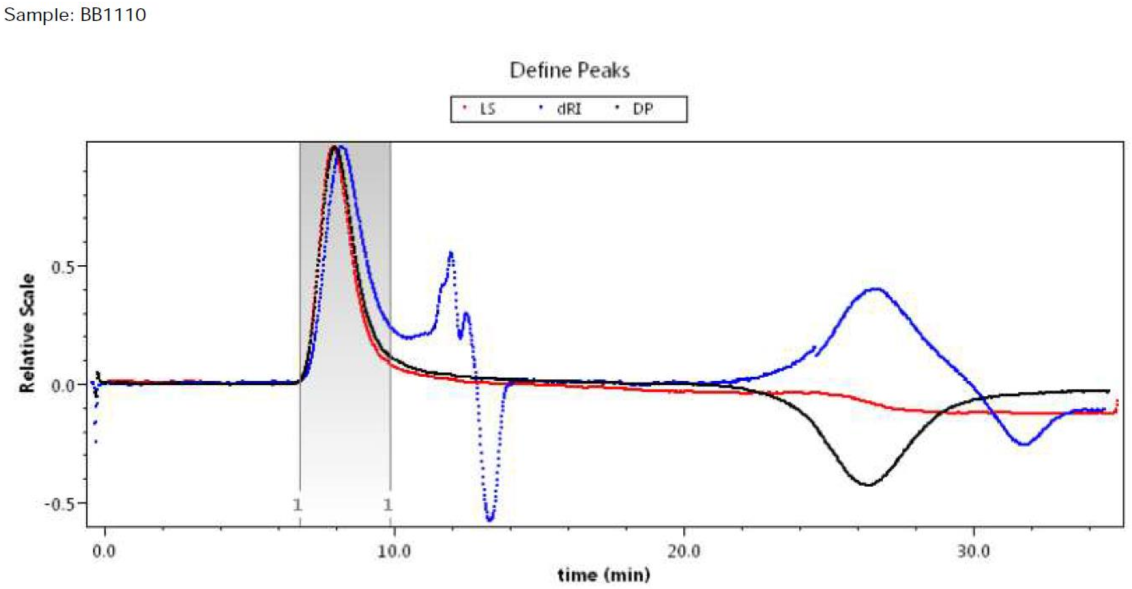

5. X-ray data

Table S3: Single Crystal X-ray Data

	Complex **1**	Complex **2**	Complex **3**[a]
CCDC reference	2260183	2260184	2260185
Empirical formula	$C_{57}H_{91}LaN_3O_3Si_2$	$C_{49}H_{73}LaN_4O_2Si_2$	$C_{48}H_{72}LaN_5O_2Si_2$
Formula weight	1061.41	945.2	946.19
Temperature/K	100(1)	100(1)	100(1)
Crystal system	triclinic	monoclinic	monoclinic
Crystal Description	Plate	Prism	Prism
Space group	P-1	$P2_1/c$	$P2_1/c$
a/Å	12.5847(2)	9.54890(10)	12.1758(3)
b/Å	14.6789(2)	36.5304(2)	25.4678(7)
c/Å	17.0872(4)	14.89280(10)	18.5937(4)
α/°	98.3879(17)	90	90
β/°	102.1945(18)	105.5080(10)	100.303(2)
γ/°	105.5303(13)	90	90
Volume/Å^3	2903.16(9)	5005.85(7)	5672.8(2)
Z	2	4	4
ρ_{calc} g/cm^3	1.214	1.254	1.108
μ/mm^{-1}	6.397	7.351	6.492
F(000)	1126	1984	1984.0
Crystal size/mm^3	0.134 × 0.084 × 0.034	0.33 × 0.21 × 0.08	0.159 × 0.075 × 0.022
Radiation	CuKα (λ = 1.54184)	Cu Kα (λ = 1.54184)	Cu Kα (λ = 1.54184)
2Θ range for data collection/°	5.418 to 155.53	7.834 to 152.742	5.948 to 154.622
Index ranges	-15 ≤ h ≤ 15, -18 ≤ k ≤ 18, -21 ≤ l ≤ 21	-11 ≤ h ≤ 12, -38 ≤ k ≤ 45, -18 ≤ l ≤ 17	-14 ≤ h ≤ 15, -28 ≤ k ≤ 31, -23 ≤ l ≤ 18

Reflections collected	48246	92605	31992
Independent reflections	12109 [R_{int} = 0.0720, R_{sigma} = 0.0552]	10430 [R_{int} = 0.0629, R_{sigma} = 0.0247]	11707 [R_{int} = 0.0371, R_{sigma} = 0.0436]
Data/restraints/parameters	12109/1/616	10430/42/574	11707/49/549
Goodness-of-fit on F^2	1.043	1.268	1.057
Final R indexes [I>=2σ (I)]	R_1 = 0.0417, wR_2 = 0.1017	R_1 = 0.0614, wR_2 = 0.1344	R_1 = 0.1264, wR_2 = 0.2811
Final R indexes [all data]	R_1 = 0.0456, wR_2 = 0.1040	R_1 = 0.0619, wR_2 = 0.1346	R_1 = 0.1334, wR_2 = 0.2849
Largest diff. peak/hole / $e^- Å^{-3}$	1.27/-1.98	1.24/-2.35	3.46/-2.74

Data can be obtained from: www.ccdc.cam.ac.uk

[a]The structure of 3 was found to contain a high level of disorder, particularly of the dimethylsilyl groups, and efforts to model this were unsuccessful, leading to large thermal ellipsoids, a high R1 and high residual electron density. Additionally, a solvent mask was applied to account for 1.5 molecules of disordered toluene per unit cell.

6. MALDI-ToF Data

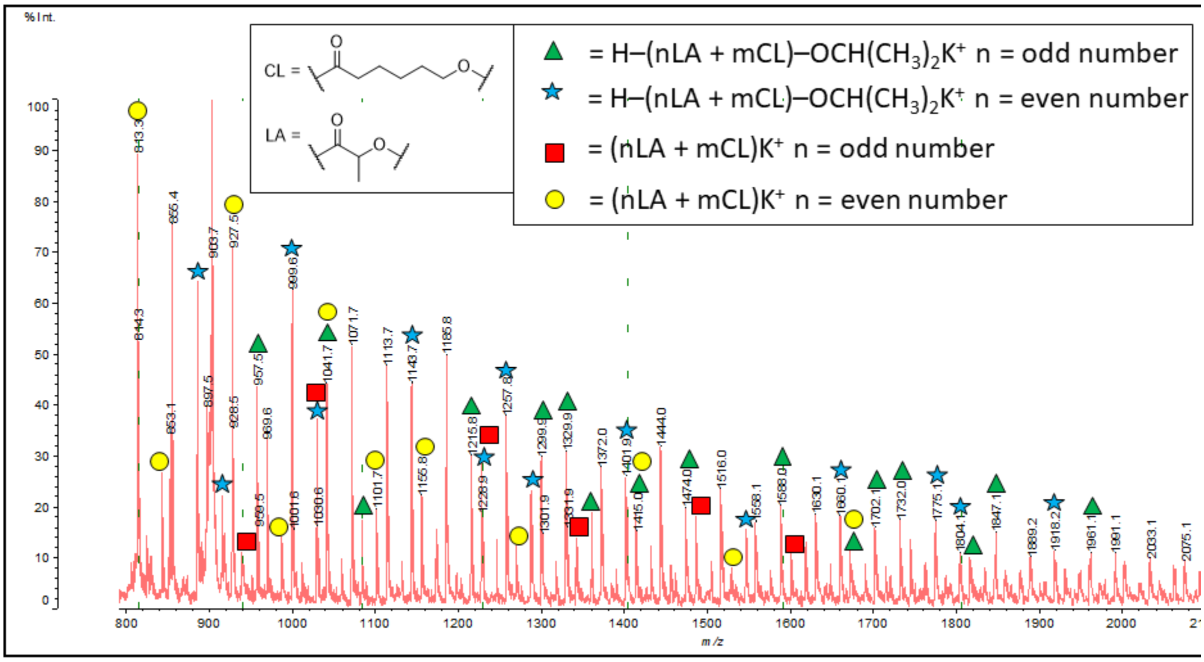

Figure S43: MALDI-ToF spectrum of a copolymer formed by reaction of complex **2** with LA and ε-CL with $[LA]_0$=$[ε\text{-}CL]_0$= 0.4 M and [**2**]:$[LA]_0$:$[ε\text{-}CL]_0$ = 1:20:20 in toluene.

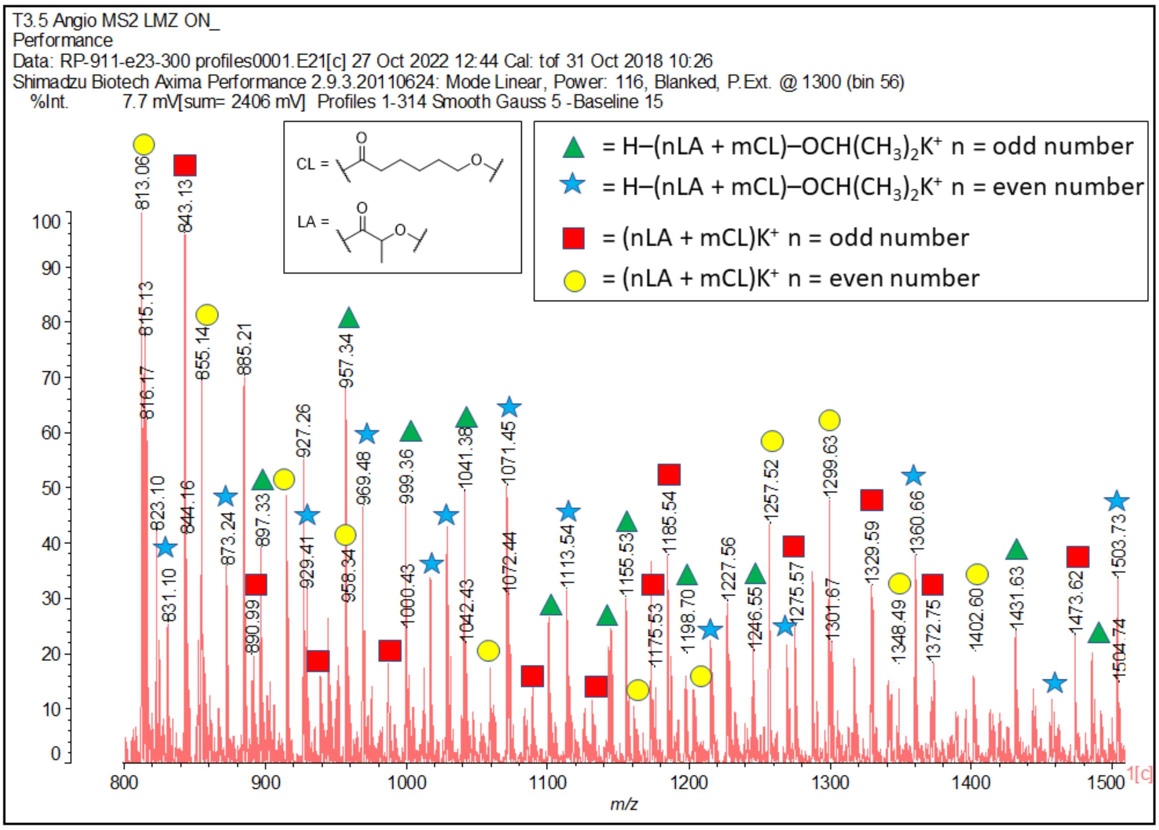

Figure S44: MALDI-ToF spectrum of a copolymer formed by reaction of reaction of complex **3** with LA and ε-CL in the presence of 1 equiv ^{i}PrOH. $[LA]_0$=[ε-CL]$_0$= 0.25 M and [**3**]:$[LA]_0$:[ε-CL]$_0$ = 1:5:5 in toluene.

7. Additional Experiments

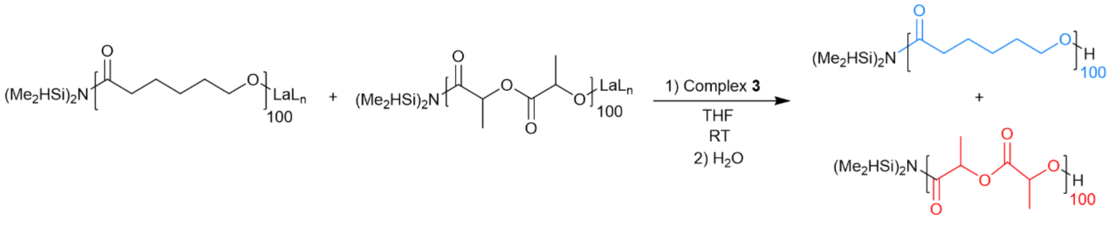

Scheme S1: Copolymerisation of active PLA and PCL chains promoted by complex **3** in THF.

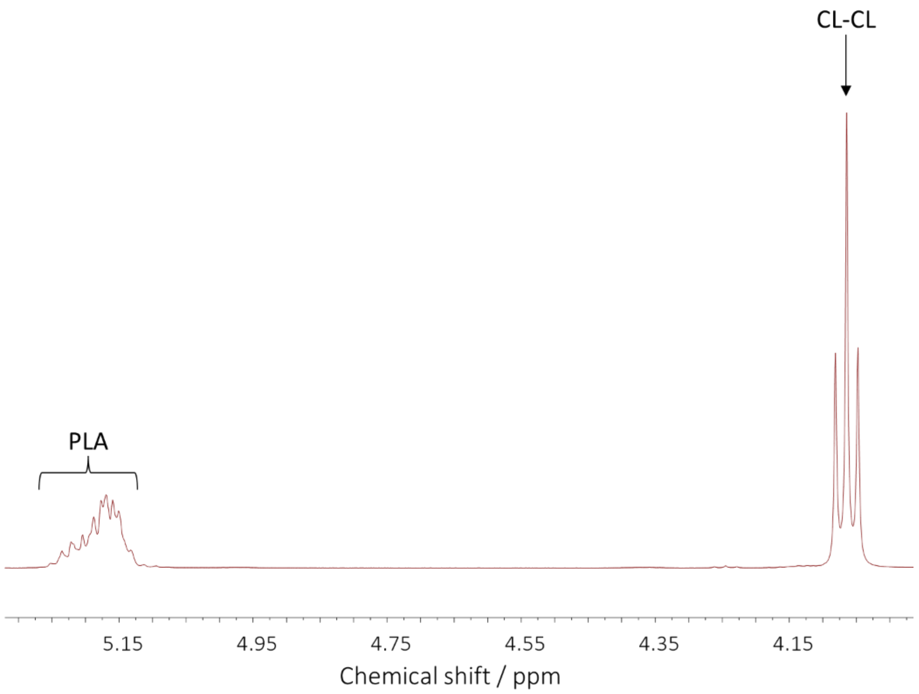

Figure S45: ^{1}H NMR spectrum of the methine and methylene region ($CDCl_3$) of the copolymerisation of active PCL and PLA chains using complex **3** in THF.

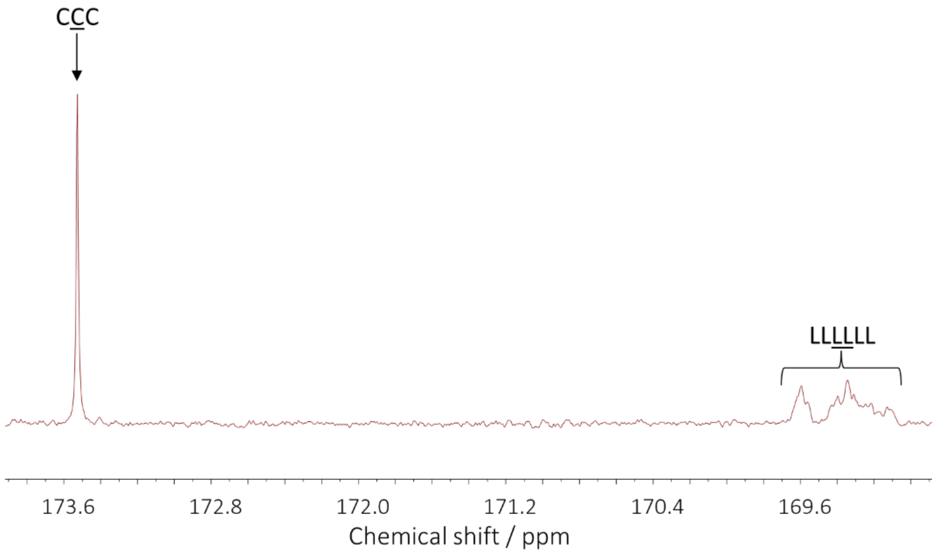

Figure S46: Quantitative ^{13}C NMR spectrum of the carbonyl region ($CDCl_3$) of the copolymerisation of active PCL and PLA chains using complex **3** in THF.

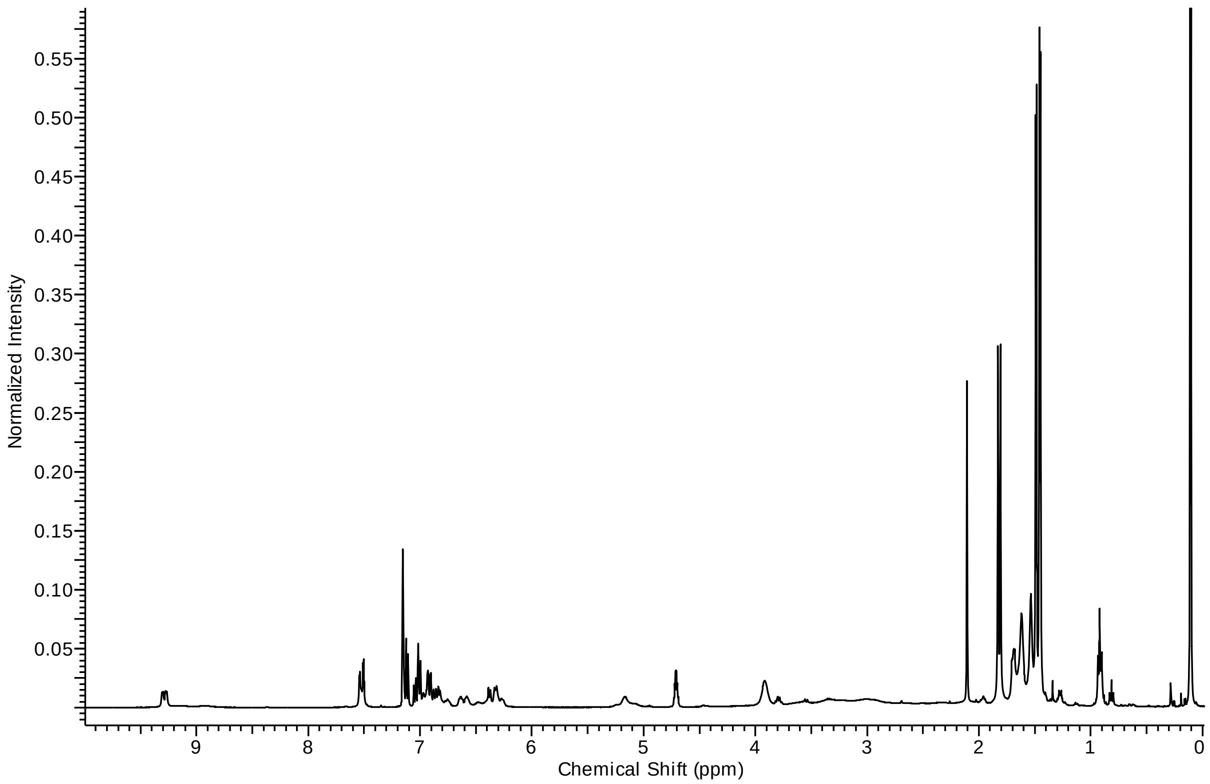

Figure S47: ${}^{1}H$ NMR spectrum of the NMR scale reaction between **3** and 1 equiv (*S*)-ethyl-lactate in benzene-d_6.

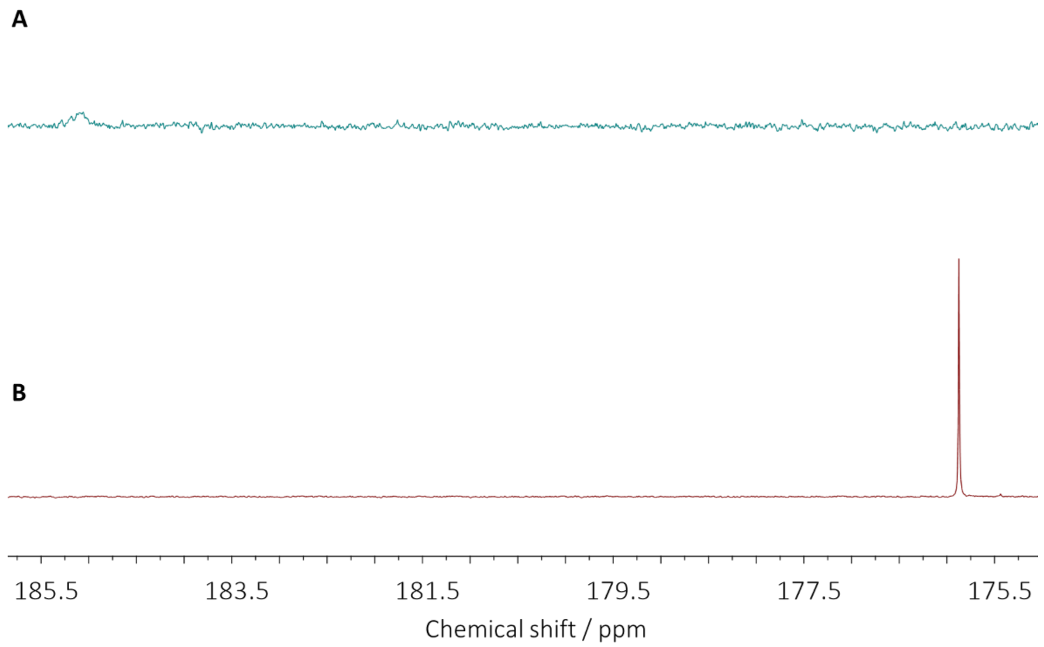

Figure S48: (A) The carbonyl region of the ${}^{13}C$ NMR spectrum of the NMR scale reaction between **3** and 1 equiv (*S*)-ethyl-lactate in benzene-d_6 and (B) The carbonyl region of the ${}^{13}C$ NMR spectrum of (*S*)-ethyl-lactate in benzene-d_6 for comparison.

Figure S49: ^{1}H DOSY NMR spectrum of **1**
Data processed using Dynamics Centre software

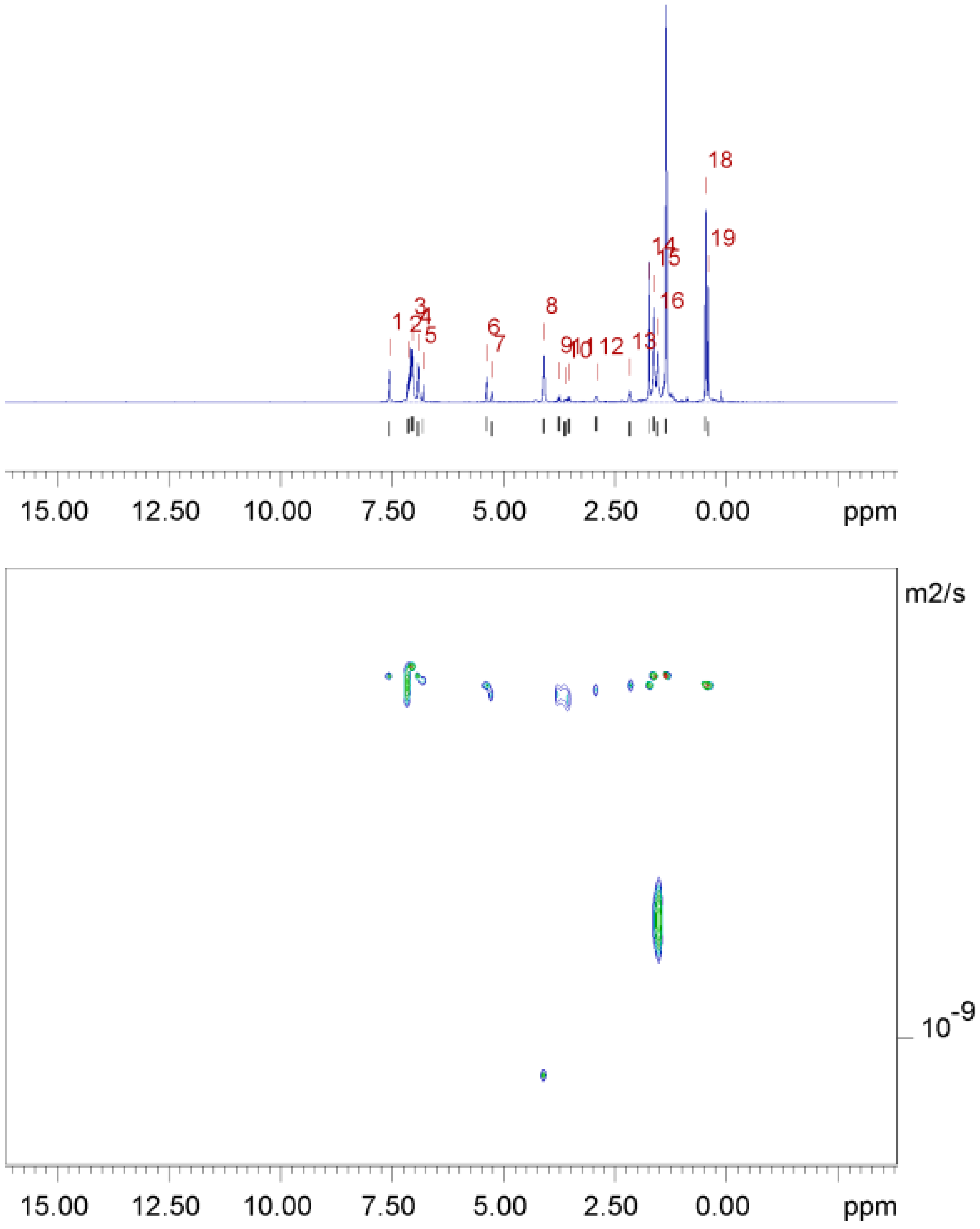

Figure S50: ^{1}H DOSY NMR spectrum of **2**
Data processed using Dynamics Centre software

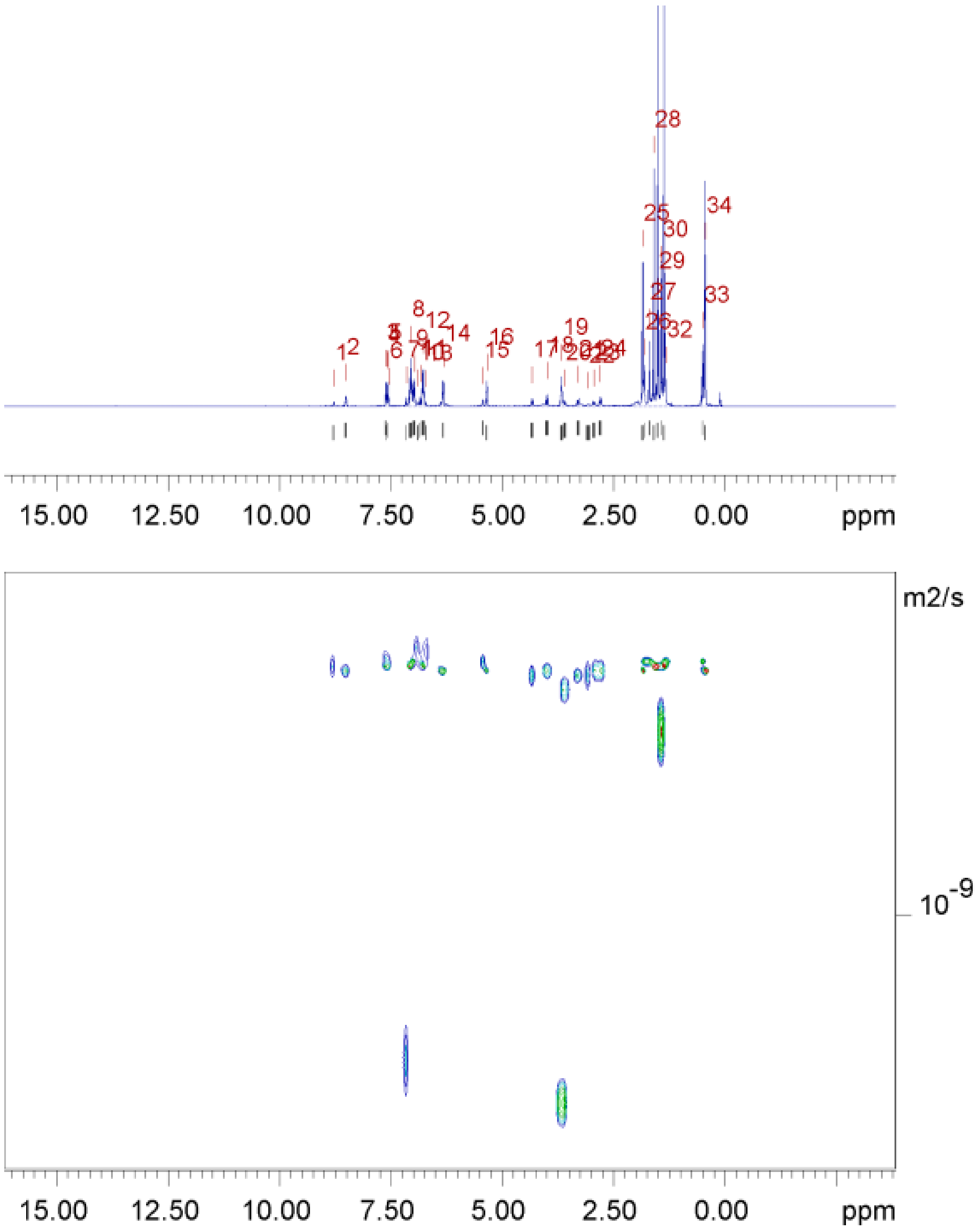

Figure S51: ^{1}H DOSY NMR spectrum of **3**
Data processed using Dynamics Centre software

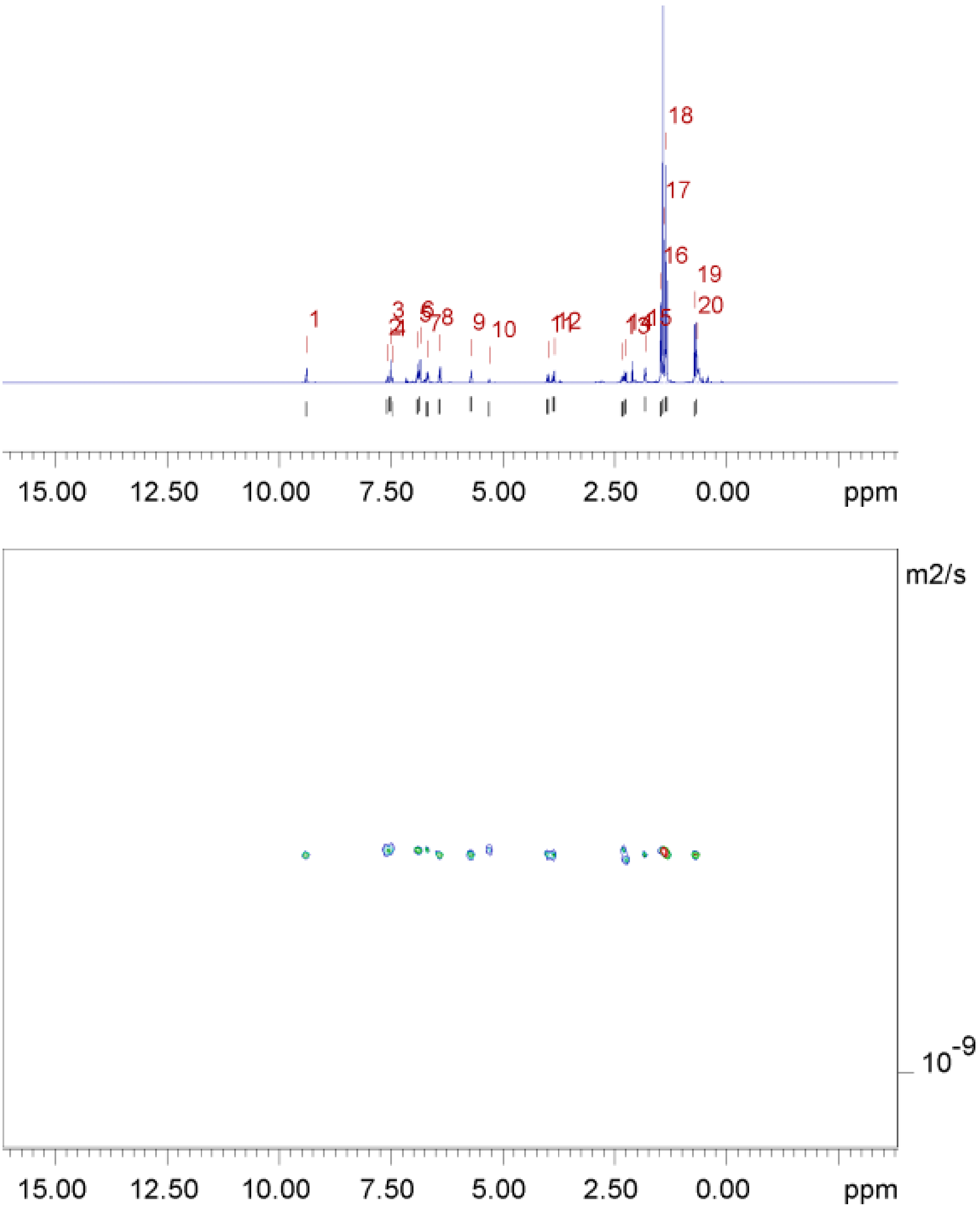
18
17
16
19
20
1
3
2
4
6
5
7
8
9
10
11
12
13
14
15
15.00
12.50
10.00
7.50
5.00
2.50
0.00
ppm
m2/s
10-9

Figure S52: Showing ^{1}H NMR spectra from the NMR scale reaction of complex **1** with 1 equivalent $Ph_3P=O$ in C_6D_6.

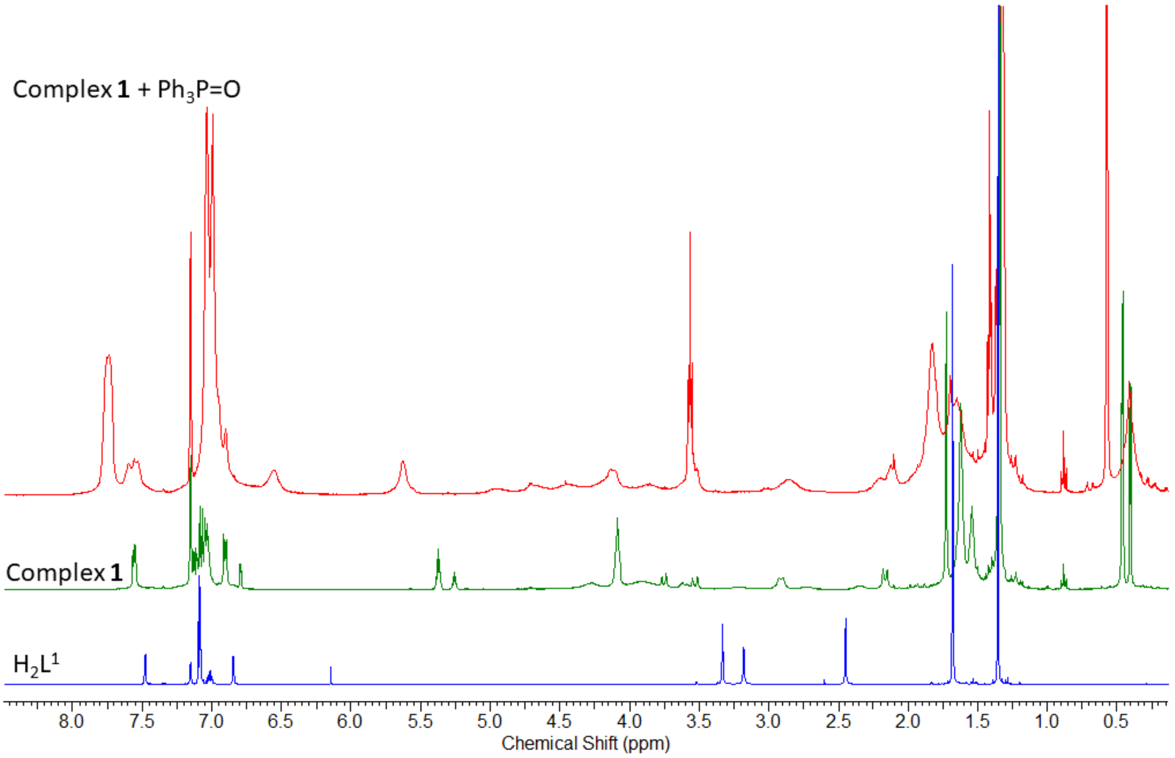

Figure S53: Showing ^{1}H NMR spectra from the NMR scale reaction of complex **2** with 1 equivalent $Ph_3P=O$ in C_6D_6.

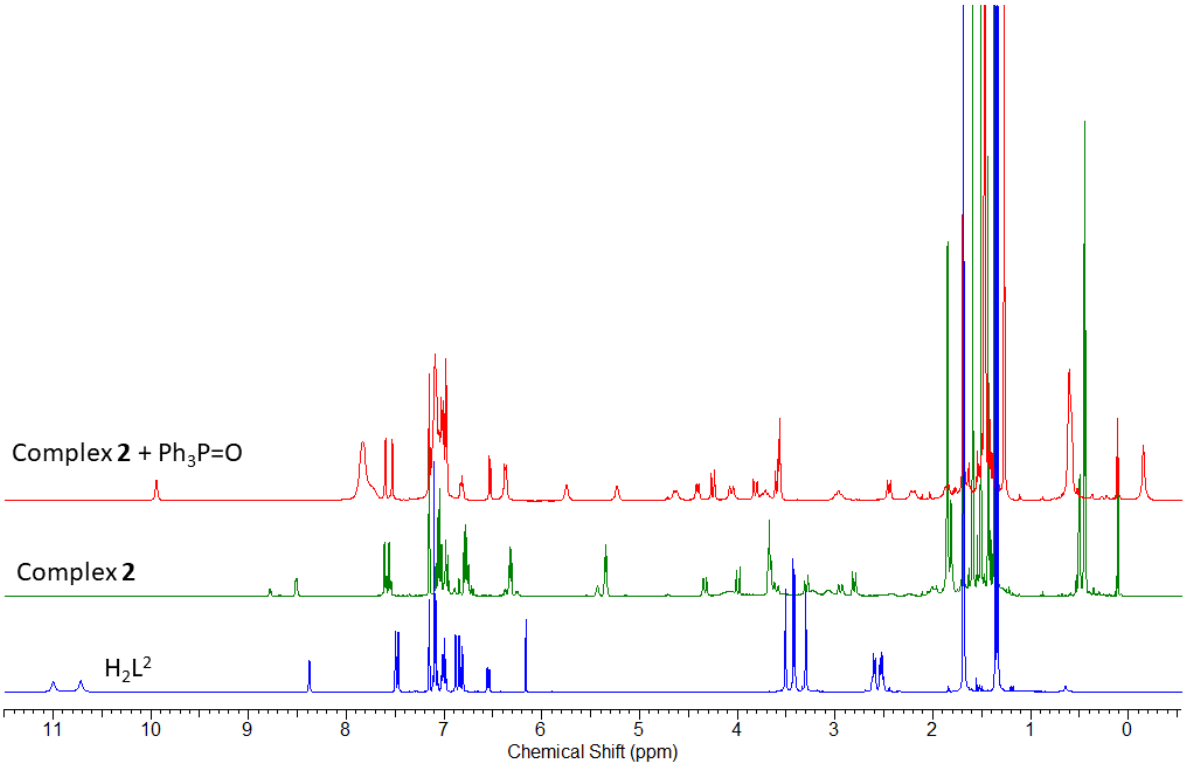

Figure S54: Showing ^{1}H NMR spectra from the NMR scale reaction of complex **3** with 1 equivalent $Ph_3P=O$ in C_6D_6.

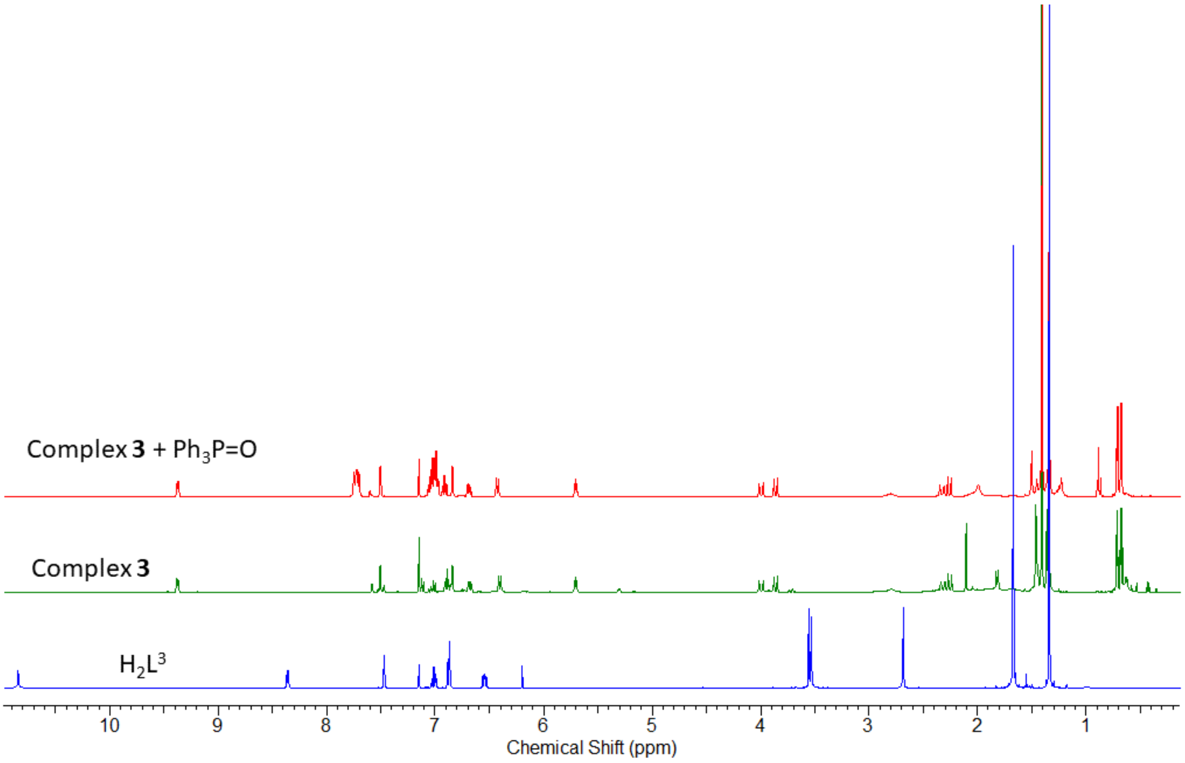

Figure S55: Showing $^{31}P\{^{1}H\}$ NMR spectra from the NMR scale reaction of complexes **1**-**3** with 1 equivalent $Ph_3P=O$ in C_6D_6.

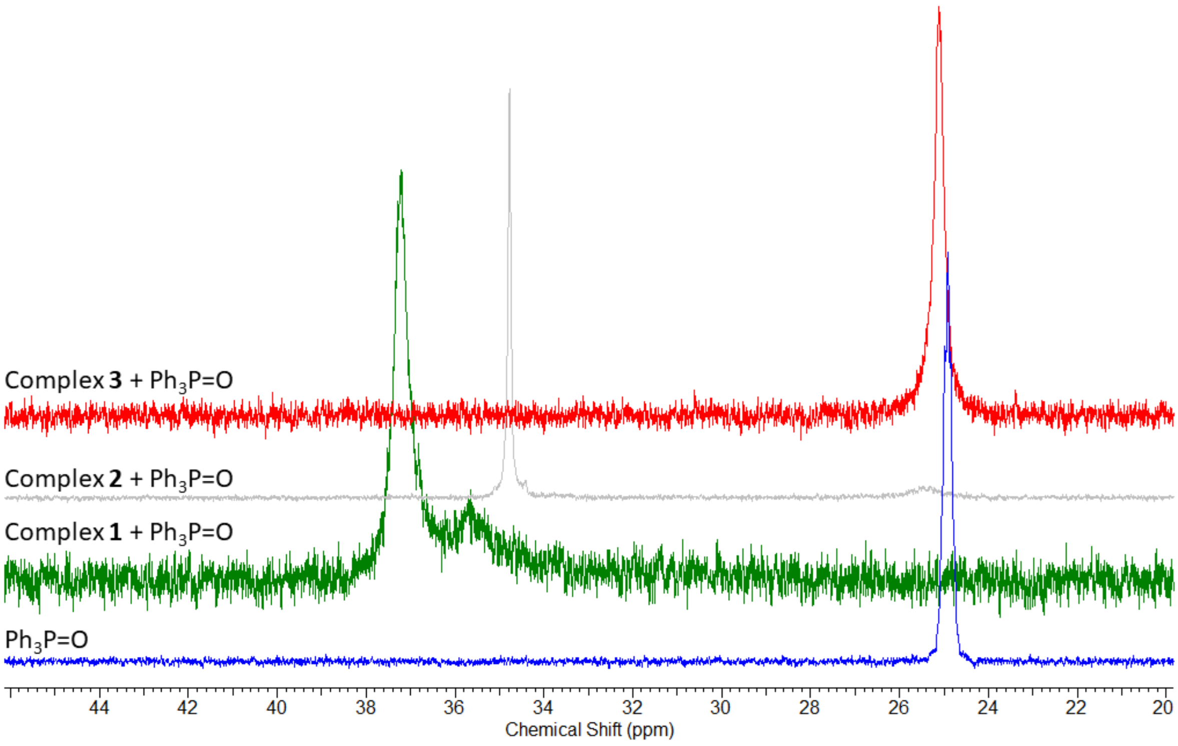

Table S4: Thermal characterisation data for selected copolymers.

Entry	Initiator [I]	Solvent	CL/LA (mol %)[b]	T_g (°C)[c]	$T_g$theo (°C)[d]
1	1	Toluene	13/87	38.5	33.7
2	1	THF	9/91	42.1	39.5
3	2	Toluene	50/50	-14.8	-14.6
4	2	THF	17/83	30.8	28.1
5	3	Toluene	61/39	-27.9/50.1	-27.4
6	3	THF	48/52	-8.5	-12.2

[a]Conditions: $[LA]_0$ = $[CL]_0$ = 1.0M, ($[LA]_0$ + $[CL]_0$)/[I] = 200, room temperature. [b]Determined by ^1H NMR spectroscopy at 400 MHz in $CDCl_3$. [c]Determined by DSC analysis. Data was obtained from the second heating cycle using heating rate of 10 °C min^{-1} [d]$T_g$theo values calculated using the Fox equation.

Figure S56: ^1H NMR spectrum of a copolymer prepared by complex **2** in toluene* for DOSY analysis

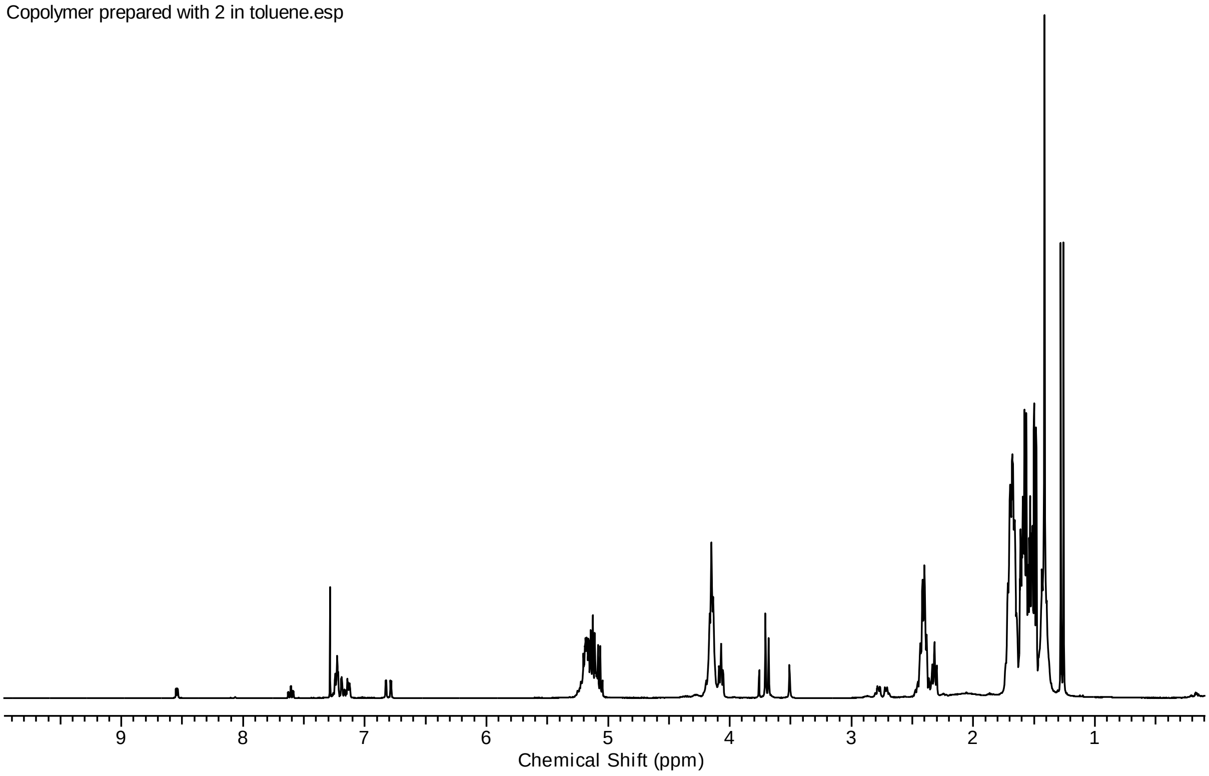

*Reaction conditions: $[(S,S)\text{-LA}]_0$ = $[\varepsilon\text{-CL}]_0$ = 0.5 M in toluene, $[(S,S)\text{-LA}]_0$/[**2**] = 10, reaction time of 20 min.

Figure S57: ^{1}H DOSY NMR spectrum of a copolymer prepared by complex **2** in toluene

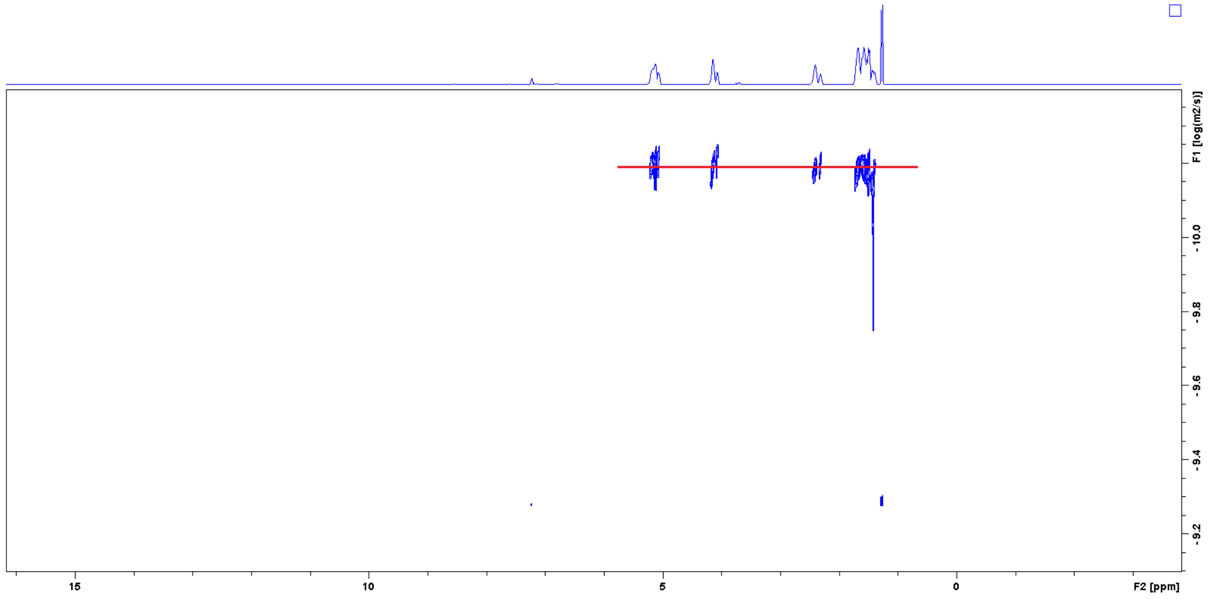

Figure S58: GPC trace of a copolymer prepared by complex **2** in toluene for DOSY analysis

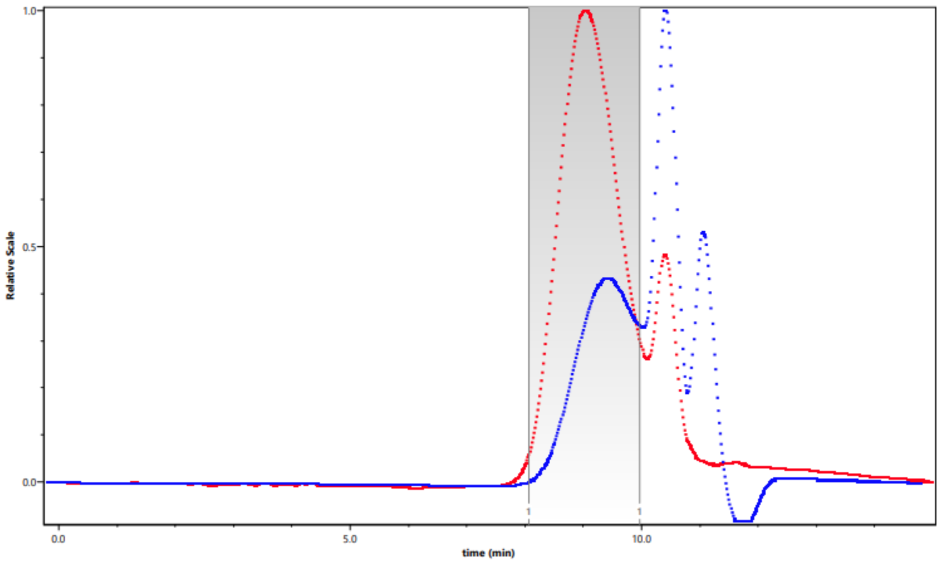

M_n = 6.3 kg mol^{-1}, $Đ$ = 1.21.

Figure S59: ^{1}H NMR spectrum of a copolymer prepared by complex **3** in THF* for DOSY analysis

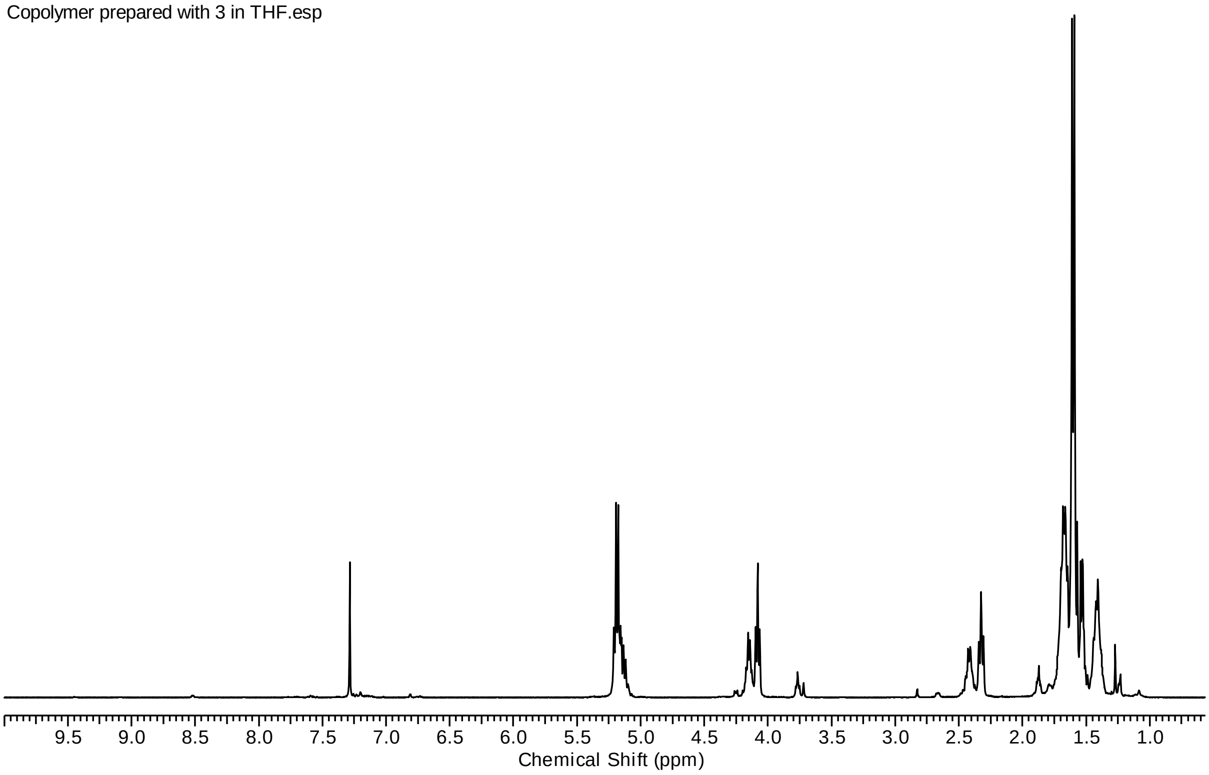

*Reaction conditions: [(*S,S*)-LA]$_0$ = [ε-CL]$_0$ = 0.5 M in toluene, [(*S,S*)-LA]$_0$/[**3**] = 10, reaction time of 20 min.

Figure S60: ^{1}H DOSY NMR spectrum of a copolymer prepared by complex **3** in THF

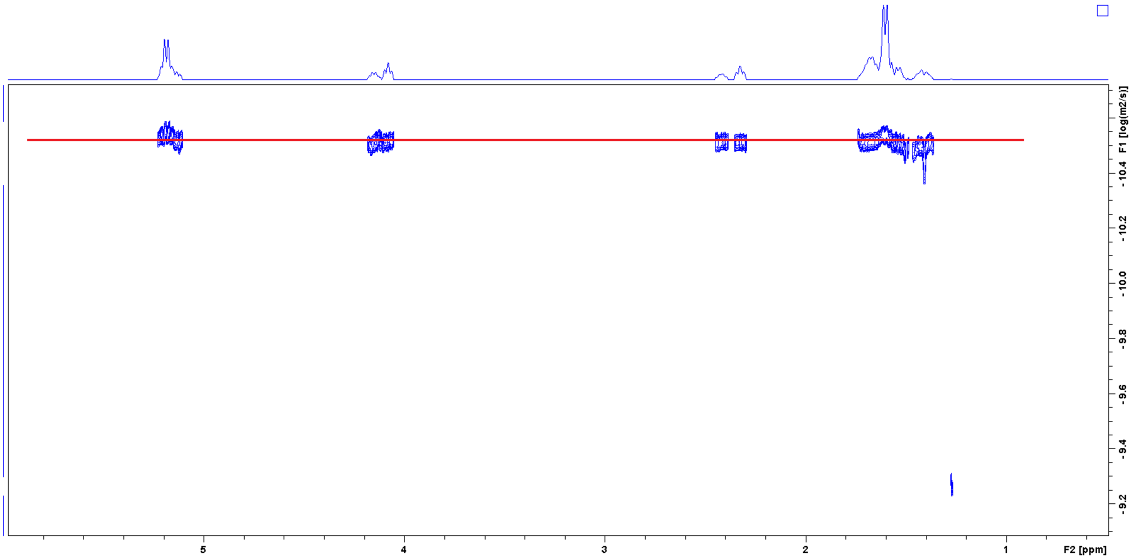

Figure S61: GPC trace of a copolymer prepared by complex **3** in THF for DOSY analysis

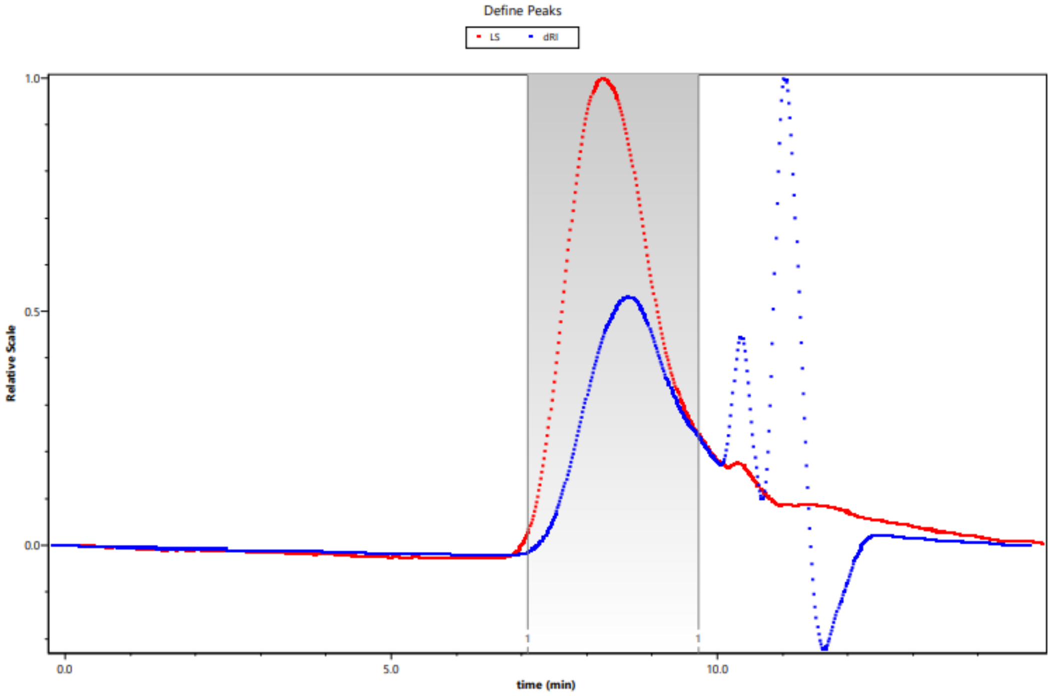

M_n = 21.5 kg mol^{-1}, $Đ$ = 1.16.

Figure S62: ^{1}H NMR spectrum of a copolymer prepared by complex **3** in toluene* for DOSY analysis

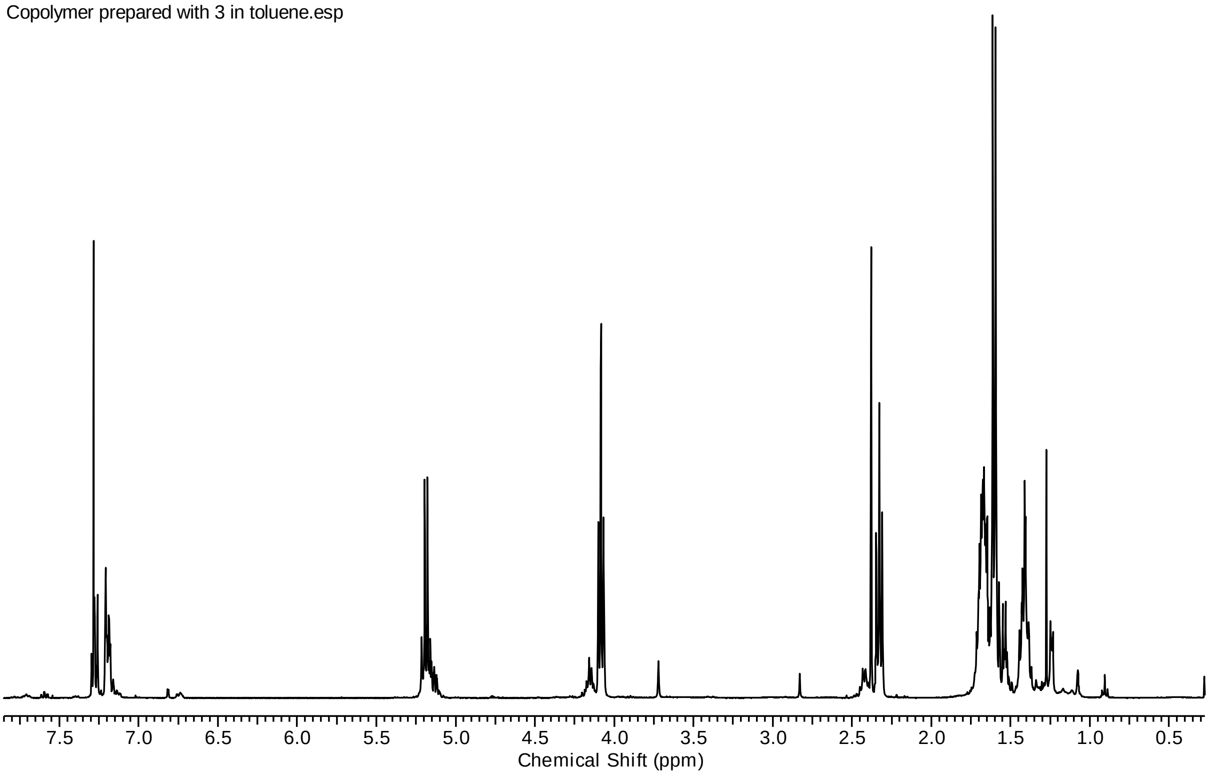

*Reaction conditions: [(*S*,*S*)-LA]$_0$ = [ε-CL]$_0$ = 0.5 M in toluene, [(*S*,*S*)-LA]$_0$/[**3**] = 10, reaction time of 20 min.

Figure S63: ^{1}H DOSY NMR spectrum of a copolymer prepared by complex **3** in toluene

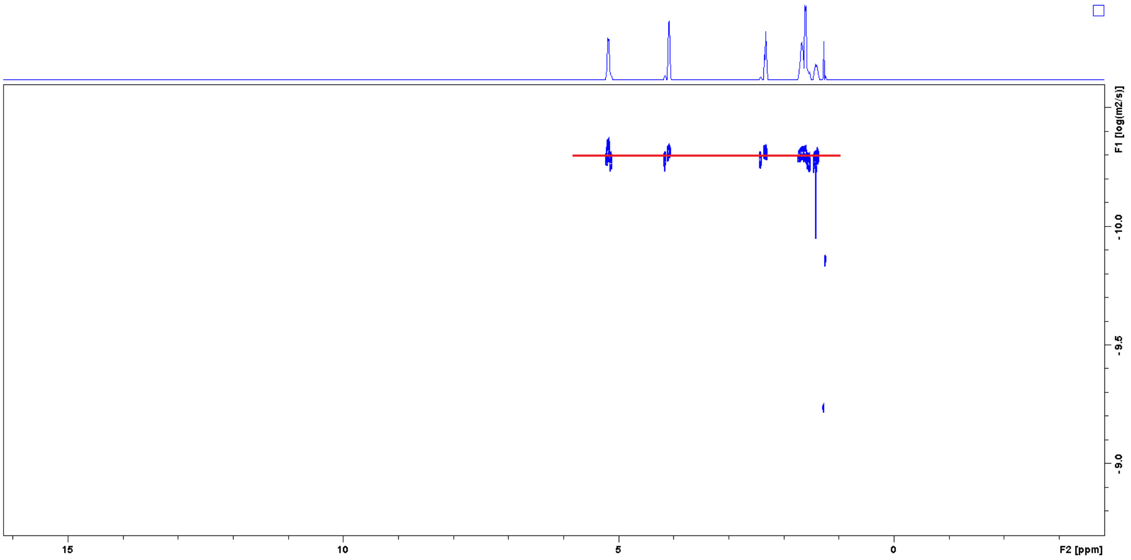

Figure S64: GPC trace of a copolymer prepared by complex **3** in toluene for DOSY analysis

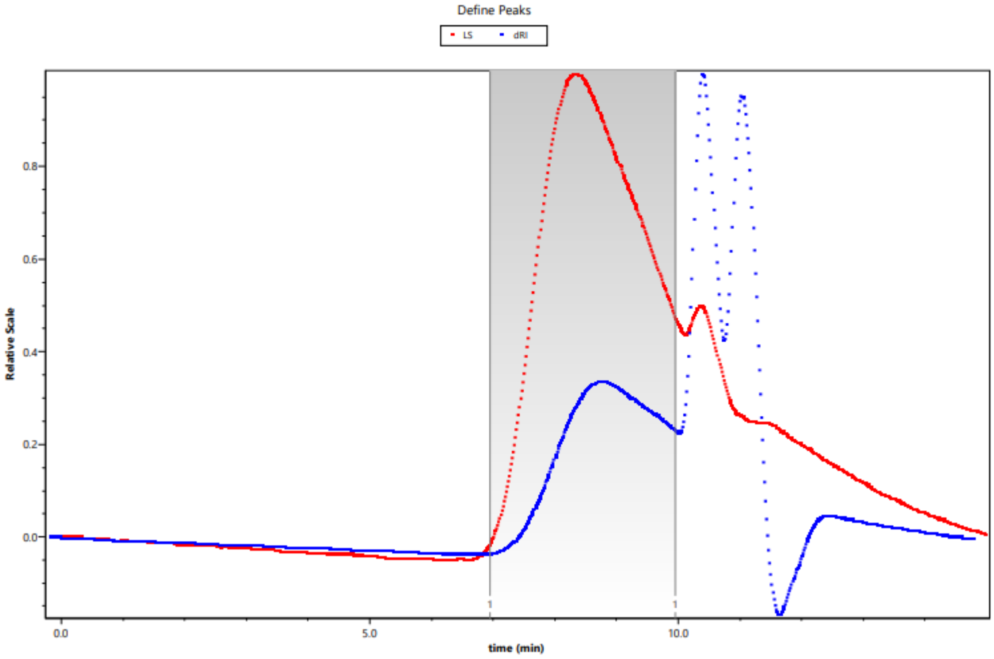

M_n = 25.3 kg mol^{-1}, $Đ$ = 1.13.

8. Calculations

Table S5: QTAIM metrics for complexes in their initial state (left) and when bound with ring-opened lactide (right).

	X=N			X=O		
	**1**	**2**	**3**	**1**	**2**	**3**
LI (La)	7.599384	7.595847	7.592705	7.578173	7.585749	7.59749
LI (N)	9.169248	9.199091	9.021658	8.736504	8.7428	8.74475
q(La)	2.795358	2.801608	2.801309	2.85028	2.854745	2.870601
q(N)	-2.84525	-2.85619	-2.78235	-1.52839	-1.53623	-1.52771
ρ	0.051083	0.053529	0.050663	0.062632	0.061917	0.056787
$\nabla^2\rho$	0.244065	0.258798	0.243448	0.433696	0.391277	0.346057
Ellipticity	0.026461	0.015466	0.018896	0.031485	0.060658	0.086993
DI(La|X)	0.319844	0.3337	0.314306	0.280889	0.256467	0.23335
NBOq(La)	1.91746	1.98176	1.89055	2.0828	2.06346	1.9976
NBOq(X)	-1.83461	-1.82818	-1.83322	-1.02607	-0.98756	-0.95968

Table S6: Percentage (%) difference in QTAIM metrics for complexes in their initial state (left) and when bound with ring-opened lactide (right), relative to complex **1**.

	X=N			X=O		
	**1**	**2**	**3**	**1**	**2**	**3**
LI (La)	0	0.046543	0.087889	0	-0.09997	-0.2549
LI (N)	0	-0.32547	1.609619	0	-0.07207	-0.09439
q(La)	0	-0.22358	-0.21289	0	-0.15665	-0.71295
q(N)	0	-0.38461	2.210529	0	-0.51283	0.044557
ρ	0	-4.78829	0.822191	0	1.141589	9.33229
$\nabla^2\rho$	0	-6.03651	0.252802	0	9.780814	20.20747
Ellipticity	0	41.55172	28.58924	0	-92.6568	-176.3
DI(La|X)	0	-4.33211	1.731469	0	8.694538	16.92448
NBOq(La)	0	-3.35339	1.403419	0	0.928558	4.090647
NBOq(X)	0	0.350483	0.075765	0	3.753155	6.470319

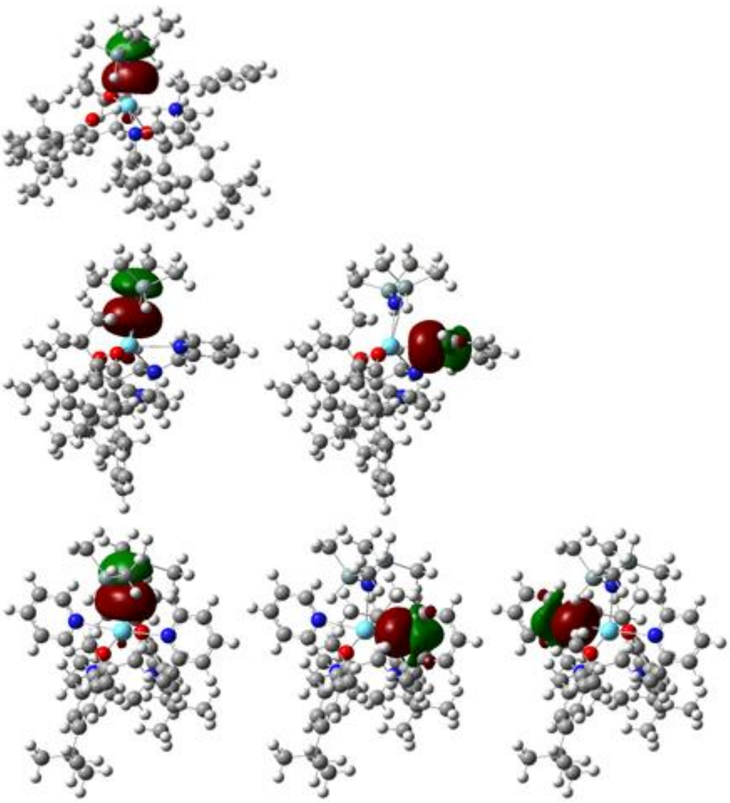

Figure S65: Natural bond orbitals representing interactions between the lanthanum ion and the ligand framework showing character from both the donating ligand atom and the lanthanum for complex **1** (top), **2** (middle), and **3** (bottom).

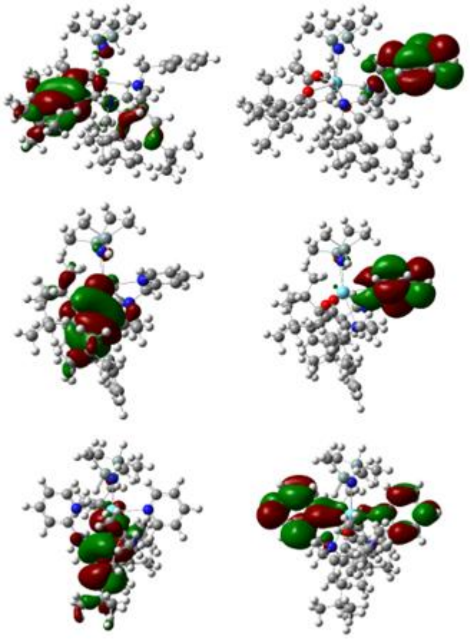

Figure S66: HOMO (left) and LUMO (right) molecular orbitals for complexes **1** (top), **2** (middle), and **3** (bottom).
